# CRISPR-Cas9 screen reveals a role of purine synthesis for estrogen receptor α activity and tamoxifen resistance of breast cancer cells

**DOI:** 10.1126/sciadv.add3685

**Published:** 2023-05-12

**Authors:** Dina Hany, Vasiliki Vafeiadou, Didier Picard

**Affiliations:** ^1^ Département de Biologie Moléculaire et Cellulaire, Université de Genève, Sciences III, Quai Ernest-Ansermet 30, CH - 1211 Genève 4, Switzerland.; ^2^ On leave from: Department of Pharmacology and Therapeutics Faculty of Pharmacy, Pharos University in Alexandria, Alexandria 21311, Egypt.

## Abstract

In breast cancer, resistance to endocrine therapies that target estrogen receptor α (ERα), such as tamoxifen and fulvestrant, remains a major clinical problem. Whether and how ERα^+^ breast cancers switch from being estrogen-dependent to estrogen-independent remains unclear. With a genome-wide CRISPR-Cas9 knockout screen, we identified previously unknown biomarkers and potential therapeutic targets of endocrine resistance. We demonstrate that high levels of PAICS, an enzyme involved in the de novo biosynthesis of purines, can shift the balance of ERα activity to be more estrogen-independent and tamoxifen-resistant. We find that this may be due to elevated activities of cAMP-activated protein kinase A and mTOR, kinases known to phosphorylate ERα specifically and to stimulate its activity. Genetic or pharmacological targeting of PAICS sensitizes tamoxifen-resistant cells to tamoxifen. Addition of purines renders them more resistant. On the basis of these findings, we propose the combined targeting of PAICS and ERα as a new, effective, and potentially safe therapeutic regimen.

## Teaser

Targeting purine biosynthesis sensitizes ERα<sup>+</sup> breast cancer cells to the gold standard therapy with tamoxifen.

## INTRODUCTION

Breast cancer remains a major threat for women worldwide. According to the latest update of global cancer statistics, female breast cancer presented the highest incidence rate with an estimated 2.3 million new cases (11.7%) in 2020, unprecedentedly surpassing lung cancer (11.4%). Among women, breast cancer has the highest 5-year prevalence (30.3%) and the highest mortality rate (15.5%) compared to other types of cancer (*1*). Breast cancer is globally classified on the basis of clinical features and molecular biomarkers. Invasive ductal carcinoma represents the most common histologic subtype of breast cancer (*2*). Molecular classification is based on the expression of the steroid receptors estrogen receptor α (ERα) and progesterone receptor (PR), the human epidermal growth factor receptor 2 (HER2), and the proliferation marker Ki-67. Luminal breast cancer that expresses ERα represents approximately 70% and includes the subtypes luminal A (ERα^+^, PR^+^, HER2^−^, and low Ki-67) and luminal B (ERα^+^, PR^+/−^, HER2_−/+_, and high Ki-67) (*2*, *3*).

ERα plays a central role in promoting survival, proliferation, invasion, and metastasis of breast cancer. ERα is a ligand-regulated transcription factor, which contains two activation functions, an N-terminal ligand-independent activation function 1 (AF-1) and a ligand-dependent activation function 2 (AF-2), which is associated with the C-terminal hormone binding domain (*4*). AF-1 and AF-2 can mediate transcriptional activation of ERα both independently and synergistically (*4*). Upon estrogen binding, ERα dissociates from molecular chaperones, dimerizes, and binds with coregulators to specific palindromic DNA sequences called estrogen response elements (EREs) leading to the induction or repression of target genes (*5*, *6*). Ligand-bound ERα can also be tethered indirectly to the response elements of other transcription factors, such as the activator protein 1 (AP-1), nuclear factor κB (NFκB), and stimulating protein 1, modulating their activities (*7*). In a nongenomic manner, the binding of estrogens to a membrane-associated subset of ERα molecules can lead to the rapid activation of various protein kinase signaling pathways, such as those involving phosphatidylinositol 3-kinase (PI3K), protein kinase B (hereafter referred to as Akt), mammalian target of rapamycin (mTOR), and mitogen-activated protein kinases (MAPKs), causing both cytoplasmic and genomic responses (*8*). ERα can also be activated as a transcription factor in the absence of estrogens by a wide variety of signal transduction pathways (see updated list at www.picard.ch/downloads/Factors.pdf), including the cyclic adenosine monophosphate (cAMP)/protein kinase A (PKA) and the PI3K/Akt/mTOR pathways (*9*, *10*). Phosphorylation of ERα occurs at multiple sites and regulates ERα functions such as sensitivity to hormones (*11*), DNA binding (*12*, *13*), and regulation of target gene transcription (*14*). It plays an important role in estrogen-dependent and estrogen-independent activation of ERα and in the response to endocrine therapy of breast cancer (*4*, *9*, *10*, *14*–*16*). For example, S305 of ERα has been shown to be the main phosphorylation target for cAMP-activated PKA, and it is associated with an enhanced agonistic behavior of tamoxifen and resistance to treatment with tamoxifen (*10*, *15*–*17*).

For decades, targeting ERα has represented the gold standard strategy for therapy of ERα^+^ breast cancer. Therapeutic targeting of ERα includes inhibition of the synthesis of estrogens using aromatase inhibitors, such as anastrozole and exemestane, competing with estrogen binding using the pure antiestrogen fulvestrant/ICI182,780 (ICI) or the partial agonist/antagonist tamoxifen. The former prevents the activation of both AF-1 and AF-2, whereas the latter inhibits the activation of AF-2 but not AF-1 (*4*). Tamoxifen represents the backbone of adjuvant endocrine therapy for both pre- and postmenopausal women with breast cancer (*18*). It has proven to be relatively well tolerated and highly efficient, although drug resistance eventually develops. As tamoxifen resistance represents a substantial problem in clinical practice, it remains a concern for basic and translational research. Many mechanisms have been described as being responsible for the escape of cancer cells from the cytotoxic effects of tamoxifen, including loss or deregulation of ERα expression and alterations of signaling pathways responsible for survival, proliferation, apoptosis, and stress responses (*9*, *10*, *15*, *16*, *19*–*21*). Metabolic rewiring plays a crucial role in cellular growth, survival, and proliferation to generate an adequate amount of adenosine triphosphate (ATP) and to fuel the biosynthesis of macromolecules in breast cancer (*22*). There is evidence supporting the role of reprogramming lipid, glucose (*23*, *24*), and amino acid (*25*, *26*) metabolism in the resistance to endocrine therapy. As an example, metabolic up-regulation in the amino acids serine and aspartate/glutamate is associated with increased nucleotide biosynthesis and resistance to tamoxifen (*25*, *26*).

Despite a substantial number of studies, failure of endocrine therapy remains a major clinical challenge. Intratumor heterogeneity (*27*) and interpatient genetic and epigenetic tumor variabilities (*28*) might contribute to the diverse responses to drugs in breast cancer (*20*). A switch of primary ERα^+^ breast cancer from being ligand-dependent to ligand-independent represents a key cause that remains mechanistically obscure. Therefore, the identification of the relevant genetic factors or biomarkers can help improve the understanding of the underlying mechanisms and hence orienting the therapeutic choices. For this study, our aim was to identify new factors that play a role in ligand-dependent and ligand-independent signaling of ERα and in the responses of breast cancer to endocrine therapy with tamoxifen or ICI. Genetic screens using the clustered regularly interspaced short palindromic repeats (CRISPR) system using the RNA-guided endonuclease Cas9 represent powerful tools for the discovery of essential genes, drug targets, or biomarkers of drug resistance (*29*–*32*). Screens with the tamoxifen-sensitive breast cancer cell lines MCF7 and T47D identified gene deletions, such as that of *CSK*, mediating estrogen-independent growth (*32*). The expression of *ARID1A* and components of the SWI/SNF complex were identified as prerequisites for the response of MCF7 cells to 4-hydroxytamoxifen (4-OHT), the major active metabolite of tamoxifen, and to ICI (*30*). A chemogenetic screen using an inhibitor of the ERα coactivator SRC-3 in MCF7 cells identified several new potential drug targets (*29*). Moreover, a specific CRISPR-Cas9 screen targeting CTCF binding sites in the vicinity of ERα-bound enhancers identified ERα target genes such as *PREX1* as being vital for ERα function (*33*). Despite all these previous efforts, a more comprehensive identification and characterization of the genetic determinants of ERα-stimulated proliferation of breast cancer cells remains to be done. We therefore performed parallel genome-wide CRISPR-Cas9 knockout screens in the absence or presence of estrogen, upon estrogen-independent activation of ERα by cAMP-activated PKA, and in the presence of the ERα antagonists 4-OHT and ICI. We used a MCF7 variant (MCF7-V) for our screens, which is relatively more 4-OHT–tolerant and displays more robust ERα responses (*34*). Our results contribute to a more complete understanding of the genes responsible for estrogen-independent and antiestrogen-resistant growth of breast cancer. Here, we highlight the role of *PAICS*, one of the top hits in our screens. It encodes a bifunctional enzyme, which is involved in the de novo biosynthesis of purine nucleotides and carries out two successive reactions as a phosphoribosylaminoimidazole carboxylase and as a phosphoribosylamino-imidazole-succinocarboxamide synthetase (*35*). Our work connects the biosynthesis and availability of purines to endocrine resistance in breast cancer.

## RESULTS

### CRISPR-Cas9 screens identify determinants of 4-OHT and ICI responses in ERα^+^ breast cancer cells

**Fig. 1.**
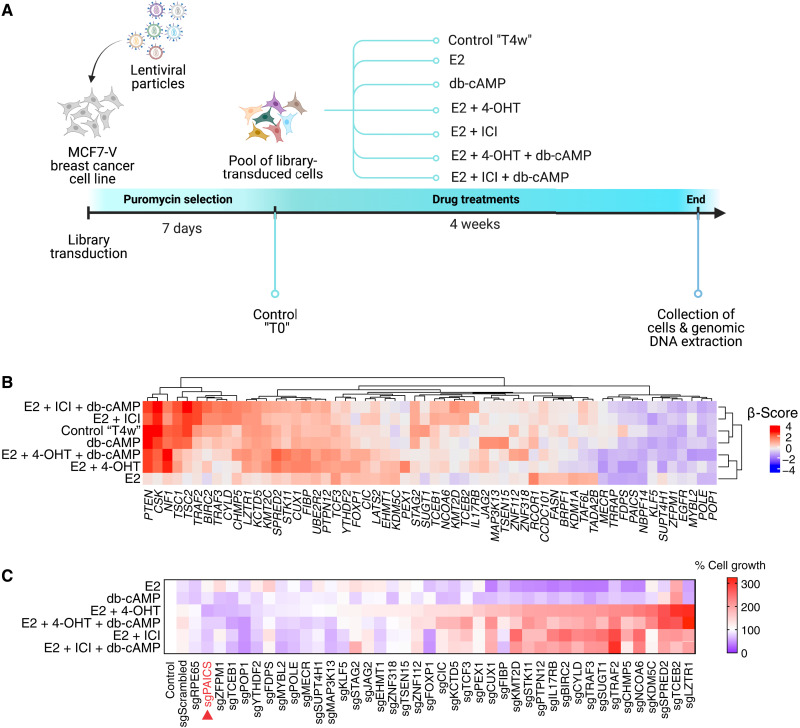
CRISPR-Cas9 screen identifies determinants of 4-OHT and ICI responses in ERα^+^breast cancer cells. (**A**) Schematic representation and timeline of the primary CRISPR-Cas9 screen. Cells collected before drug treatments at time 0 (control T0) and vehicle-treated cells collected after 4 weeks (control “T4w”) were used as controls. The scheme was created with Biorender.com. (**B**) Heatmap representation of the hierarchical cluster analysis done for the top 58 genes identified by the primary CRISPR-Cas9 screen. Values are based on the calculated β-score of the pooled sgRNA reads from two biological replicates. Red indicates enrichment, and blue indicates depletion of the sgRNAs of the corresponding genes. Cutoff value: β-score ≤ −0.3 or ≥ 0.3, and FDR ≤ 0.1 or ≥ −0.1. (**C**) Heatmap representation of % cell growth of individual knockouts of 40 genes selected for a secondary screen. Cell growth was measured by a crystal violet assay. Absorbances from each knockout cell line were first normalized to vehicle-treated control and then to the corresponding values of nontransduced WT MCF7-V cells (set to 100%). Cells transduced with sgScrambled (expressing a scrambled sequence) or sgRPE65 (expressing a sgRNA targeting an unexpressed gene) represent two additional control cell lines. Values represent averages of three independent experiments. Red and blue indicate increased and reduced cell growth, respectively. Values were ordered ascendingly based on % cell growth with 4-OHT. Red arrow head indicates sgPAICS as the knockout cell line with the highest sensitivity to 4-OHT.

We used a genome-wide CRISPR-Cas9 knockout screen approach, in which 17β-estradiol (E2) and dibutyryl-cAMP (db-cAMP), a cell-permeable version of cAMP, were used as inducers of cell proliferation, whereas 4-OHT and ICI were used as inhibitors of cell proliferation of MCF7-V cells (fig. S1, A and B). We used the human Brunello CRISPR knockout pooled library, which targets 19,114 functional genes of the human genome with three or four single-guide RNAs (sgRNAs) each (*36*). The library was amplified and validated for its complexity by next-generation sequencing (NGS) (fig. S1, C and D). A pool of viral particles was prepared to infect MCF7-V cells (Fig. 1A and fig. S1D). Transduced cells were then selected with puromycin, pooled, and subjected to the following conditions: only vehicle (for the negative control), E2, db-cAMP, E2 + 4-OHT, E2 + ICI, E2 + 4-OHT + db-cAMP, and E2 + ICI + db-cAMP (Fig. 1A and fig. S1, B and D). A low concentration of E2 of 100 pM was used to induce ERα-mediated cell proliferation, whereas ERα antagonists were used at 100 nM, a dose that is sufficiently high to counteract E2 (fig. S1, A and B) and yet relatively low to avoid unspecific effects. Before treatments, one sample of the pooled cells was collected to represent the control condition (control “T0”) (Fig. 1A and fig. S1D). Note that our choice of treatments allows both positive and negative selections. For example, in the case of E2 and db-cAMP, which both enhance the growth and proliferation of ERα^+^ MCF7-V cells (fig. S1, A and B), a gene whose knockout impairs proliferation and hence is negatively selected can be considered as activator of the E2 or db-cAMP growth stimulatory pathways. In contrast, in the case of growth inhibitors like 4-OHT and ICI, a gene whose knockout causes the cells to survive the inhibitory effects of the drugs (i.e., is positively selected) might be responsible for drug sensitivity. By NGS, we identified the enrichment and depletion of sgRNAs for each treatment condition. Normalized counts of sgRNAs and the calculated β-scores are available in separate files (data S1 and S2, respectively). There are approximately 600 genes whose sgRNA sequences were strongly depleted regardless of the treatment. We removed them as “essential genes” (data S3) from further analyses. Next, a set of 58 genes was identified and hierarchically clustered into 15 distinct groups (Fig. 1B and data S4). They represent the top hits that show significant enrichment or depletion of sgRNA sequences in each condition as compared to the vehicle-treated control. Some of the top hits identified by our screen, including *PTEN*, *CSK*, *NF1*, and *TSC1/2* had previously been identified by a CRISPR-Cas9 screen that was performed with the ERα^+^ breast cancer cell lines MCF7 and T47D (*32*). On the basis of literature curation, a subset of 40 genes with potentially novel functions in the context of ERα^+^ breast cancer was identified and selected for further validation through a secondary screen. For each gene, we selected the sequence of one sgRNA present in the pooled Brunello library to produce a single knockout of the corresponding gene in MCF7-V cells. A polyclonal pool of knockout cells for each gene was subjected to the same treatments as in the primary screen, and the results were represented as a heatmap of cell growth (Fig. 1C). This analysis confirmed that, of the 40 selected genes, 33 might play a role in ERα signaling and/or 4-OHT or ICI responses. For example, loss of function of *LZTR1*, *TCEB2*, and *SPRED2* caused the cells to be significantly more resistant to 4-OHT and, to a lesser extent, to ICI (Fig. 1, B and C). We have recently shown that *SPRED2* deficiency is an important indicator of tamoxifen resistance because of ERα hyperactivation through the MAPK signaling pathway (*37*). Moreover, the loss of functions of *BIRC2*, *TRAF2*, *TRAF3*, *CYLD*, and *CHMP5*, which all encode factors involved in tumor necrosis factor–α–mediated activation of NFκB, was associated with significant resistance to ICI and, to a lesser extent, to 4-OHT (Fig. 1, B and C). In contrast, the loss of function of another set of genes, such as *PAICS*, *ZFPM1*, and *POP1*, enhanced the sensitivity to 4-OHT/ICI treatments (Fig. 1, B and C). We decided to focus on PAICS for the following.

### PAICS is up-regulated in cancer and correlates with progression and poor clinical outcomes of ERα^+^ breast tumors

PAICS, a dual enzyme involved in the de novo biosynthesis of purines, appeared as the top hit of knockouts that sensitize MCF7-V cells to 4-OHT and ICI. The sgRNAs for the genes encoding the other enzymes of the same pathway, including *PPAT*, *ATIC*, *ADSL*, *PFAS*, and *GART*, were depleted regardless of the treatment and therefore filtered out (data S2), and in the case of *GART* even as an essential gene (data S3). Here, note that the CRISPR-Cas9 screen did not show any significant differential effects (data S1 and S2) for the knockouts of the genes *CAD*, *DHODH*, and *UMPS*, which encode enzymes involved in the de novo biosynthesis of the pyrimidine nucleotides uridine monophosphate and cytidine monophosphate, nor for *DHFR* and *TYMS*, which encode enzymes involved in the conversion of folate to deoxythymidine monophosphate.

**Fig. 2.**
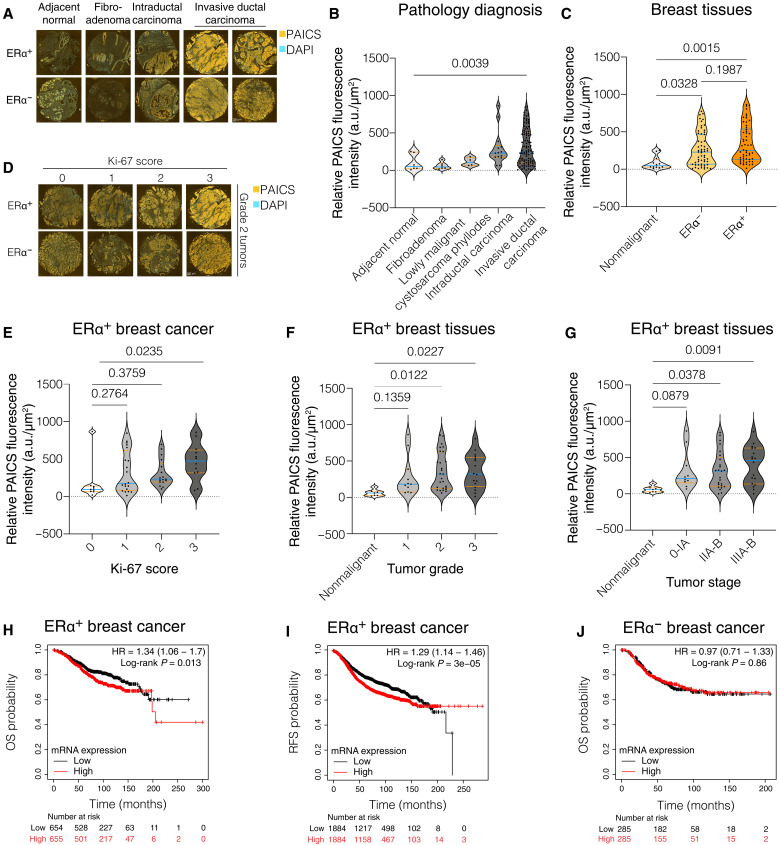
PAICS is up-regulated in cancer and correlates with progression and poor clinical outcomes of ERαbreast tumors. (**A** and **D**) Representative images from the TMA analysis of human breast tissue cores immunostained for PAICS (orange) and counterstained for the nuclei with 4′,6-diamidino-2-phenylindole (DAPI) (blue). Representative images of the pathological diagnosis (A) and Ki-67 score (D) among ERα^+^ and ERα^−^ breast samples. (**B**, **C**, and **E** to **G**) Violin plots of the means of the relative fluorescence intensities of PAICS [in arbitrary units (a.u.)] per fluorescent unit area (in square micrometers) and its 95% confidence interval between cores, classified on the basis of pathology diagnosis (B), malignancy and ERα status (C), Ki-67 score of ERα^+^ breast cancer (E), tumor grade in ERα^+^ breast tissues (F), and TNM stage in ERα^+^ breast samples (G). The statistical significance between the groups was analyzed by one-way analysis of variance (ANOVA), and *P* values of <0.05 were considered statistically significant. (**H** to **J**) Kaplan-Meier plots for overall survival (OS) (H) and relapse-free survival (RFS) (I) of ERα^+^ breast cancer, and OS (J) in ERα^−^ ones, classified as tumors expressing high levels (red line) and low levels (black line) of *PAICS* mRNA. HR, hazard ratio.

To determine whether PAICS protein levels correlate with oncogenic characteristics, we compared them across multiple breast cancer cell lines (MCF7, MCF7-V, T47D, H3396, BT474, SKBR3, and MDA-MB-231) and the noncancerous breast epithelial cell line MCF10A (fig. S2A). In addition, we compared several isogenic pairs of oncogenically transformed nonbreast cell lines and their noncancer parent derivatives. These included the nontransformed retinal pigment epithelial cell line RPE1, immortalized with human telomerase reverse transcripase (hTERT), along with its counterpart transformed by the H-Ras mutant G12V (RPE1 Ras) (fig. S2B), the nontumorigenic lung epithelial cell line BEAS-2B and its v-Ha-Ras–transformed derivative BZR (fig. S2C), and human skin fibroblasts that were sequentially transformed by the stable overexpression of human telomerase (hTERT), hTERT + SV40 large T antigen (hTERT LT), and hTERT + SV40 large T antigen + H-RasG12V (hTERT LT Ras) (fig. S2D) (*38*, *39*). We consistently observed higher PAICS protein levels in cancer cell lines as compared to their respective noncancerous counterparts (fig. S2, A to D). The specific correlation between PAICS expression and breast cancer was further investigated using a human breast tissue microarray (TMA). Fluorescent immunostaining of the PAICS protein showed elevated expression in malignant breast tissues including intraductal and invasive ductal carcinomas as compared to nonmalignant tissue biopsies including adjacent normal breast tissues and fibroadenomas, in both ERα^+^ and ERα^−^ samples (Fig. 2, A to C). PAICS expression showed a strong correlation with the tumor progression based on the pathological diagnosis (Fig. 2, A and B), the expression of the cell proliferation marker Ki-67 (Fig. 2, D and E, and fig. S2, E and F), the tumor grade (Fig. 2F and fig. S2, G and H), and the “tumor, node, and metastasis” (TNM) stage (Fig. 2G and fig. S2, I and J). More specifically, statistically significant correlations were associated with ERα^+^ (Fig. 2, E to G) rather than ERα^−^ tumors (fig. S2, F, H, and J).

Next, we used the Kaplan-Meier plotter (*40*) to search for the correlation between *PAICS* mRNA expression levels and survival of patients with breast cancer. We found that *PAICS* gene expression is significantly correlated with poor overall survival (OS) and relapse-free survival (RFS) of patients with ERα^+^ breast tumors (Fig. 2, H and I) and specifically also of tamoxifen-treated patients (figs. S2K and S3A). We extended this analysis to all other components of the purinosome complex. High mRNA levels of three of the five other enzymes (PPAT, ATIC, and ADSL) are significantly associated with poor RFS in patients with ERα^+^ breast cancer treated with tamoxifen (figs. S3 and S4). In contrast, there is no correlation between high mRNA levels of any of the six enzymes with improved RFS of patients with ERα^−^ breast cancer (Fig. 2J and figs. S3 and S4). Overall, these results suggest that PAICS is highly expressed in cancer and can be a marker of breast tumor progression and that four enzymes of the purinosome complex, including PAICS, may determine disease outcomes, particularly in patients with ERα^+^ breast cancer treated with tamoxifen.

### PAICS expression correlates with cell migration and estrogen-independent and tamoxifen-resistant proliferation of ERα^+^ breast cancer cells

**Fig. 3.**
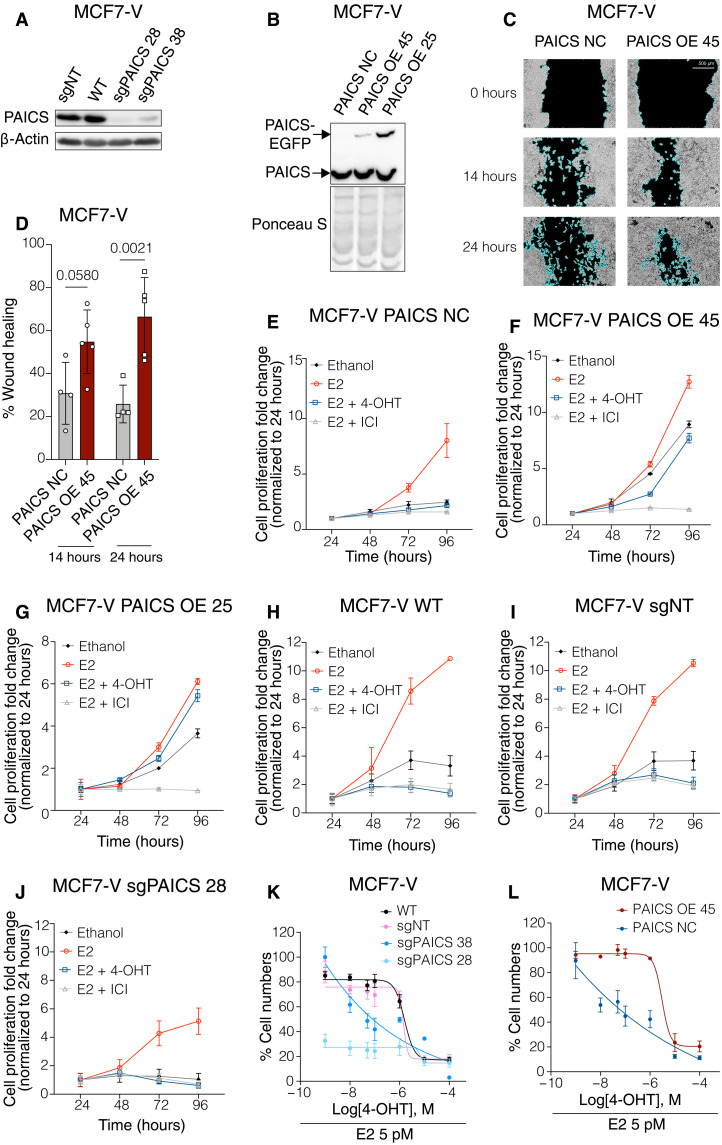
PAICS expression correlates with cell migration and estrogen-independent and tamoxifen-resistant proliferation of ERα^+^breast cancer cells. (**A** and **B**) Immunoblots of PAICS showing the efficiency of its knockdown (A) and overexpression (B) in MCF7-V cells. In (A), MCF7-V WT and sgNT (sgRPE65) cells are negative controls used interchangeably. β-Actin is used as an internal control. In (B), a transduced clone that did not show PAICS overexpression (PAICS NC) is used as a negative control. The PAICS-EGFP fusion protein is detected at about 77 kDa. Ponceau S staining is used as a protein loading control. (**C**) Cell migration assay of MCF7-V cells without and with PAICS overexpression. Representative phase-contrast images for all replicates (including these) are presented in fig. S5F. (**D**) Bar graph showing the % wound healing of MCF7-V cells presented in fig. S5F. (**E** to **J**) Proliferation of the indicated MCF7-V clones treated with either vehicle (ethanol), E2, E2 + 4-OHT, or E2+ ICI. (**K** and **L**) Dose-response curves with increasing doses of 4-OHT with PAICS-deficient (K) and PAICS-overexpressing clones (L). For each curve, the result for a vehicle-treated control group was set to 100%. For (D) to (L), data are represented as means ± SD of at least three independent experiments. Statistical significance in (D) was analyzed by two-way ANOVA, and *P* values of <0.05 were considered statistically significant.

To explore whether *PAICS* can function as an oncogene, we stably overexpressed a PAICS–enhanced green fluorescent protein (EGFP) fusion protein in the noncancerous RPE1 cell line (fig. S5A). RPE1 cells overexpressing PAICS display more irregular, spreading, and protrusive shapes than the parent cells (fig. S5B). In a wound-healing assay, PAICS overexpression significantly enhanced cell migration and fastened healing of the wounded area as compared to wild-type (WT) nontransfected RPE1 (fig. S5, C and D). For further characterization with ERα^+^ breast cancer cells, we generated two individual knockdown clones of PAICS in MCF7-V cells using a CRISPR-Cas9 sgRNA lentiviral construct targeting *PAICS* (sgPAICS) (Fig. 3A). In parallel, cells were transduced with lentiviral particles for the expression of a sgRNA targeting the unexpressed gene *RPE65* as a negative control (sgNT). Because we could not identify a full knockout clone of PAICS, we hypothesize that some low level of PAICS may be essential for survival, similarly to the other members of the de novo purine biosynthesis pathway, such as GART (data S3). Two MCF7-V clones with stable overexpression of PAICS-EGFP were generated using the expression plasmid pPAICS-EGFP. These cellular clones were referred to as PAICS OE 45 and PAICS OE 25 (Fig. 3B). Another clone that was obtained in parallel but was not showing overexpression of PAICS by immunoblotting was used as a negative control (PAICS NC). Similarly, two PAICS-overexpressing clones were obtained in T47D cells and are referred to as PAICS OE 2 and PAICS OE 8 (fig. S5E). Reminiscent of the results obtained with PAICS-overexpressing RPE1 cells, MCF7-V cells showed enhanced cell migration when PAICS was overexpressed (Fig. 3, C and D, and fig. S5F), whereas no significant difference to WT cells was observed in the case of the knockdown of PAICS (fig. S5, G and H). Next, we determined more specifically whether the differential expression of PAICS affects the proliferation rate of ERα^+^ breast cancer cells. We found that its overexpression increases the proliferation of MCF7-V and T47D cells in the absence of E2 and in the presence of 4-OHT, but not ICI, by comparison with PAICS NC cells (Fig. 3, E to G, and fig. S5, I to K). PAICS deficiency caused MCF7-V cells to become more dependent on E2 for their proliferation (Fig. 3, H to J). Together, these results indicate that increased PAICS expression may stimulate the proliferation and migration of ERα^+^ breast cancer cells in the absence of estrogen, whereas PAICS deficiency restricts cell proliferation to the presence of estrogen.

To evaluate the effect of the differential protein expression levels of PAICS on the response to 4-OHT, we treated WT untransfected MCF7-V cells, a pool of sgNT clones, the two clones that are deficient in PAICS, the two clones overexpressing PAICS, and PAICS NC cells with increasing doses of 4-OHT. Knockdown of PAICS resulted in an increased sensitivity to 4-OHT as compared to either WT or sgNT cells (Fig. 3K). By comparing the approximate concentration required for 50% inhibition (IC_50_), we found that PAICS deficiency resulted in an enhanced potency of 4-OHT by about 12- and 4000-fold in sgPAICS 38 and sgPAICS 28 knockdown clones, respectively, as compared to sgNT cells (Fig. 3K). Conversely, overexpression of PAICS led to a 4-OHT–resistant phenotype when compared to PAICS NC cells (Fig. 3L and fig. S6, A to D). Overexpression of PAICS reduced the potency of 4-OHT by about 50-fold in PAICS OE 45 cells as compared to PAICS NC cells in the MCF7-V background (Fig. 3L). Similar increases in the IC_50_ were observed with the other overexpression clone (PAICS OE 25) and with the PAICS-overexpressing T47D clones (fig. S6, A to D). Hence, PAICS expression levels are directly correlated with resistance of ERα^+^ breast cancer cells to 4-OHT treatment.

We next investigated whether the mere addition of purine nucleosides to medium complemented with purine-depleted serum would result in resistance to 4-OHT. We tested the impact of adenosine, guanosine, inosine, and the combination of all three on proliferation in the presence of 4-OHT. We found that the depletion of purines resulted in enhanced sensitivity to 4-OHT and that adding back purine nucleosides essentially restored the initial response to E2 and to E2 + 4-OHT (fig. S6, E to H). Thus, the overexpression of PAICS or the addition of purine nucleosides makes cells relatively more 4-OHT–resistant.

### Interplay between PAICS and ERα

**Fig. 4.**
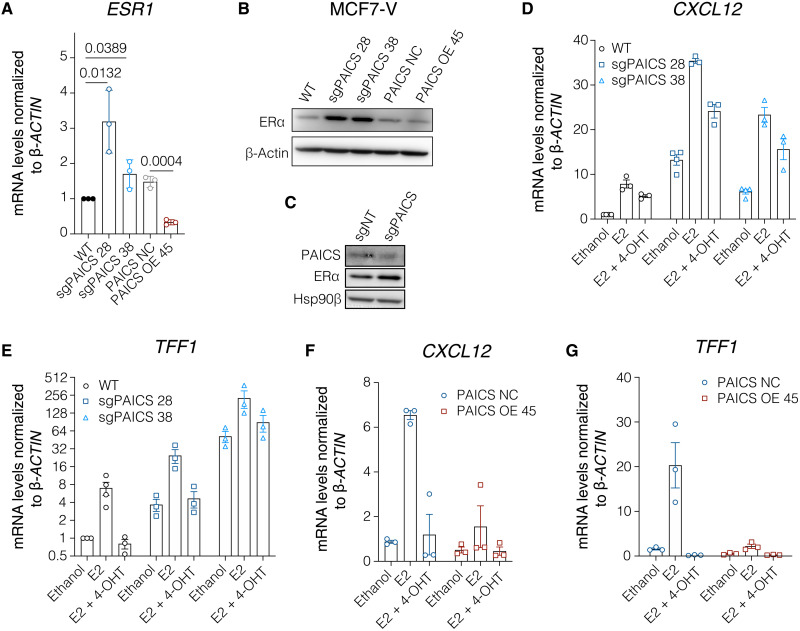
Interplay between PAICS and ERα. (**A**) mRNA levels of the ERα gene *ESR1* measured by RT-qPCR in MCF7-V cells as indicated. Ct values were first normalized to β*-ACTIN*, and then values corresponding to WT were set to 1. (**B**) Immunoblot of ERα in total cell lysates. (**C**) Immunoblots of ERα and PAICS in total cell lysates of MCF7-V cells transiently transfected as indicated. Hsp90β was used as loading control. (**D** to **G**) mRNA levels of the ERα target genes *CXCL12* and *TFF1* in indicated MCF7-V cells; E2 was used at 1 nM. For (D) and (E), values corresponding to WT treated with vehicle were set to 1. For (F) and (G), values corresponding to PAICS NC treated with vehicle were set to 1. For bar graphs, data are represented as means ± SD of at least three independent experiments. For (A), statistical significance between the groups was analyzed by unpaired Student’s *t* tests (A), and *P* values of <0.05 were considered statistically significant.

Because we had found that increased PAICS expression levels promote estrogen-independent proliferation of MCF7-V and T47D cells (Fig. 3, E to G, and fig. S5, I to K), and resistance to 4-OHT (Fig. 3, E to G, K, and L, and fig. S5, I to K), we wondered whether PAICS affects expression of ERα and what role it plays in ERα signaling and tamoxifen resistance. We determined ERα expression at the mRNA (*ESR1*) and protein levels and found that PAICS deficiency in MCF7-V cells resulted in an increase in the basal *ESR1* mRNA and ERα protein levels as compared to parent MCF7-V cells (Fig. 4, A to C). This increase of ERα levels is associated with an increase in the basal and E2-induced transcription of the ERα target genes *CXCL12*, *TFF1*, and *XBP1* (Fig. 4, D and E, and fig. S7A). In contrast, overexpression of PAICS significantly diminished *ESR1* mRNA and ERα protein levels in MCF7-V (Fig. 4, A and B). Similarly, minimal ERα protein levels were observed in T47D when PAICS was overexpressed (fig. S7B). This down-regulation was also associated with a diminished basal and E2-induced transcription of the ERα target genes *CXCL12*, *TFF1*, and *XBP1* in MCF7-V (Fig. 4, F and G, and fig. S7A). To verify that the reduction of ERα levels by PAICS overexpression is not due to a nonspecific clonal effect, we transfected PAICS OE 45 and PAICS OE 25 cells with an expression vector for sgPAICS. The depletion of both endogenous and exogenous PAICS up-regulated ERα protein levels (fig. S7C). These results demonstrate that there is an inverse correlation between PAICS and ERα expression at both the mRNA and protein levels.

**Fig. 5.**
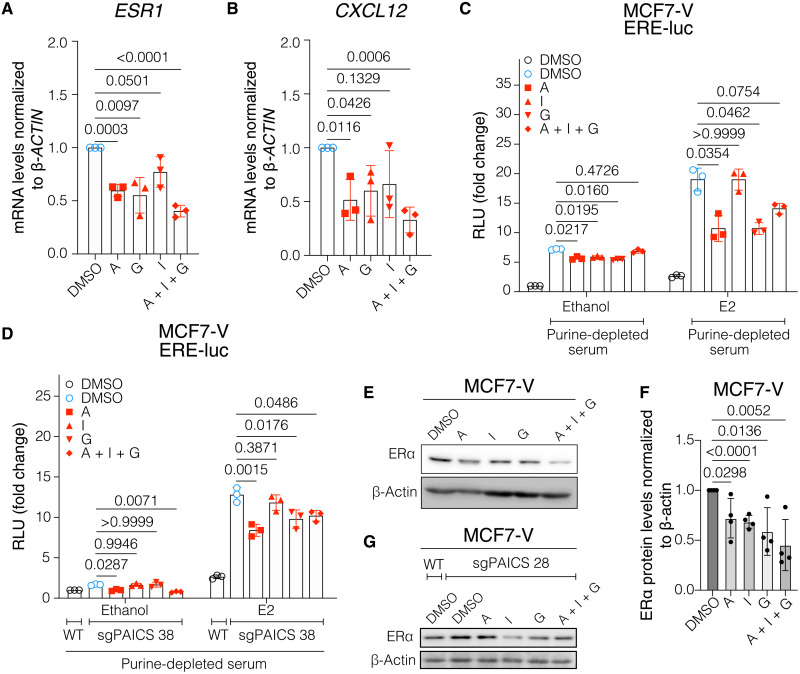
Purine nucleosides reduce ERα expression levels. (**A** and **B**) Normalized mRNA levels of *ESR1* and *CXCL12* in MCF7-V cells starved in purine-depleted medium for 72 hours and then treated with either dimethyl sulfoxide (DMSO), A (5 μM adenosine), G (5 μM guanosine), I (5 μM inosine), or a combination of the three nucleosides (A + I + G), each at 5 μM. Values corresponding to vehicle were set to 1. (**C** and **D**) Luciferase reporter assays for endogenous ERα activity in MCF7-V WT (C) and sgPAICS 38 (D) cells. Cells were purine-starved and then treated as indicated. In parallel, for (C), a control was incubated in purine-rich medium and treated with DMSO (with or without E2). Values of the control treated with vehicle (ethanol) were set to 1. For (D), a control of nontransduced WT MCF7-V cells was purine-starved and then treated with DMSO (with or without E2). Values of the control group treated with ethanol were set to 1. RLU, relative luminescence unit. (**E**) Representative immunoblot of ERα in MCF7-V WT cells, hormone-starved for 72 hours, and then treated with the indicated nucleosides, each at 100 μM. (**F**) Quantification of ERα protein levels from four replicate blots as in (E). (**G**) Immunoblot of ERα in sgPAICS 28 cells, treated as in (E). For bar graphs, data are represented as means ± SD of at least three independent experiments. Statistical significance was analyzed by unpaired Student’s *t* tests (A), (B), and (F) or two-way ANOVA (C) and (D), and *P* values of <0.05 were considered statistically significant.

Considering the biochemical functions of PAICS, it might also affect ERα expression through its impact on purine biosynthesis. To explore the mechanism, we cultured WT MCF7-V cells in medium with purine-depleted serum supplemented with either adenosine, guanosine, inosine, or a combination thereof. We observed that the expression of *ESR1* itself and of the ERα target genes *CXCL12*, *GREB1*, and *XBP1* is reduced by added adenosine and guanosine nucleosides but less consistently by inosine (Fig. 5, A and B, and fig. S7, D and E). We also determined the impact of purines on ERα transcriptional activity with a luciferase reporter assay using the ERE-luciferase reporter ERE-Luc. Incubation with medium containing purine-depleted serum was associated with an induction of basal and E2-induced ERα activity, and the addition of nucleosides reduced activity to varying extents (Fig. 5C). A very similar pattern could be seen upon depletion of PAICS (Fig. 5D). The addition of the three nucleosides, notably in combination, caused the down-regulation of ERα in MCF7-V and T47D cells (Fig. 5, E and F, and fig. S7, F and G). Similar results were obtained with the PAICS-deficient MCF7-V clones (Fig. 5G, and fig. S7H). Hence, both ERα expression and activity are affected by the availability of purine nucleotides.

We speculated that the up-regulation and increased ERα availability and activity in the case of a PAICS deficiency could sensitize cells to 4-OHT and that PAICS overexpression could have the opposite effect. To confirm our hypothesis, we simulated the effect of a PAICS deficiency on ERα levels by overexpression of full-length ERα in MCF7-V cells (fig. S7I). A dose-response analysis of ERα-overexpressing clones with 4-OHT showed a significant enhancement of potency by about 9- and 16-fold for ERα overexpression clones OE 17 and OE 24, respectively (fig. S7, J and K). Therefore, PAICS expression is a determinant of ERα mRNA and protein expression and influences the cellular responses to ERα agonists and inhibitors, such as tamoxifen.

### Increased expression of PAICS correlates with cAMP/PKA activation in ERα^+^ breast cancer cells

**Fig. 6.**
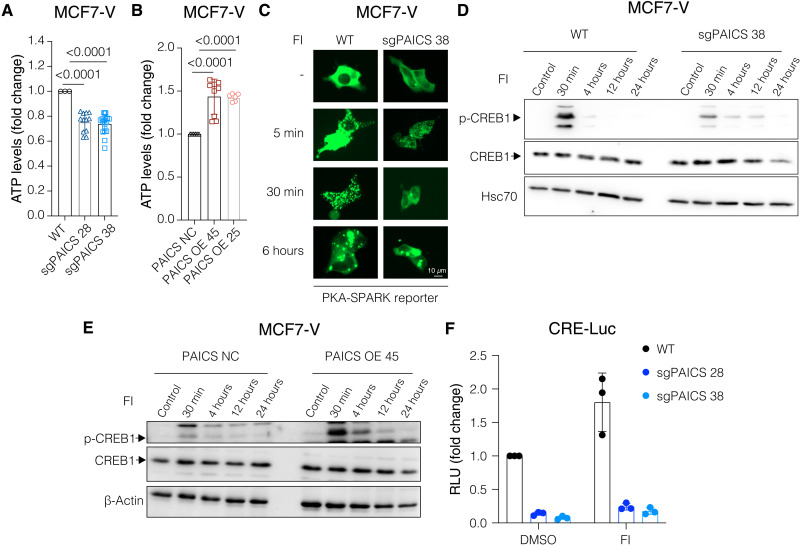
Increased expression of PAICS correlates with activation of cAMP/PKA signaling in ERα^+^breast cancer cells. (**A** and **B**) ATP levels measured using CellTiter-Glo reagent of equal numbers of cells. Luminescence signal of MCF7-V WT (A) or PAICS NC (B) was set to 1. (**C**) Representative images of cells transiently transfected with the PKA-SPARK reporter plasmid. Cells were treated with FI for 5 min, 30 min, and 6 hours to induce cAMP/PKA signaling. Green fluorescent phase separation droplets are indicative of active PKA. Images for all replicates (including these) are presented in fig. S8D. (**D** and **E**) Immunoblots of phosphorylated (S133) and total CREB1 in total cell lysates. Cells were treated with FI for 30 min, 4 hours, 12 hours, and 24 hours to induce the phosphorylation of CREB1 downstream of active PKA. (**F**) Luciferase reporter assay for endogenous CREB1 activity in the indicated MCF7-V cells transiently transfected with the CRE-Luc reporter plasmid. Cells were hormone-starved for 72 hours and then treated with either vehicle (DMSO) or FI. Luciferase activities of MCF7-V WT cells treated with vehicle were set to 1. Data are represented as means ± SD of at least three independent experiments. For (A) and (B), statistical significance between the groups was analyzed by unpaired Student’s t-tests, and *P* values of <0.05 were considered statistically significant.

Because PAICS deficiency would impair the synthesis of purine nucleotides, including adenosine monophosphate (AMP), we expected a decrease in the pool of ATP and hence a reduction in cAMP levels. By measuring the basal ATP levels in PAICS-deficient (Fig. 6A) and PAICS-overexpressing MCF7-V cells (Fig. 6B), we found that PAICS expression levels are directly correlated with ATP levels (Fig. 6, A and B). A similar increase in ATP was associated with PAICS-overexpression in RPE1 cells (fig. S8A). Then, we directly determined the induced levels of cAMP upon treatment with the adenylate cyclase activator forskolin and the phosphodiesterase inhibitor 3-isobutyl-1-methylxanthine (IBMX), a cocktail hereafter referred to as “FI.” In the case of a PAICS deficiency, both the peak and the cAMP levels after 6 hours were lower than that in the sgNT control cells (fig. S8B). Conversely, the overexpression of PAICS resulted in higher sustained levels of cAMP (fig. S8C). Modulation of cAMP levels would affect the extent of PKA activation and the phosphorylation of its downstream substrates, including the transcription factor cAMP response element (CRE)–binding protein 1 (CREB1). Activation of CREB1 by phosphorylation is known to contribute to increased growth and survival of cancer cells (*41*, *42*). We determined PKA activity using the PKA-SPARK (separation of phases-based activity reporter of kinase) (*43*). This system enabled us to visualize PKA activity as GFP-based phase separation droplets. A significant reduction in PKA activity at both the basal and FI-induced levels was observed in PAICS-deficient cells compared to WT (Fig. 6C and fig. S8D). To assess the phosphorylation of the key PKA substrate CREB1, FI-induced CREB1 phosphorylation was visualized by immunoblotting with a phosphoserine-specific antibody. We found that PAICS deficiency was associated with reduced CREB1 phosphorylation upon treating cells with FI (Fig. 6D and fig. S8E) and that adding adenosine restored its phosphorylation (fig. S8F). Moreover, overexpression of PAICS augmented both the initial and the sustained phosphorylation of CREB1 (Fig. 6E). In a luciferase reporter assay for CREB1, deficiency of PAICS led to a significant reduction in CRE activity both at the basal and FI-induced levels (Fig. 6F). Therefore, PAICS expression determines the levels of ATP, the induced levels of cAMP, and the activity of PKA and its substrates.

**Fig. 7.**
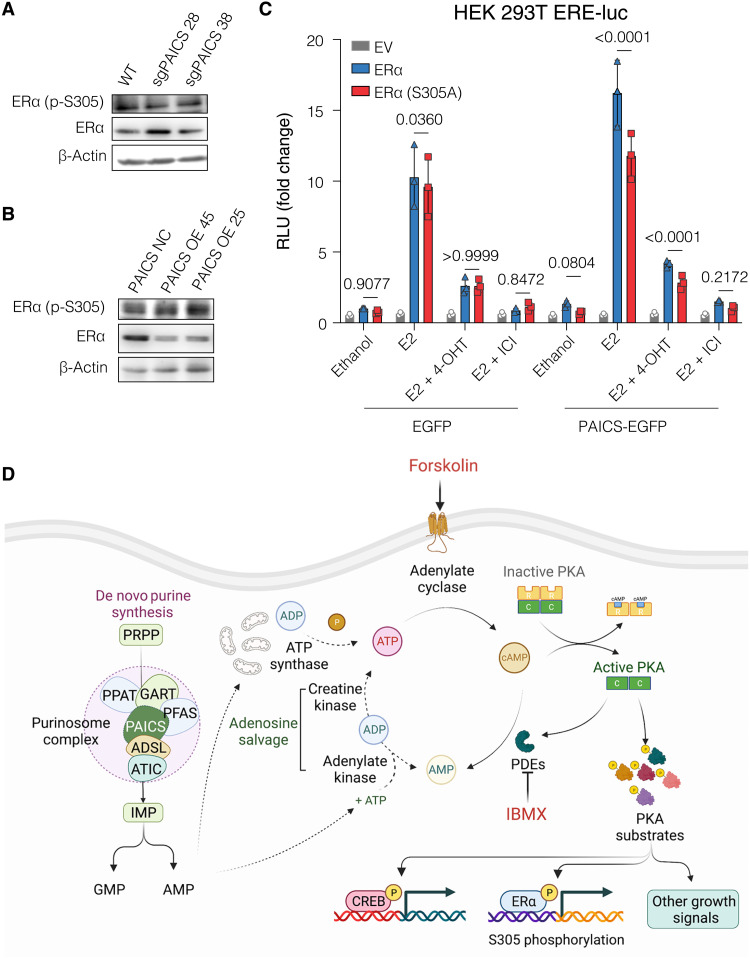
High PAICS levels are associated with increased dependence of ERα activity on S305. (**A** and **B**) Immunoblots of phosphorylated (S305) and total ERα. (**C**) Luciferase reporter assay for exogenously expressed ERα in HEK 293 T cells transiently cotransfected with the ERE-Luc reporter, and either the plasmid PAICS-EGFP for PAICS overexpression or the empty vector pEGFP-C1 (EGFP) as a control. In addition, transfection mixtures contained either empty vector pSG5 (EV), HEG0 (ERα), or the corresponding plasmid for expression of ERα S305A. The relative luciferase activities (RLU) are relative to the activities of the internal transfection standard Renilla luciferase. Cells were hormone-starved for 72 hours and then treated with either ethanol, E2 (1 nM), or E2 (1 nM) + 4-OHT (100 nM), or E2 (1 nM) + ICI (100 nM) for 24 hours. Luciferase activities of the control cells transfected with ERα and treated with vehicle was set to 1. Data are represented as means ± SD of at least three independent experiments. Statistical significance was analyzed by two-way ANOVA, and *P* values of <0.05 were considered statistically significant. (**D**) Schematic illustration summarizing the influence of PAICS levels on cAMP/PKA signaling. PDEs, phosphodiesterase enzymes. The scheme was created with Biorender.com.

Activation of cAMP/PKA promotes ERα activity both in the presence and in the absence of estrogen or tamoxifen. Phosphorylation of ERα by PKA at different sites, including S305, contributes to its activation and tamoxifen resistance (*15*, *16*, *44*). Therefore, we speculated that overexpression of PAICS might promote the phosphorylation of S305 of ERα, which might contribute to the observed estrogen-independent growth and resistance to tamoxifen (Fig. 3, E to G, K, and L, and fig. S5, I to K). Immunoblotting for ERα phospho-S305 showed that PAICS deficiency is associated with reduced phosphorylation of ERα at S305, whereas the overexpression of PAICS correlates with hyperphosphorylation at this site. This is particularly obvious when one considers the fact that total ERα levels are inversely affected by PAICS (Fig. 7, A and B, and figs. S7B and S8, G and H). Further validation using an ERE-Luc reporter assay in human embryonic kidney (HEK) 293 T cells revealed that overexpression of PAICS shifted the activity of ERα to be more dependent on S305, because the E2-induced transcriptional activity of the S305A mutant was reduced compared to that of WT ERα (Fig. 7C). These results are consistent with the conclusion that the expression of PAICS is directly correlated with phosphorylation of ERα at S305, which contributes to tamoxifen resistance (Fig. 7D) (*15*, *16*, *44*).

### Expression of PAICS is correlated with mTOR activity and the global rate of protein translation

**Fig. 8.**
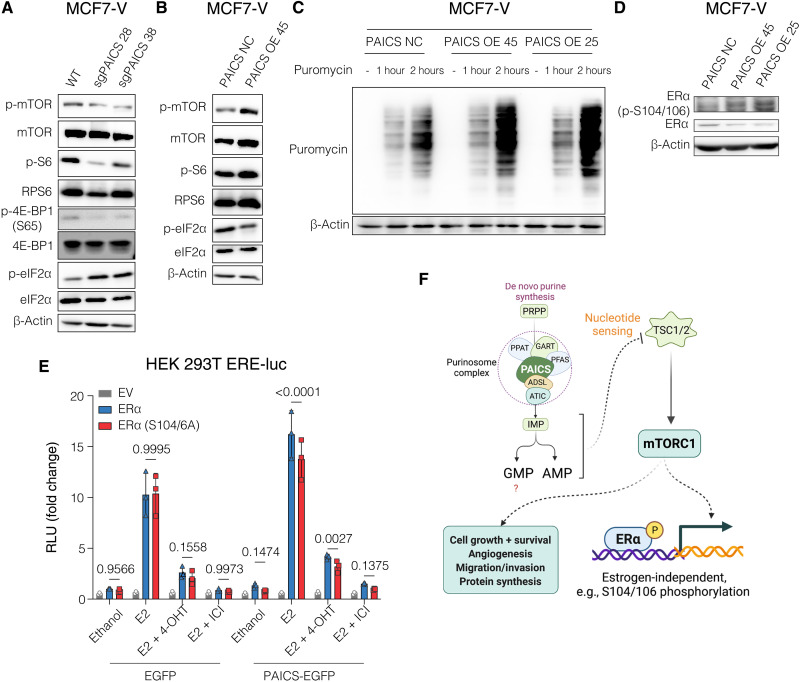
Expression of PAICS is correlated with mTOR activity and the global rate of protein translation. (**A** and **B**) Immunoblot of the indicated proteins. (**C**) Immunoblot of puromycin-labeled nascent polypeptides in total cell lysates. Cells were treated without or with puromycin (1 μM) for 1 and 2 hours. (**D**) Immunoblot of phosphorylated S104/106 of ERα and total ERα. (**E**) Luciferase assays in HEK 293 T cells using the ERE-Luc reporter, as in Fig. 7C. Data are represented as means ± SD of at least three independent experiments. Values for EV and ERα are the same as those in Fig. 7C as both S305A and S104/6A were tested in parallel. The statistical significance between the groups was analyzed by two-way ANOVA, and *P* values of <0.05 were considered statistically significant. (**F**) Schematic illustration summarizing the influence of PAICS levels on mTOR signaling. The scheme was created with Biorender.com.

Nucleotide sensing stimulates mTOR signaling through the regulation of the tuberous sclerosis complex (TSC) complex and the guanosine triphosphate (GTP)–binding protein Rheb (*45*). We therefore expected that PAICS protein levels might be correlated with mTOR activity. We monitored mTOR activity by looking at the phosphorylation of mTOR on S2448 and of S6; these can serve as proxies of mTOR activation because both are phosphorylated by S6 kinase. In the case of mTOR, phosphorylation by S6 kinase is part of a feedback loop (*46*). By immunoblotting, we found that PAICS deficiency correlates with decreased phosphorylation of mTOR at S2448 and of S6 and increased phosphorylation of the translation initiation factor eIF2α (Fig. 8A). Reduced and increased phosphorylation of S6 and eIF2α, respectively, leads to reduced translation. Conversely, the overexpression of PAICS is correlated with an increase in mTOR activity, S6 phosphorylation, and decreased phosphorylation of eIF2α (Fig. 8B). When PAICS was overexpressed in noncancerous RPE1 cells, we also detected increased phosphorylation of mTOR (fig. S9A). To confirm that the reduced activity of mTOR in PAICS-deficient cells is due to a depletion of purine nucleosides, we examined the phosphorylation of mTOR and its substrate 4E-BP1 upon supplementing the medium with the nucleosides adenosine, guanosine, and inosine. We found that adding nucleosides reverted the reduction of mTOR and 4E-BP1 phosphorylation in PAICS-deficient cells (fig. S9B). Phosphorylation of 4E-BPs, including 4E-BP1, prevents the sequestration of the initiation factor eIF4E, thereby promoting translation. Increased expression, availability, and phosphorylation of eIF4E and hence global translation were reported to be associated with tamoxifen resistance (*47*, *48*). Differential regulation of mTOR activity and eIF2α implies a direct correlation between PAICS and the global protein translation machinery. To test this, PAICS-overexpressing cells were treated with puromycin for specific time points and tested for the incorporation of puromycin into nascent polypeptides by immunoblotting with an anti-puromycin antibody. We observed more puromycin incorporation when PAICS was overexpressed and hence increased translation (Fig. 8C). Therefore, PAICS levels are directly correlated with mTOR activation and global translation.

Activation of Akt/PI3K/mTOR can promote the activation of ERα by estrogen and tamoxifen and in a ligand-independent manner (*49*). In the presence of estrogen, mTOR was previously shown to boost ERα activity through the phosphorylation of S104 and S106, which are located in AF-1 (*50*). S104/106 was shown to be phosphorylated in a ligand-independent manner by MAPK in vitro and in vivo, and their phosphorylation is required for the agonistic effects of tamoxifen (*51*). Therefore, we expected that PAICS expression might influence ERα activity through mTOR-stimulated phosphorylation of S104/106. We observed an increased phosphorylation at S104/106 when PAICS was overexpressed and, again, most obviously when one considers the reduced levels of total ERα (Fig. 8D and fig. S7B). In addition, using an ERE-luciferase reporter assay with HEK 293 T cells, we found a significant impairment of the transcriptional activity of the ERα double-mutant S104/106A upon PAICS overexpression, which implies that the activity of ERα is shifted to be more dependent on these sites (Fig. 8E). Together, we conclude that PAICS expression levels are directly correlated with mTOR activity, global translation, and phosphorylation of ERα at S104/106, which can all contribute to tamoxifen resistance.

### Pharmacological inhibition of PAICS sensitizes ERα^+^ breast cancer cells to 4-OHT

**Fig. 9.**
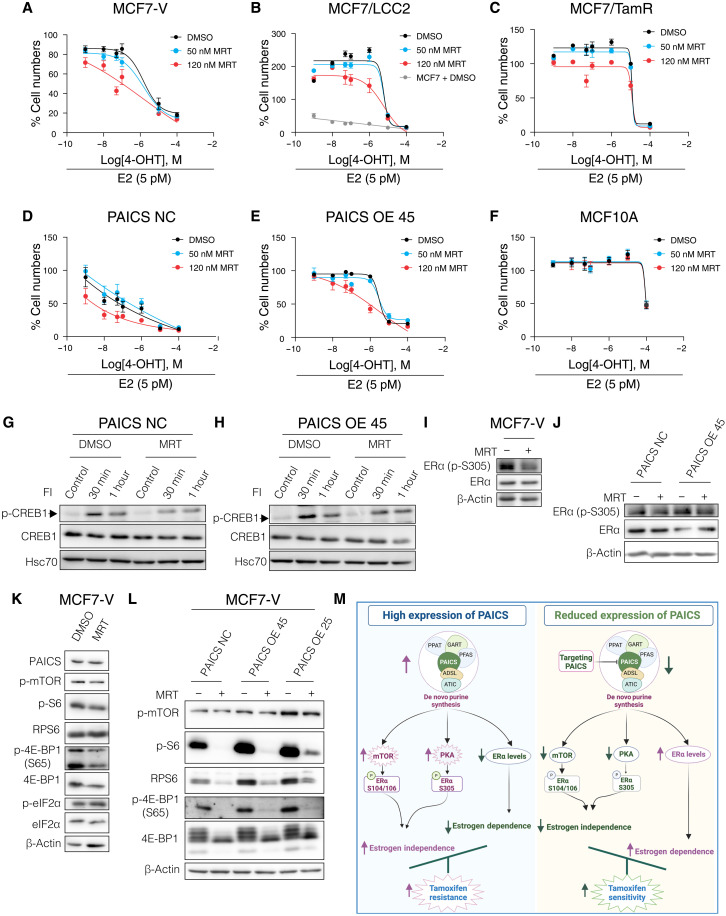
Pharmacological inhibition of PAICS sensitizes ERα^+^breast cancer cells to 4-OHT. (**A** to **F**) Dose-response curves with increasing doses of 4-OHT in the presence of either vehicle (DMSO) or MRT (50 nM or 120 nM), with cell densities measured with a crystal violet assay. For each curve, the result for a control group treated with vehicle was set to 100%. Data are represented as mean ± SD of at least three independent experiments. For (B), the dose-response curve obtained with MCF7 cells, the parent cell line of MCF7/LCC2, is included for comparison. (**G** and **H**) Immunoblots of phosphorylated (S133) and total CREB1. Cells were pretreated with either vehicle (DMSO) or MRT (500 nM) for 24 hours. Then, they were further treated with FI for 30 min and 1 hour. Simultaneously, untreated cells (control) were used as a control that corresponds to basal levels of CREB1 phosphorylation. Hsc70 was used as an internal control. (**I** and **J**) Immunoblots of phosphorylated (S305) and total ERα. Cells were treated with either DMSO or MRT (500 nM) for 24 hours. (**K** and **L**) Immunoblots of the indicated proteins in total cell lysates of cells treated with either vehicle (DMSO) or MRT (500 nM) for 24 hours. (**M**) Schematic illustration summarizing the impact of alterations of PAICS expression levels on cell signaling and ERα activity. The scheme was created with Biorender.com.

Because PAICS levels can be up-regulated in cancer (Fig. 2, A to C, and fig. S2, A to D), we sought to evaluate PAICS as a potential drug target and to test the efficacy and the safety profiles of the small-molecule PAICS inhibitor MRT00252040 (MRT) (*45*, *52*). We first generated dose-response curves of MRT for the ERα^+^ breast cancer cell lines MCF7, T47D, and MCF7-V; the tamoxifen-resistant cell lines MCF7/LCC2 and MCF7/TamR (*53*, *54*); and the noncancerous breast epithelial cell line MCF10A (fig. S10A). Calculations of approximate IC_50_ values showed that MRT is slightly more potent in the tamoxifen-resistant cell lines MCF7/LCC2 and MCF7/TamR by about 4- and 1.7-fold, respectively, by comparison with the tamoxifen-sensitive WT MCF7 cells (fig. S10B). MCF10A showed a much lower sensitivity than the cancerous cells. The IC_50_ values of MCF7 (about 460 nM) and MCF10A (about 110 μM) cells indicate a large therapeutic window of up to 240:1 (fig. S10B). To determine whether MRT treatment would result in an increased 4-OHT sensitivity of ERα^+^ breast cancer cells, two doses of MRT corresponding to ~IC_10_ (50 nM) and ~ IC_20_ (120 nM) (fig. S10C) were combined with increasing doses of 4-OHT with our panel of cell lines (Fig. 9, A to F, and fig. S10, D and E). At these low doses, MRT increased the sensitivity of the tamoxifen-tolerant MCF7-V or tamoxifen-resistant cell lines MCF7/LCC2 and MCF7/TamR, and PAICS OE 45 cells to 4-OHT (Fig. 9, A to E, and fig. S10, D and E). Moreover, the drug combination did not result in a significant difference compared to 4-OHT alone in the noncancerous cell line MCF10A (Fig. 9F). To further confirm the safety of the drug combination, two additional (nonbreast) noncancerous cell lines (RPE1 and BEAS-2B) were tested; again, the combinations did not show any additional toxicities, as compared to the treatment with 4-OHT alone (fig. S10, F and G). To test the effect of MRT on cell proliferation, MCF7-V cells were cotreated with or without MRT in the presence of E2, E2 + 4-OHT, and E2 + ICI. MRT treatment resulted in increased sensitivity to 4-OHT and dependency on E2 (fig. S10, H and I).

We speculated on the basis of our aforementioned results (Fig. 6 and fig. S8) that MRT might be an indirect inhibitor of cAMP/PKA signaling. We found that pretreatment with MRT resulted in reduced FI-induced CREB1 phosphorylation in PAICS-overexpressing MCF7-V cells (Fig. 9, G and H). MRT treatment also led to decreased phosphorylation of ERα at S305 (Fig. 9, I and J). Therefore, inhibition of PAICS with MRT causes a reduction of cAMP/PKA-induced phosphorylation of CREB1 and ERα S305.

Because we have also shown that PAICS levels correlate with mTOR activity and global protein synthesis (Fig. 8 and fig. S9), we analyzed the impact of MRT on those. We found that MRT treatment of WT MCF7-V and PAICS-overexpressing cells resulted in a reduction of the phosphorylation of mTOR and of its substrates S6 and 4E-BP1 (Fig. 9, K and L). MRT also promoted the inhibitory hyperphosphorylation of eIF2α (Fig. 9). Similarly, down-regulation of protein translation by MRT was observed when WT MCF7-V and PAICS-overexpressing cells were pretreated with MRT and labeled with puromycin (fig. S10, J and K), in line with our previous findings (Fig. 8C). Therefore, the PAICS inhibitor MRT causes a reduction of mTOR activity and reduces the activity of the protein translation machinery.

## DISCUSSION

Our work casts a new light on the role of the de novo biosynthesis of purines in endocrine therapy resistance of breast cancer. With a genome-wide CRISPR-Cas9 screen, we identified a set of genes that potentially contains new therapeutic targets and/or biomarkers of endocrine therapy responses (Fig. 1, B and C). One of the top hits encodes the enzyme PAICS whose role in breast cancer was largely unknown. Here, we report the discovery of an important correlation of PAICS levels with tumor progression, estrogen-independent cell proliferation, and tamoxifen resistance of ERα^+^ breast cancer. Our findings also highlight mechanistic connections between PAICS and the cAMP/PKA and mTOR signaling pathways. From a therapeutic perspective, we propose that PAICS may be a promising drug target to overcome tamoxifen resistance of ERα^+^ breast cancer.

Metabolic enzymes are commonly deregulated in cancer due to the increased demand caused by rapid cell proliferation (*55*, *56*). The biosynthesis of purines occurs through the purine salvage or de novo biosynthesis pathways. The salvage pathway is predominant and involves the recycling of nucleotide metabolites to regenerate purines. The de novo purine biosynthesis is the biological process that converts phosphoribosyl pyrophosphate to inosine monophosphate (IMP) and from there to AMP and guanosine monophosphate (GMP). This process is energy-intensive and relies on 10 highly conserved steps catalyzed by six different enzymes. Increased cellular demand of purines stimulates these enzymes to form a multicomponent, dynamic, non–membrane-bound complex known as the purinosome complex (*57*). PAICS catalyzes steps 6 and 7 of de novo purine biosynthesis (*58*). High PAICS expression levels are correlated with the formation of multiple tumors, including breast (*59*), bladder (*60*), prostate (*61*), gastric (*62*), colorectal (*63*), and lung (*64*) carcinomas and acute myeloid leukemia (*52*). Hypomethylation of the *PAICS* gene was reported for lung cancer cells as compared to normal lung cells (*64*), possibly contributing to elevated expression. By comparing the expression levels of PAICS in cancerous and noncancerous cell lines, we observed consistently higher protein levels of PAICS in the cancer setting (fig. S2, A to D). Our analysis of PAICS protein expression using a TMA of human clinical breast biopsies showed a significantly higher expression of PAICS in malignant tissue cores in both ERα^+^ and ERα^−^ breast samples (Fig. 2, A to C). This could be explained by an increased dependence of cancer cells on the synthesis of the nucleoside triphosphate precursors AMP and GMP to maintain elevated rates of transcription, DNA replication, cell division, and cell signaling (*64*). Further analysis of the TMA data revealed a significant correlation of PAICS expression and breast tumor proliferation, invasiveness, grade, and TNM stage in ERα^+^ samples rather than ERα^−^ ones (Fig. 2, A to G, and fig. S2, E to J). In line with this, survival analyses of patients with ERα^+^ and ERα^−^ breast cancer revealed a statistically significant correlation between the expression levels of *PAICS* and poor outcome in ERα^+^ breast cancer including those that were treated with tamoxifen. In contrast, there was no significant correlation for ERα^−^ breast cancer (Fig. 2, H to J, and figs. S2K and S3A). This partially agrees with previous reports about *PAICS* being a highly prognostic gene for breast cancer growth and aggressiveness, irrespective of ERα status (*65*), and about high mRNA levels of *ATIC*, encoding another enzyme of the pathway being associated with poor survival of patients with breast cancer (*66*). To our knowledge, a role of PAICS in ERα signaling and regulation had not been established before.

### Role of PAICS in cell migration, proliferation, and the regulation of ERα

The finding that PAICS is up-regulated in tumors suggests that it might be a favorable therapeutic target. Our data show that a deficiency of PAICS results in an enhanced sensitivity of ERα^+^ breast cancer cells to 4-OHT, while its overexpression leads to a resistant phenotype (Fig. 3, K and L, and fig. S6, A to D). High expression of PAICS is associated with cell proliferation, migration, and invasion (*60*, *61*, *63*). We show that the overexpression of PAICS in the noncancerous cell line RPE1 is associated with increased cell migration (fig. S5, A to D). Moreover, in ERα^+^ breast cancer cells, we found that PAICS enhances cell migration and modulates cell proliferation toward estrogen-independence (Fig. 3, C to J, and fig. S5, F to K). The observed increased cell migration is consistent with an in vivo animal study showing that PAICS depletion could inhibit primary and metastatic breast tumor growth (*65*). In the same study, PAICS depletion led to a more pronounced reduction in cell proliferation in vivo than in vitro. In an analysis of a public microarray dataset, three other enzymes of the de novo purine biosynthesis pathway (GART, PFAS, and ATIC) were pinpointed as critical hubs, which were highly expressed in tamoxifen-resistant MCF7 cells; curiously, their knockdown did not affect tamoxifen sensitivity (*66*). In our study, differential effects of 4-OHT and ICI are notable in the cell proliferation profiles of ERα^+^ breast cancer cells that overexpress PAICS (Fig. 3, E to G, and fig. S5, I to K). Enhancement of cell proliferation in the absence of estrogen and in the presence of 4-OHT would imply either an up-regulation in signaling pathways that promote cell growth and proliferation in general or an altered mode of activation of ERα. Estrogen-independent or 4-OHT–resistant or even 4-OHT–promoted activation of ERα might occur through AF-1 or through the effects of phosphorylation of S305 in the hinge domain, although a contribution of AF-2 cannot be excluded (*4*, *10*, *15*–*17*, *44*, *50*, *51*, *67*). The inhibition of the PAICS-stimulated proliferation by ICI demonstrates that the cells are still dependent on ERα. In contrast, the depletion of PAICS sensitized the cells to both 4-OHT and ICI and restricted the proliferation of cells to the presence of estrogens. These findings suggest that PAICS expression affects the regulation of ERα activity and the response of ERα to different ligands.

Expression of ERα is an essential determinant of the tamoxifen response. We demonstrated that there is an inverse correlation between PAICS levels and the expression levels of ERα mRNA and protein (Fig. 4, A to G, and fig. S7, A to C). Independent of the levels of PAICS, we found that overexpression of ERα is sufficient to enhance the sensitivity of breast cancer cells to 4-OHT treatment (fig. S7, I to K). Together, these results suggest that PAICS levels affect the expression of *ESR1* (ERα) at the transcriptional level and that this contributes to the modulated response to 4-OHT. This is consistent with previous studies showing that low levels of ERα expression can lead to tamoxifen resistance (*47*, *68*, *69*). ERα expression can be regulated by several transcription factors, DNA methylation, histone modifications, RNA-binding proteins, and microRNAs (*47*). The exact mechanisms of how PAICS or purines regulate *ESR1* expression remain to be elucidated. It is conceivable that this could be due to the PAICS-associated regulation of mTOR and the global translation rate. It was previously shown that multiple mRNA transcript variants of ERα contain upstream open reading frames (uORFs) in their 5′ untranslated regions (*70*). These uORFs primarily have a negative effect on the translation of the downstream ORF encoding ERα. However, upon cellular stress and down-regulation of global translation, leaky scanning of these mRNAs can bypass uORFs and lead to translation reinitiation and hence rescue of translation of the long ORF (*70*, *71*). An interplay between ERα and de novo purine biosynthesis is also supported by a previous report showing that ERα signaling induces a metabolic switch in breast cancer cells, including increased biosynthesis of purines (*72*). In the same study, *PAICS* and *PPAT*, which encodes another enzyme of the de novo purine biosynthesis pathway, were identified as ERα target genes. The down-regulation of the transcription of *ESR1* at higher levels of PAICS might be due to a regulatory, perhaps even a negative feedback role of purines on the transcription of *ESR1*, or due to hypothetical purinosome-independent functions of PAICS. A previous report has shown that the availability of purines and purine nucleotides can regulate the transcription of a broad class of genes that are lacking specific consensus sequences proximal to their promoters (*73*). It remains to be seen whether *ESR1* falls into this category.

### PAICS levels determine cAMP/PKA activation and ERα phosphorylation at S305

Apart from the crucial necessity of purines for the generation of DNA and RNA molecules, purine nucleotides such as ATP and GTP are indispensable for providing cellular energy and for signaling (*74*). Depending on the energy needs, macromolecules such as carbohydrates, proteins, and fatty acids can be used by mitochondrial and glycolytic pathways to fuel the cells with ATP. This requires a sufficient supply of its cytosolic precursor adenosine diphosphate (ADP). ADP, in turn, results from the phosphorylation of AMP, which is synthesized from IMP through a two-step enzymatic reaction. Our results revealed that PAICS expression levels can affect the cellular pool of ATP (Fig. 6, A and B, and fig. S8A). This might result from increased synthesis of its precursor IMP by the de novo purine biosynthetic pathway. This possibility ties well with a recent study showing a reduction in the levels of AMP, ADP, and ATP upon using chemical inhibitors of purine biosynthesis (*45*). Another speculation would be that PAICS or the purinosome might have a role in the regulation of mitochondrial function. An interplay between mitochondria and the purinosome is very plausible because recent studies using superresolution microscopy revealed their colocalization (*75*, *76*). Moreover, the multistep process of the de novo biosynthesis of purines requires a coordinated supply of energy, substrates, amino acids, and cofactors from mitochondria (*75*, *76*).

The levels of ATP and the capacity of its synthesis might affect directly or indirectly multiple cellular signaling pathways (*77*). Upon activation of the enzyme adenylate cyclase, ATP is converted to the second messenger cAMP. cAMP-activated PKA can drive tumor formation in multiple endocrine tissues, including breast (*78*–*80*). An inactivating mutation of the *PRKAR1A* gene, which encodes the regulatory subunit R1α of PKA, was shown to increase PKA activity and to promote carcinogenesis in mammary tissues (*78*). Down-regulation of RIα and PKA overexpression were shown to be associated with increased phosphorylation of S305 of ERα and tamoxifen resistance in breast cancer (*16*). Clinical reports showed that phosphorylation of S305 of ERα was associated with resistance to tamoxifen adjuvant therapy in premenopausal women (*16*, *81*, *82*). Previous work from our laboratory had shown that, in response to cAMP, the phosphorylation of the coactivator-associated arginine methyltransferase 1 at S448 by PKA is indispensable for the activation of unliganded ERα, albeit insufficient on its own (*83*). Later on, other ERα interactors were discovered to contribute to the activation of ERα in response to cAMP, notably LSD1, Co-REST, HDAC1/6, Hsp90 (*84*), and, more recently, CREB1 (*85*). Here, we showed that PAICS levels were directly correlated with FI-induced cAMP levels, PKA activity, and the phosphorylation of PKA substrates, such as S133 of CREB1 and S305 of ERα (Figs. 6, C to F, and 7, A to D; and figs. S7B and S8, B to E). Regulation of ERα activity by PKA is specific to tissue, cell type, and target gene. Although PKA promotes ligand-independent activation of ERα, it was also known to regulate ERα activity in the presence of estrogen (*86*–*88*) and notably to decrease the estrogen dependence of ERα activation for specific genes in MCF7, T47D, and MCF7/LCC2 cells (*11*). Moreover, it was reported to cause a switch of the behavior of tamoxifen from antagonist to agonist due to an arrest of ERα in an active conformation that is able to recruit coactivators. This was found to convert tamoxifen to a growth stimulator rather than an inhibitor when S305 of ERα is phosphorylated (*16*, *67*). Therefore, we propose that the impact of cAMP/PKA signaling on ERα activity explains how PAICS levels can be linked to estrogen-independent and 4-OHT–resistant proliferation of ERα^+^ breast cancer cells.

### PAICS correlates with mTOR activity and ERα phosphorylation at S104/106

We also explored the role of the PI3K/Akt/mTOR signaling pathway, which is known to regulate cell survival, proliferation, and metabolic homeostasis (*20*). PI3K represents the most commonly altered pathway in breast cancer and a critical hub in hormone independence and endocrine therapy resistance of ERα^+^ breast cancer (*49*, *89*). Activation of PI3K has been shown to be associated with poor recurrence-free survival of patients with breast cancer after tamoxifen adjuvant therapy (*89*). A cross-talk between PI3K/Akt/mTOR and ERα can lead to increased ERα transcriptional activity both in the absence of ligand and in the presence of estrogen or tamoxifen (*90*, *91*). Moreover, PI3K/Akt/mTOR can induce the phosphorylation of c-Jun that is part of the AP-1 complex that contributes to ERα activity, breast cancer progression, invasiveness, and tamoxifen resistance (*92*, *93*). Activation of PI3K/Akt/mTOR has been suggested to be required for the adaptation of ERα^+^ breast cancer to hormone deprivation (*89*). Targeting mTOR with small-molecule inhibitors, such as everolimus or rapamycin, was shown to reverse hormone-independent cell growth and endocrine resistance (*89*, *94*–*96*). Data from our screens as well as those of others (*32*) highlight *TSC1* and *TSC2* as genes whose depletion boosts cell proliferation in the absence of estrogen and in the presence of 4-OHT or ICI (Fig. 1B). Our results show a strong correlation of PAICS levels with the activation of mTOR signaling and the global protein translation machinery (Fig. 8, A to C, and fig. S9A). This is supported by a recent report identifying purine nucleotides, but not pyrimidines, as an upstream regulatory stimulus for the activation of the mTOR complex mTORC1 (*45*). mTORC1 acutely senses alterations in the levels of adenine nucleotides by TSC and not through AMP-activated protein kinase or Rag GTPases. mTORC1 could also sense long-term changes in guanine nucleotide levels. Therefore, we speculate that the observed correlation of PAICS with mTOR activity could be explained by alterations of the levels of purine nucleotides (*45*). In agreement with this idea, our data show that supplementation with purine nucleosides rescues the reduced mTOR activity resulting from the knockdown of PAICS (fig. S9B).

Phosphorylation of ERα within the AF-1 domain stimulates ERα ligand-independent activity and contributes to endocrine therapy resistance (*51*). Akt and ribosomal S6 kinase 1, which is a downstream target of mTOR, were found to promote ERα transcriptional activity by phosphorylating S167 in response to growth factors (*90*, *91*). This phosphorylation is positively correlated with endocrine therapy response and better survival of patients with primary breast tumors (*69*). In response to estrogen, the mTORC1 component raptor binds to and translocates with ERα to the nucleus. This allows the interaction of mTOR with ERα and phosphorylation of S104/106, also located within AF-1 (*50*). ERα was found to be phosphorylated at S104/106 by MAPK both in vitro and in vivo (*51*) and by cyclin A-CDK2 independently of ligand (*97*). Phosphorylation at these sites is required for the agonistic activity of tamoxifen and may have a role in tamoxifen resistance (*51*). Our results indicate that the activity of ERα becomes more dependent on the phosphorylation of S104/106 when PAICS is overexpressed (Fig. 8, D and E, and fig. S7B). This shift might explain the estrogen-independent and tamoxifen-resistant cell proliferation observed in these cell lines. The phosphorylation of S104/106, when PAICS is overexpressed, might be mediated by a hyperactive mTOR, MAPK, or yet another kinase. Which kinase(s) is most relevant under these circumstances remains to be investigated.

### Pharmacological significance

Mechanistically, the small-molecule PAICS inhibitor MRT has been shown to inhibit the SAICAR synthetase activity of PAICS because its binding to PAICS resulted in accumulation of metabolites upstream of SAICAR (*52*). It has been reported for leukemia that targeting PAICS using MRT reduces cell proliferation by inhibiting DNA synthesis and by promoting apoptosis in vitro and in vivo (*52*). Moreover, PAICS knockdown causes a reduction in tumor growth (*61*–*63*) and metastasis (*63*) in mouse xenograft models. Apart from the metabolic role of PAICS, its knockdown was found to induce DNA damage and to impair DNA damage repair by reducing HDAC1/2 deacetylase activity and to enhance the sensitivity to DNA damaging agents like cisplatin in gastric carcinoma in vitro and in vivo (*62*). In breast cancer, targeting PAICS was shown to reduce the proliferation of breast cancer cells by inducing a cell cycle arrest (*59*). We have evaluated the therapeutic potential of MRT in ERα^+^ breast cancer cell lines. We demonstrate that MRT selectively affects breast cancer cell lines compared to nonmalignant cells (fig. S10, A to C). We found that low doses of MRT resensitize 4-OHT–tolerant and 4-OHT–resistant cell lines to 4-OHT with minimal toxicities on noncancerous cells (Fig. 9, A to F, and fig. S10, D to I). We therefore propose a low-dose combination of MRT + 4-OHT as a potentially nontoxic drug combination to treat tamoxifen-resistant breast cancer. We have demonstrated that MRT treatment has the potential to impair signaling by cAMP/PKA and mTOR, to reduce global protein translation and phosphorylation of S305 of ERα (Fig. 9, G to L, and fig. S10, J and K). These results provide a mechanistic explanation for how MRT could resensitize cells to 4-OHT and contribute to overcoming tamoxifen resistance.

Collectively, our study shows that PAICS expression levels are directly correlated with tamoxifen resistance, which can be attributed to increased cAMP-induced PKA and purine-induced mTOR signaling. cAMP/PKA and mTOR signaling might result in the phosphorylation of ERα at S305 and S104/106, respectively. Modulation of the levels of ERα phosphorylation at these sites might tune the balance of ERα activity in response to estrogen and 4-OHT. Last, our results showed that targeting PAICS either with gene editing or with the specific small-molecule inhibitor MRT can sensitize breast cancer cells to 4-OHT (Fig. 9M).

### Limitations

The huge number of cells required for a CRISPR-Cas9 screen made it difficult to do the screen in parallel with additional cell lines. This would have helped to confirm and to diversify the list of genetic biomarkers and/or targets. Moreover, although the inclusion of a treatment with a cell-permeable form of cAMP in the screen seemed worthwhile, with our cutoff values, we could not identify significant hits specifically related to this particular treatment. Perhaps other ways of activating PKA continuously throughout the duration of the screen might be more appropriate. Although our findings strongly suggest a correlation between PAICS and ERα expression, more studies are needed to identify the underlying molecular mechanism. Furthermore, the connection between PAICS and endocrine resistance needs to be further strengthened in the future with cohort studies in patients with breast cancer. Last, the small-molecule PAICS inhibitor MRT is a recent investigational drug, and, therefore, future preclinical studies are necessary for its progression toward clinical trials.

## MATERIALS AND METHODS

### Cell lines

MCF7-V cells were a gift from W. Zwart (Netherlands Cancer Institute, Amsterdam, The Netherlands); profiling of the highly polymorphic short tandem repeat loci showed that they are 88% related to WT MCF7 cells; they are more tolerant to 4-OHT and display more robust ERα responses (*34*, *37*). In the following, for cell lines available from American Type Culture Collection (ATCC), ATCC references are given in parenthesis: MCF7 (HTB-22), BT474 (HTB-20), SKBR3 (HTB-30), MDA-MB-231 (HTB-26), HCC1937 (CRL-2336), MCF10A (CRL-10317), HEK 293 T cells (CRL-3216), human skin fibroblasts CCD-1079Sk (CRL-2097), RPE1 cells (CRL-4000), BEAS-2B (CRL-9609), and BZR (CRL-9483). T47D cells were purchased from Sigma-Aldrich (no. 85102201). MCF7/LCC2 (*53*) and MCF7/TamR (*54*) (a gift from D. Barrow, Cardiff University) were used as tamoxifen-resistant cell lines. RPE1 Ras and the human skin fibroblast derivatives hTERT, hTERT LT, and hTERT LT Ras, generated as described before (*38*), were a gift from V. Xavier and J.-C. Martinou (University of Geneva, Switzerland) (*39*). Human breast carcinoma H3396 cells (*98*) were a gift from H. Gronemeyer’s laboratory (IGBMC, Strasbourg).

### Plasmids

The human Brunello CRISPR knockout pooled library (*36*) was a gift from D. Root and J. Doench (Addgene, no. 73179). The vector lentiCRISPRv2 (*99*) (Addgene, no. 52961) for expression of Cas9 was a gift from F. Zhang (Addgene, no. 52961). The VSV-G envelope expressing plasmid pMD2.G (Addgene, no. 12259) and the lentiviral packaging plasmid psPAX2 (Addgene, no. 12260) were gifts from D. Trono’s laboratory (École polytechnique fédérale de Lausanne) and were used to produce lentiviral particles for expression of Cas9 and sgRNAs. For the specific knockouts of *PAICS* and *RPE65*, the selected sgRNA sequences target the third exon of *PAICS* and the fifth exon of *RPE65* (data S5). Oligonucleotides were purchased from Microsynth (data S5), annealed, and cloned into the Bbs I site of the vector lentiCRISPRv2 for expressing sgPAICS and sgRPE65. For transient transfection of sgPAICS and sgRPE65, annealed oligos were cloned into the Cas9-expressing vector pSpCas9(BB)-2A-Puro (PX459) V2.0 (Addgene, no. 62988), which was a gift from F. Zhang. The plasmid pPAICS-EGFP was a gift from S. Benkovic (Addgene, no. 99108) and was used for the transient and stable overexpression of a PAICS-EGFP fusion protein. As a negative control for the transient overexpression of PAICS, EGFP expression plasmid pEGFP-C1 (Clontech, no. 6084-1) was used. For the generation of ERα-overexpressing clones, the plasmid F-ER (*100*) was used. For transient expression of WT and mutant ERα in HEK 293 T cells, plasmid HEG0 (*101*) and its derivatives HEG0 S305A and HEG0 S104/106A were used, with their expression vector pSG5 (*102*) as the empty vector control. For luciferase assays, the following reporter plasmids were used: XETL (here, referred to as ERE-Luc) (*103*), CRE-Luc (Stratagene), and the Renilla luciferase transfection control reporter pRL-CMV (Promega, no. E2261). To visualize PKA activity, plasmid pcDNA3-PKA-SPARK was used. It was a gift from X. Shu (Addgene, no. 106920).

### Cell culture

MCF7/LCC2 and MCF7/TamR cells were cultured in Dulbecco’s modified Eagle’s medium (DMEM) without phenol red (Thermo Fisher Scientific, no. 11880036), supplemented with 5% charcoal-treated fetal bovine serum (CHFBS), penicillin/streptomycin (100 μg/ml; Thermo Fisher Scientific, no. 15070063), 4 mM l-glutamine (Thermo Fisher Scientific, no. 25030081), and 100 nM 4-OHT (Sigma-Aldrich, no. H7904). MCF10A were cultured in DMEM/F12 medium (Thermo Fisher Scientific, no. 31331028) containing 5% horse serum (BioConcept, no. 2-05F00-I), penicillin/streptomycin (100 μg/ml), human epidermal growth factor (10 ng/ml; Sigma-Aldrich, no. E9644), insulin (5 μg/ml; Sigma-Aldrich, no. I9278), and 1 μM dexamethasone (Sigma-Aldrich, no. D8893). All other cell lines were cultured in DMEM/GlutaMAX medium (Thermo Fisher Scientific, no. 31966047), supplemented with 10% FBS (PAN-Biotech, no. P40-37500) and penicillin/streptomycin (100 μg/ml). Before treatments, hormone deprivation was done by maintaining the cells in DMEM medium without phenol red, containing 5% CHFBS, penicillin/streptomycin, and l-glutamine for 72 hours. All cell lines were maintained at 37°C with 5% CO_2_ in a humidified incubator.

### Genome-wide CRISPR-Cas9 screen

#### Library amplification and validation of its complexity

The protocol was adapted from the Broad Institute protocol of amplification of library plasmid DNA available from the GPP web portal (https://portals.broadinstitute.org/gpp/public/resources/protocols). Briefly, in a total of four cuvettes, 400 ng of plasmid library DNA was transformed into 100 μl of Endura electrocompetent cells (Lucigen, no. 60242-2) by electroporation with a MicroPulser (Bio-Rad, no. 1652100) using the Ec1 setting (1.8 kV). Within 10 s of the pulse, 975 μl of recovery medium was added to each cuvette containing 25 μl of the transformation mixture and then transferred to a loosely capped tube containing an additional 1 ml of recovery medium. Tubes were incubated with shaking at 250 rpm for 1 hour at 37°C. The four tubes were pooled and mixed well. Bacteria were then spread on five ampicillin agar dishes of 500 cm^2^, in addition to one control dish without ampicillin, and incubated overnight at 37°C. Then, bacterial colonies were scraped into cold LB medium and centrifuged, and pellets were weighed (total weight about 1 to 2 g). Plasmid DNA was purified by midi-prep using a NucleoBond Xtra Midi kit (Machery Nagel, no. 740410.50) according to the manufacturer’s protocol. To check library representation, purified DNA was amplified by polymerase chain reaction (PCR) using GoTaq DNA polymerase (Promega, no. M3001) as follows: Four parallel PCR reactions were prepared, each containing 200 ng of DNA and the universal primers P5 and P7 (table S1) used for Illumina sequencing (Microsynth) (*36*). The PCR program was as follows: 95°C for 1 min, 95°C for 30 s for DNA denaturation, primer annealing at 53°C for 30 s, and extension at 72°C for 30 s, for 28 cycles, and then 72°C for 10 min for the final extension step. PCR amplicons were further purified using the GenElute PCR Clean-Up Kit (Sigma-Aldrich, no. NA1020) and checked for correct sizes by agarose gel electrophoresis. Quantity was determined using a NanoDrop (Thermo Fisher Scientific). The four PCR reactions were pooled into one sample and sequenced by NGS at the iGE3 Genomics Platform of the University of Geneva using a HiSeq 4000 instrument (Illumina). Approximately 24 million single-end reads of 50 base pairs (bp) that contain the sgRNA sequences were obtained and checked for quality using the FastQC tool available within the Galaxy platform (https://usegalaxy.org/). Sequencing adaptors were trimmed using the cutadapt tool, and the sgRNA sequences were aligned to the Brunello library index using the Bowtie tool. The amplified library contained about 90% perfectly matching sgRNAs, 0.01% of the sgRNAs were undetected, and the distribution skew ratio was 0.84 (*104*).

#### Preparation of lentiviral particles

The protocol was adapted from a previously described one (*104*). HEK 293 T cells were seeded into eight 15-cm dishes at a density of 30 million cells per dish. The next day, each dish received a cotransfection mixture of 3.77 μg of pMD2.G, 7.55 μg of psPAX2, and 15.11 μg of pooled library plasmid DNA in serum-free DMEM medium using PEI MAX 40K (Polysciences Inc., no. 24765-100) at a DNA:PEI MAX ratio of 1:8.2. Media were changed after 12 to 14 hours, and lentivirus-containing supernatants were collected after 48, 72, and 96 hours after transfection. Supernatants were spun at 1000*g* for 5 min, then filtered through 0.45-μm sterile filters, and complemented with sterile 40% poly(ethylene glycol) (PEG) 8000 in 400 mM NaCl (PEG 8000 from Sigma-Aldrich, no. 25322-68-3) at a PEG:supernatant volume ratio of 1:3. Tubes were incubated at 4°C on a rotating wheel for 1 to 2 hours and then centrifuged at 4000*g*, at 4°C for 30 min. The pellets containing the lentiviral particles were resuspended in fresh culture medium in 1/20th of the initial collection volume. The first two concentrated lentiviral suspensions were frozen at −80°C and only thawed on ice on the day of the third collection to be pooled into one lentiviral preparation.

#### Viral transduction and puromycin selection

To achieve >500× coverage of the library plasmids for each treatment group, a total of 110 dishes (15 cm) were seeded with MCF7-V cells at a density of 10 million cells per dish. The pooled lentiviral suspension (120 μl) was added per dish with polybrene (8 μg/ml; Sigma-Aldrich, no. 107689-10G) to enhance the transduction efficiency. The volume of concentrated viral suspension was optimized for 50% transduction efficiency in MCF7-V cells. Twenty-four hours after lentiviral transduction, cells were treated with puromycin (3 μg/ml; Cayman Chemical, no. 13884) for 7 days. A control dish of nontransduced MCF7-V cells was used as a positive control for the puromycin selection efficiency.

#### Drug treatments

Following the selection of lentivirally transduced cells with puromycin, cells were trypsinized, and cell pellets were resuspended in fresh culture medium and then pooled into one suspension of cells. A total of 100 million cells were collected and aliquoted into 20 tubes of 5 million cells each and centrifuged; cell pellets were washed with phosphate-buffered saline (PBS) and then kept at −20°C. These cells represented the transduced control cells before drug treatment (control T0). The rest of the cell suspension was allocated into drug treatment groups, each with a total of 100 million cells. Hence, each group was represented by 10 dishes (15 cm) seeded with 10 million cells per dish. After 24 hours, drug treatments were started, and medium with drugs was changed twice per week for a total period of 4 weeks. Treatment groups were as follows: vehicle control, E2 (100 pM), db-cAMP (10 μM), E2 (100 pM) + 4-OHT (100 nM), E2 (100 pM) + ICI (100 nM), E2 (100 pM) + 4-OHT (100 nM) + db-cAMP (10 μM), and E2 (100 pM) + ICI (100 nM) + db-cAMP (10 μM). Cells were passaged (one into two plates) if any of the groups reached about 90% confluency. After treating the cells for 4 weeks, cells were harvested, and, for each group, cell pellets were resuspended and pooled into one suspension of cells. Cell suspensions were aliquoted, and pellets were harvested as done for control T0.

#### Genomic DNA extraction, PCR, and NGS

The following was adapted from published protocols (*105*). Cells were lysed in digestion buffer [100 mM NaCl, 10 mM tris-HCl (pH 8), 25 mM EDTA, 0.5% SDS, and proteinase K (0.1 mg/ml)] and incubated with shaking at 50°C for 14 hours. Then, ribonuclease (1 μg/ml) was added to the lysates and incubated at 37°C for 1 hour. One volume of phenol/chloroform/isoamyl alcohol was added to the lysate and mixed well, and samples were centrifuged at 10,000*g* for 10 min. The aqueous phases at the top were transferred to new tubes, 5 M NaCl was added at 1/8 volume, and then 100% ethanol was added (twice as much as the volume of the original aqueous phase). Genomic DNA was recovered by centrifugation at 12,000*g* for 20 min. Pellets were rinsed twice with 70% ethanol, centrifuged at 12,000*g* for 5 min, and then left to air-dry for 15 min at room temperature. Pellets were dissolved in Tris-EDTA buffer at 65°C overnight or until dissolved. Genomic DNA samples of each treatment group were pooled into one sample, mechanically homogenized, and sheared by passing through a syringe with a 25-gauge needle. The size distribution of the sheared DNA was assessed by agarose gel electrophoresis, and DNA concentration quantitated using a NanoDrop. For each group, four parallel PCR reactions were prepared, each containing 4 μg of genomic DNA, the universal Illumina primer P5, and a barcoded primer P7, which was unique for each group (table S1). PCR program setup and purification of the PCR products were the same as for the initial validation of the complexity of the library. PCR amplicons were checked for correct sizes by agarose gel electrophoresis, and DNA was quantitated using a NanoDrop. The four PCR reactions were pooled into one sample and sequenced by NGS with single-end reads of 50 bp with indices. Sequence quality was checked using the FastQC tool, sequencing adaptors were trimmed using the cutadapt tool, and the sgRNA sequences were aligned to the Brunello library index using the Bowtie tool.

#### Data analysis

The CRISPR-Cas9 screen was done with two biologically independent replicates. Read sequences of sgRNAs were mapped to their corresponding genes (data S1), and a “β-score” was calculated using the MAGeCK-VISPR platform (data S2) (*32*, *106*). β-Score is similar to “log fold change,” and it indicates the respective enrichment (β > 0) or depletion (β < 0) of sgRNAs of a specific gene when compared to the control T0. For all genes, β ≥ 0.3 or β ≤ −0.3 and false discovery rate (FDR) value of ≤0.1 or ≥−0.1 were considered significantly enriched or depleted, respectively. We considered about 600 genes as essential for survival because their sgRNA counts were depleted in all conditions and eliminated from the subsequent analysis (data S3). Comparisons between every two groups were performed using the same cutoff values to identify significant differences between the β-scores. The counts of the four different sgRNAs per gene were checked in the two replicates to eliminate β-scores that were biased by sgRNA outlier values. Last, 58 genes were identified as the top enriched or depleted genes, and β-scores were hierarchically clustered using R packages (Fig. 1B and data S4). Literature curation narrowed down the list to 40 genes (data S5) with unknown functions in ERα^+^ breast cancer or endocrine therapy responses.

### Secondary CRISPR-Cas9 knockout screen

For the validation of the effects of the knockout of the 40 genes identified with the primary screen, the knockout of each gene was done individually and subjected to the same treatment groups. One sgRNA sequence was selected from the Brunello library on the basis of the calculated off-target score (*36*) and the consistency of read counts between the two biological replicates. sgRNA sequences corresponding to scrambled, and *RPE65* gene sequences were used as controls. sgRNA oligonucleotides (data S5) were cloned into lentiCRISPRv2 as described above and previously (*99*). Preparation of lentiviruses and transduction of MCF7-V cells were done as mentioned previously for the primary screen. For the drug treatments, each knockout cell line was seeded at a density of 7000 cells per well of a 96-well plate and treated for 2 weeks with the same treatment groups as during the primary screen. Cell growth was assessed using crystal violet staining. Briefly, cells were washed with PBS, incubated with 4% formaldehyde in PBS for 20 min, washed again with PBS, and then incubated with 0.1% crystal violet solution in distilled water for 30 min. The wells were washed thoroughly with distilled water and air-dried overnight. Crystals were dissolved in glacial acetic acid, and the absorbance was measured at 595 nm with the Cytation 3 Imaging Reader (Agilent). The average % cell growth of the 40 gene knockout cell lines and the treatment groups were visualized as a heatmap using GraphPad Prism version 8.0.0 (Fig. 1C).

### Generation of sgRNA knockout clones of PAICS and RPE65

sgPAICS and sgRPE65 lentiviral particles were produced in HEK 293 T cells as described above. MCF7-V cells seeded at 60% confluency in 10-cm dishes were incubated for 48 hours with complete culture medium containing 200 μl of concentrated lentiviral suspensions per dish. In parallel, one 10-cm dish of MCF7-V was incubated without viral particles and used as a nontransduced control for the puromycin selection. Puromycin (3 μg/ml) was added to both transduced and nontransduced MCF7-V cells for 4 days or until all the nontransduced cells were dead. For the isolation of single PAICS knockout clones, cells that were resistant to puromycin were sorted into single cells and seeded into a 96-well plate at a density of one cell per well using a BD FACS Aria III Cell Sorter (BD Biosciences). After clonal expansion, single clones were screened for either a complete knockout or a significant knockdown of PAICS protein levels by immunoblotting with a PAICS antibody. Two clones, #28 and #38, which showed a significant decrease in the PAICS protein levels compared to WT MCF7-V cells, were selected for further investigation. To generate the negative control cell line (sgNT), sgRPE65-mediated knockout cells that were resistant to puromycin were pooled.

### Generation of stable PAICS and ERα overexpression clones

Cells were transfected with the expression vectors pPAICS-EGFP (MCF7-V, T47D, and RPE1) or F-ER (MCF7-V) using jetOPTIMUS (Polyplus, no. PPT117-07), according to the manufacturer’s protocol. After 48 hours, cells were maintained in G418 (1000 μg/ml; Sigma-Aldrich, no. A1720) for 2 to 3 weeks or until the nontransfected control cells died completely. Individual cellular colonies were picked using 200-μl pipette tips, expanded, and analyzed by immunoblotting for the PAICS-EGFP fusion protein or ERα. For MCF7-V cells, two PAICS-overexpressing clones, #25 and #45, were selected for further validation, and clone #43, which did not show expression of exogenous PAICS, was selected as a negative control (PAICS NC). For T47D cells, clones #2 and #8 were overexpressing PAICS-EGFP, and clone #3 was selected as a negative control. For RPE1 cells, the PAICS-overexpressing clones #7, #9, and #17 were selected for further assays. Cellular morphology was visualized using an inverted light microscope. We found ERα to be overexpressed in MCF7-V clones #17 and #24, but not in clone #4, which was used as negative control (ERα NC).

### Fluorescence immunohistochemistry of breast TMA

The human breast TMA (US Biomax Inc., no. BRF1503f) consists of 150 cores of 75 cases. It contains three cases of adjacent normal breast tissue, three cases of breast fibroadenoma, two cases of breast cystosarcoma phyllodes, seven cases of breast intraductal carcinoma, and 60 cases of breast invasive ductal carcinoma. All cases are represented in duplicate cores. Data including TNM and pathology grade, with immunohistochemistry results for the markers HER-2/ERα/PR/Ki-67, were provided by the manufacturer (data S6). The protocol was adapted from a previously published one (*34*). First, paraffinized tissue sections were warmed up for 30 min at 60°C in a dry oven. Then, sections were deparaffinized and rehydrated by sequential incubation in the following solutions: xylene (two washes, 5 min each), 100% ethanol (two washes, 5 min each), 95% ethanol (one wash for 5 min), 70% ethanol (one wash for 5 min), and double distilled H_2_O (one wash for 5 min). Unmasking was done by heat-induced epitope retrieval using 10 mM sodium citrate buffer (pH 6). Tissues were boiled in the citrate buffer in the microwave at 600 W for 3 min, then left to cool until the temperature of the buffer reached 50°C, then boiled again for another 3 min, and left to cool to room temperature. Tissues were permeabilized and blocked in 0.3% Triton X-100 and 1% bovine serum albumin (BSA) in PBS (1% BSA in PBST) for 20 min. PAICS primary antibody was diluted at 1:25 in 1% BSA in PBST buffer, added to the tissue sections, and incubated in a humidified chamber overnight at 4°C. After two washes with PBS, tissues were incubated for 1 hour at room temperature with the anti-rabbit secondary Cy3 red-fluorescent antibody diluted to 1:1000 in 1% BSA in PBST buffer. Sections were washed twice in PBS for 5 min each, and then nuclei were counterstained by incubation with 4′,6-diamidino-2-phenylindole dye [1:30,000 in PBS, from stock solution (1 mg/ml); Thermo Fisher Scientific, no. 62248] for 5 min. Sections were washed three times in PBS for 5 min each, then rinsed for 5 min in a solution of 10 mM CuSO_4_ and 50 mM NH_4_Cl to reduce autofluorescence, and then rinsed briefly in double-distilled H_2_O. The TMA slide was mounted using the fluorescence mounting medium Fluoromount (Sigma-Aldrich, no. F4680) and left to dry in a dark chamber. Imaging was done using the high-throughput slide scanner Zeiss Axioscan.Z1 and Zen 2 software. Images were dearrayed and analyzed for fluorescence intensity and area using QuPath 0.3.0 software. For each tissue section, fluorescence intensity was normalized to the fluorescent area (data S6). A threshold of fluorescence was set to 500, and values below that level were eliminated from the analysis. High-resolution full scan images of the TMA slide are available through the zenodo repository with the DOI 10.5281/zenodo.7149674.

### Cell proliferation assays

To determine the rate of cell proliferation, 25,000 cells per well were seeded into 48-well plates in DMEM without phenol red, containing 5% CHFBS, penicillin/streptomycin, and l-glutamine. After 24 hours, one plate was harvested as a control before treatment (day 0), and the rest of the plates were treated with vehicle (ethanol), E2 (100 pM), E2 (100 pM) + 4-OHT (100 nM), and E2 (100 pM) + ICI (100 nM). Plates were harvested after 2, 3, and 4 days from seeding for MCF7-V cells and 2, 4, and 6 days for T47D cells. The proliferation of cells was quantitated by crystal violet staining (see above).

### Wound-healing assay

Cells were seeded into six-well plates. At 100% confluency, wounds were formed by scratching the cell layers using a 200-μl pipette tip. Cells were then washed with tris-buffered saline (TBS) and incubated in DMEM without phenol red, containing 5% CHFBS, penicillin/streptomycin, and l-glutamine. Images of specific areas of the wounds were taken at 0, 14, and 24 hours after scratching. Image analysis was done by measuring the wound area using the software ImageJ. % Wound healing was calculated according to the following formula: 100 × [wound area at time 0 − wound area at time (i)]/wound area at time 0.

### Gene expression analyses

#### Drug treatments

Cells were hormone-deprived before treatments for 72 hours and seeded at a density of 200,000 cells per well of a six-well plate. Then, cells were treated with ethanol, E2 (1 nM), and E2 (1 nM) + 4-OHT (100 nM) for 24 hours. Assays to determine the basal level activities of ERα without any induction or drug treatment were done in hormone-deprived medium. For the treatments with purine nucleosides, cells were incubated in a medium containing purine-depleted serum for 72 hours and then treated with vehicle [dimethyl sulfoxide (DMSO)], adenosine (5 μM), guanosine (5 μM), inosine (5 μM), or a combination of the three nucleosides.

#### RNA extraction and real-time RT-qPCR

RNA extraction was done using a homemade guanidinium-acid-phenol reagent (*107*). Cells (0.5 × 10^6^) were lysed in 500 μl of solution D [4 M guanidium isothiocyanate, 25 mM sodium citrate (pH 7) and 0.5% N-laurosylsarcosine, and 0.1 M β-mercaptoethanol] and then transferred to microfuge tubes. Fifty microliters of 2 M sodium acetate (pH 7.4), 500 μl of water-saturated phenol (ROTH, no. A980.1), and 100 μl of chloroform/isoamyl alcohol (49:1) (Merck, no. 25668) were added, and tubes were shaken vigorously. After centrifugation (20 min at 10,000*g*, 4°C), the top aqueous phase (about 400 μl) containing the total RNA was recovered and precipitated with 250 μl of isopropanol, and RNA pellets were washed with 250 μl of 75% ethanol. RNA was quantitated using a NanoDrop. The total RNA (400 ng) was reverse-transcribed into cDNA using random primers (Promega, no. C1181) and a GoScript reverse transcriptase kit (Promega, no. A5003). Quantitative real-time PCR (qPCR) reactions were done using specific primers and a GoTaq qPCR Master Mix kit (Promega, no. A6001), according to the manufacturer’s protocol. Cycle threshold (Ct) values were measured using a Bio-Rad CFX 96 Real-Time PCR instrument (Bio-Rad). Relative gene expression was calculated using the ΔΔCt method. Specific primer sequences are listed in table S2.

### Protein expression analyses

#### Drug treatments and transient transfections

Assays of the basal levels of ERα were done in a hormone-deprived medium to stabilize the receptor. For treatments with purine nucleosides, cells were preincubated in hormone-deprived medium for 72 hours for ERα assays or in a medium containing purine-depleted serum for the assays of mTOR signaling markers. Then, cells were treated with vehicle (DMSO), adenosine, guanosine, inosine, or a combination of the three nucleosides. Nucleosides were added at 5 μM each for assays in purine-depleted medium, whereas excess nucleosides at 100 μM were used for assays in hormone-deprived but purine-rich medium. For CREB1 phosphorylation assays, cells were treated with FI cocktail that contains 10 μM forskolin (Cayman Chemical, no. 11018) and 100 μM IBMX (Sigma-Aldrich, no. 410957) and incubated for 30 min, 4 hours, 12 hours, and 24 hours. For assays with MRT, cells were treated/pretreated with MRT (500 nM) for 24 hours. For the transient expression of sgNT and sgPAICS in PAICS-overexpressing MCF7-V clones, cells were hormone-deprived for 72 hours, seeded in a six-well plate, and transfected with either sgRPE65 or sgPAICS plasmids using PEI MAX 40K at a DNA:PEI MAX ratio of 1:6.

#### Immunoblot analyses

Cells were harvested, and pellets were washed at least twice with PBS and then lysed in ice-cold lysis buffer [20 mM tris-HCl (pH 7.4), 2 mM EDTA, 150 mM NaCl, 1.2% sodium deoxycholate, 1.2% Triton X-100, protease inhibitor cocktail (Thermo Fisher Scientific, no. 78429), and phosphatase inhibitor cocktail (Thermo Fisher Scientific, no. 78420)]. Cell lysates were sonicated for 15 min at high power with the Bioruptor sonicator (Diagenode). Protein quantification was performed using the Bradford reagent (Bio-Rad, no. 5000001), measuring absorbance at 595 nm. Proteins (20 to 50 μg) were resolved on SDS–polyacrylamide gel electrophoresis and transferred to a nitrocellulose membrane (GVS Life Science) with an electroblotting unit (VWR, no. BTV100). Membranes were blocked with 2.5% nonfat milk or BSA in TBS containing 0.2% Tween 20 (TBST) and incubated with primary antibodies overnight at 4°C. Then, they were washed with TBST, incubated with the corresponding secondary antibodies for 1 hour at room temperature, and developed using the WesternBright chemiluminescent substrate (Advansta, no. K-12045-D50). Images were captured using an Amersham ImageQuant 800 biomolecular imager. The “Fisher BioReagents EZ-Run Prestained Rec Protein Ladder” (Thermo Fisher Scientific) was used as protein molecular weight marker. Quantification of band intensities was done using ImageJ. Details of primary and secondary antibodies are listed in table S3. Raw uncropped blots are available in data S7.

### Puromycin labeling assay

WT MCF7-V cells and the MCF7-V clones PAICS NC, PAICS OE 25, and PAICS OE 45 were seeded at 60% confluency in 10-cm dishes. MRT was added to the cells for 24 hours followed by puromycin (1 μM) for specific periods. Cells were harvested and lysed in 20 mM tris-HCl (pH 7.4), 2 mM EDTA, 150 mM NaCl, 1.2% sodium deoxycholate, 1.2% Triton X-100, and protease inhibitor cocktail. Immunoblotting was done using an anti-puromycin antibody (table S3).

### Dual-luciferase reporter assays

For assays with HEK 293 T cells, they were seeded at 60% confluency in six-well plates. After hormone deprivation for 72 hours, cells were cotransfected using PEI MAX 40K with 0.75 μg of the luciferase reporter plasmid ERE-Luc, 50 ng of Renilla luciferase expression plasmid pRL-CMV used as a transfection control, and 1 μg of either pPAICS-EGFP or pEGFP-C1 as a control plasmid. Each transfection mixture was further divided to receive 0.15 μg of either plasmids HEG0 (*101*) or its derivatives HEG0 S305A and HEG0 S104/106A, or their expression vector pSG5 (*102*) as the empty vector control. Twenty-four hours after transfection, cells were trypsinized and seeded into 96-well plates and then treated with either ethanol, E2 (1 nM), E2 + 4-OHT (100 nM), or E2 + ICI (100 nM) for another 24 hours. For assays with MCF7-V cells, WT cells or the sgPAICS 38 knockdown clone were preincubated in a medium containing purine-depleted (Thermo Fisher Scientific, no. A3382001) or purine-supplemented (Thermo Fisher Scientific, no. A3160501) serum for 72 hours. Then, cells were seeded in six-well plates and cotransfected with 1 μg of ERE-Luc and 50 ng of pRL-CMV using PEI MAX 40K. After 24 hours, cells were trypsinized; reseeded into 96-well plates; and treated with vehicle (DMSO), adenosine (5 μM), guanosine (5 μM), inosine (5 μM), or a combination of the three nucleosides, in the presence and the absence of E2 (5 pM). For luciferase assays with CRE-Luc, WT MCF7-V cells and the knockdown clones sgPAICS 28 and sgPAICS 38 were cotransfected with 1 μg of CRE-Luc reporter plasmid and 50 ng of pRL-CMV using PEI MAX 40K. After 24 hours, cells were trypsinized, reseeded into a 96-well plate, and treated with either FI or vehicle (DMSO) for another 24 hours. Cells were lysed, and luciferase activities were measured with a dual-luciferase detection kit (Promega, no. E1910), using a bioluminescence plate reader (Cytation 3 Imaging Reader, Agilent). The activity of firefly luciferase was normalized to the activity of the Renilla luciferase.

### ATP assay

ATP levels were measured with the CellTiter-Glo (CTG) luminescent assay (Promega, no. G7572) according to the manufacturer’s instructions. Briefly, cells were seeded at a density of 70,000 cells per well of 96-well plates. After 24 hours, CTG reagent was added, and the luminescence was measured with the Cytation 3 Imaging Reader from Agilent. The luminescence of control cells was set to 1.

### cAMP-Glo assay

cAMP was measured using a cAMP-Glo assay kit (Promega, no. V1501) according to the manufacturer’s protocol. Briefly, cells were seeded at a density of 3000 cells per well of a white opaque 384-well plate with a clear bottom (Corning, no. 3765). After 24 hours, medium was replaced by serum-free medium containing FI and incubated for 30 min, 1 hour, 3 hours, and 6 hours. Cells were lysed using the cAMP-Glo lysis buffer by shaking at room temperature for 15 to 30 min. cAMP detection solution containing the enzyme PKA was added, mixed, and incubated for 20 min at room temperature. Then, the Kinase-Glo reagent was added, mixed, and incubated for 10 min at room temperature. Luminescence was measured using the Cytation 3 Imaging Reader from Agilent.

### PKA-SPARK fluorescence imaging

Cells were seeded at 200,000 cells per well of a six-well plate and transiently transfected with 1 μg of pPKA-SPARK using the PEI MAX 40K transfection reagent. Twenty-four hours after transfection, cells were treated with FI for specified time points. Fluorescence imaging and image analysis were done on live cells using the Cytation 3 Imaging Reader (Agilent) the software Gen5.

### 4-OHT and MRT dose-response curves

Cells were seeded into a 96-well plate 24 hours before drug treatments. A serial dilution ranging from 10^−9^ to 10^−4^ M for 4-OHT or MRT in DMEM medium without phenol red, containing 5% CHFBS, penicillin/streptomycin, l-glutamine, and E2 (5 pM), was prepared and added to the cells. For the combination of 4-OHT + MRT, 50 nM or 120 nM MRT was added to the serial dilutions of 4-OHT. For the combination of 4-OHT and purine nucleosides, purine-depleted serum was charcoal-treated for the additional depletion of hormones. After 7 days of 4-OHT, 4-OHT + MRT, or 4-OHT + nucleoside (5 μM each) treatments or 3 days of MRT monotherapy, cells were stained using crystal violet, and absorbance was measured at 595 nm using the Cytation 3 Imaging Reader (Agilent). Dose-response curves were fitted by least squares regression with a variable slope (four parameters) using GraphPad Prism version 8.0.0. IC_10_, IC_20_, and IC_50_ values were obtained by interpolating the curves with a 95% confidence interval.

### Kaplan-Meier analyses

Graphs for OS and RFS associated with *PAICS* mRNA expression in ERα^+^ (+/− tamoxifen treatment) and ERα^−^ breast cancer datasets were generated using the Kaplan-Meier plotter (*40*).

### Statistical analyses

Data were analyzed using GraphPad Prism 8.0.0 and Microsoft Excel. Differences between two groups were analyzed with a two-tailed Student’s *t* test. One-way analysis of variance (ANOVA) was used to analyze variations between tissue samples of the TMA scan. Two-way ANOVA was used to analyze differences of multiple variables between more than two groups. Error bars represent the SD between replicates, and *P* values of <0.05 were considered statistically significant. All numerical data used to generate the figures are provided in data S8.

## Notes

View/request a protocol for this paper from *Bio-protocol*.
