# Supplementary material for: CRISPR-Cas9 screen reveals a role of purine synthesis for estrogen receptor α activity and tamoxifen resistance of breast cancer cells
Source: Sci Adv. 2023 May 12;9(19):eadd3685. doi: 10.1126/sciadv.add3685 (PMC10181187; doi:10.1126/sciadv.add3685)
Supplement: Supplementary file 1 — Figs. S1 to S10 Tables S1 to S3 Legends for data S1 to S8 [file sciadv.add3685_sm.pdf]

Science Advances
AAAS

Supplementary Materials for

CRISPR-Cas9 screen reveals a role of purine synthesis for estrogen receptor α activity and tamoxifen resistance of breast cancer cells

Dina Hany *et al*.

Corresponding author: Didier Picard, didier.picard@unige.ch

Sci. Adv. **9**, eadd3685 (2023)
DOI: 10.1126/sciadv.add3685

The PDF file includes:

Figs. S1 to S10
Tables S1 to S3
Legends for data S1 to S8

Other Supplementary Material for this manuscript includes the following:

Data S1 to S8

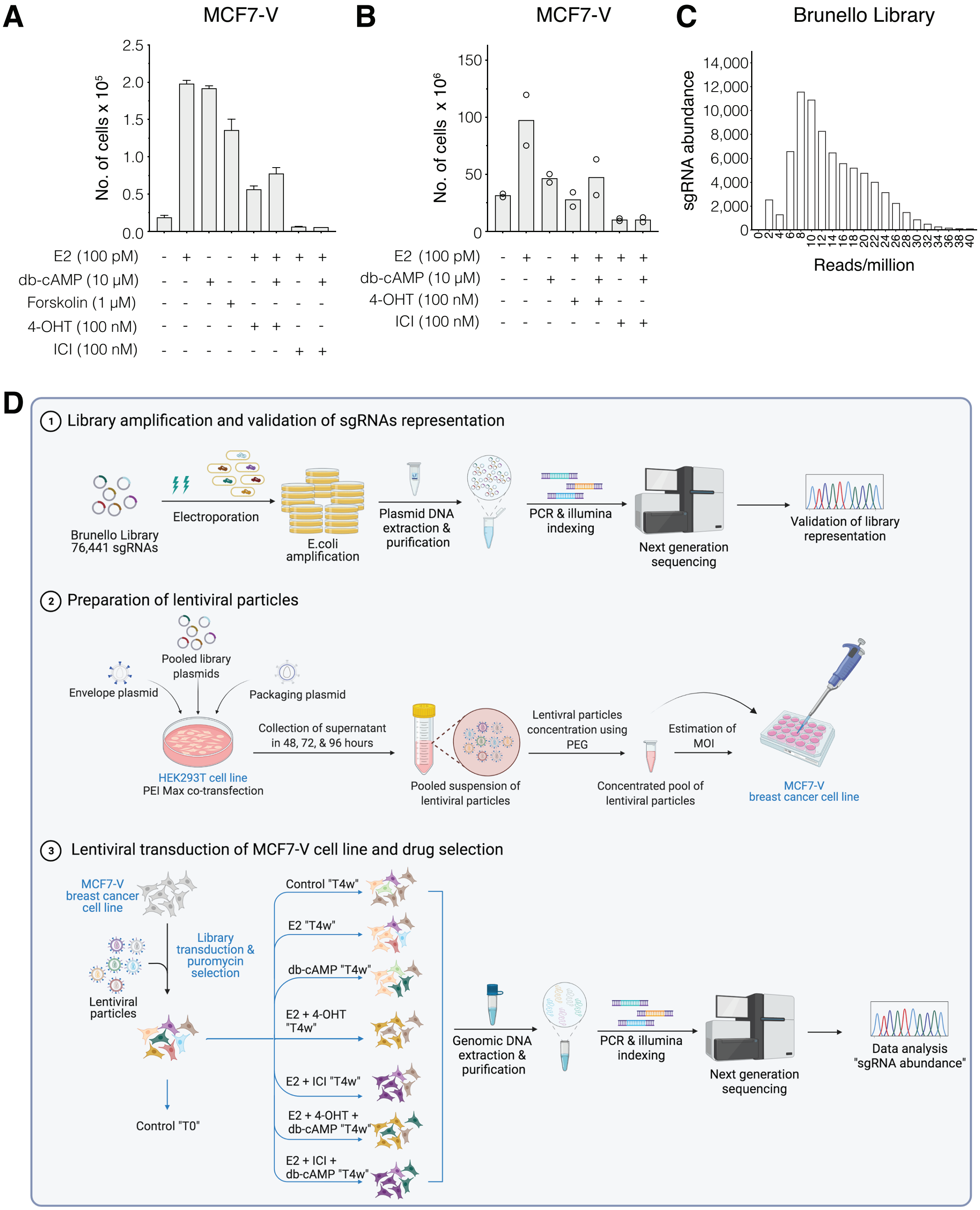
A
MCF7-V
No. of cells x 10^5
E2 (100 pM)
db-cAMP (10 μM)
Forskolin (1 μM)
4-OHT (100 nM)
ICI (100 nM)
B
MCF7-V
No. of cells x 10^6
C
Brunello Library
sgRNA abundance
Reads/million
D
1 Library amplification and validation of sgRNAs representation
Brunello Library
76,441 sgRNAs
Electroporation
E.coli
amplification
Plasmid DNA
extraction &
purification
PCR & illumina
indexing
Next generation
sequencing
Validation of library
representation
2 Preparation of lentiviral particles
Pooled library
plasmids
Envelope plasmid
Packaging plasmid
HEK293T cell line
PEI Max co-transfection
Collection of supernatant
in 48, 72, & 96 hours
Pooled suspension of
lentiviral particles
Lentivral particles
concentration using
PEG
Concentrated pool of
lentiviral particles
Estimation of
MOI
MCF7-V
breast cancer cell line
3 Lentiviral transduction of MCF7-V cell line and drug selection
MCF7-V
breast cancer
cell line
Library
transduction &
puromycin
selection
Lentiviral
particles
Control "T0"
Control "T4w"
E2 "T4w"
db-cAMP "T4w"
E2 + 4-OHT
"T4w"
E2 + ICI "T4w"
E2 + 4-OHT +
db-cAMP "T4w"
E2 + ICI +
db-cAMP "T4w"
Genomic DNA
extraction &
purification
PCR & illumina
indexing
Next generation
sequencing
Data analysis
"sgRNA abundance"

Fig. S1. Details of CRISPR/Cas9 screen. (A) Bar graph representing the means of the number of MCF7-V cells after 2 weeks with the indicated treatments, measured by a crystal violet assay; cell numbers were interpolated from a standard curve. Error bars represent standard deviations of the means of at least 3 independent experiments. **(B)** Bar graph representing the cell counts of MCF7-V at the end of the primary CRISPR/Cas9 screen. Cells from two independent biological replicates were transduced with the Brunello library, treated as indicated for 4 weeks, and counted using a hemocytometer and an inverted light microscope. **(C)** Bar graph indicating the distribution and abundance of the sgRNAs after library amplification and NGS. **(D)** Schematic illustration of the detailed steps of the primary CRISPR/Cas9 screen. The scheme was created with Biorender.com.

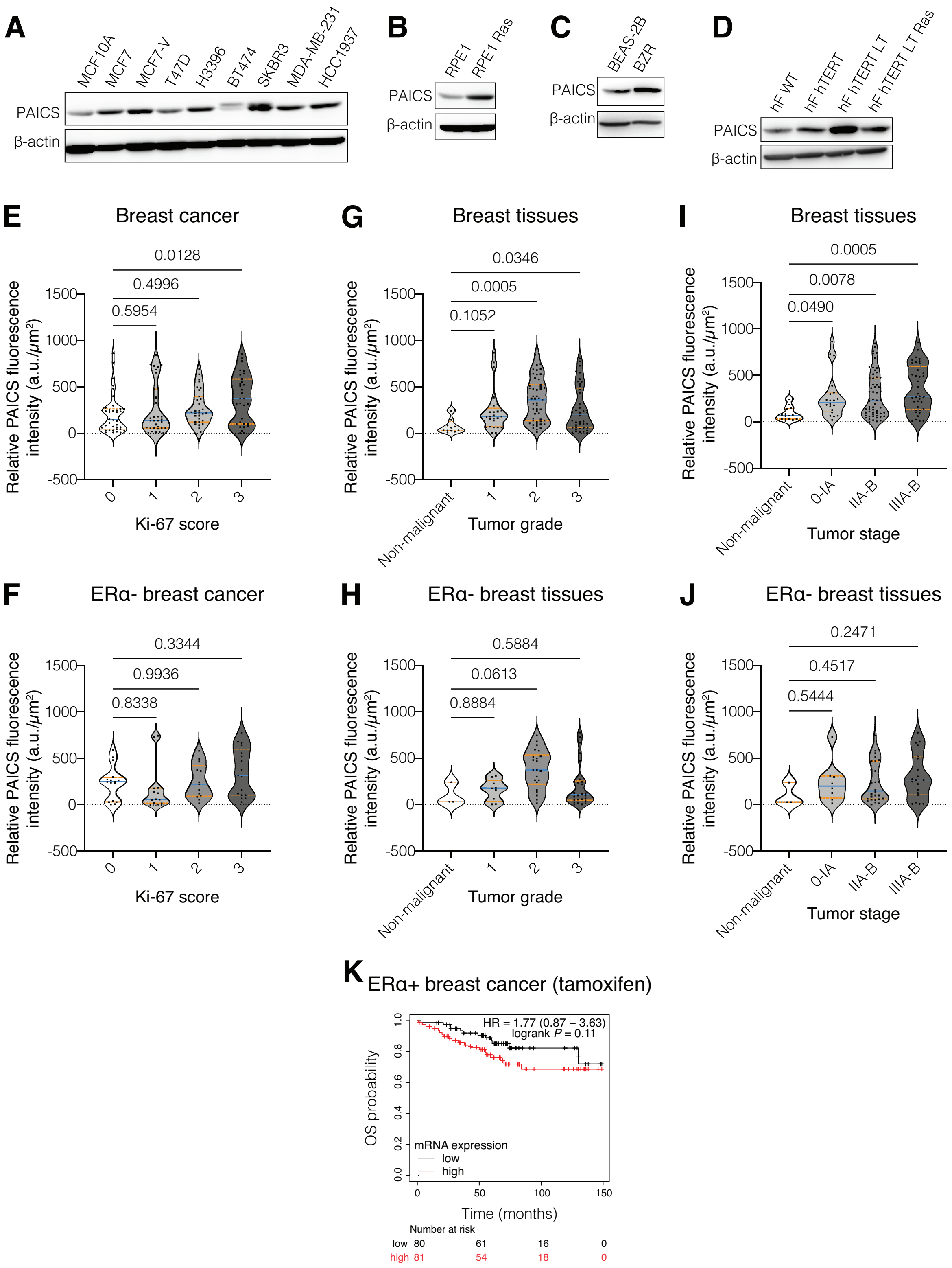
A
MCF10A
MCF7
MCF7-V
T47D
H3396
BT474
SKBR3
MDA-MB-231
HCC1937
PAICS
β-actin
B
RPE1
RPE1 Ras
PAICS
β-actin
C
BEAS-2B
BZR
PAICS
β-actin
D
hF WT
hF hTERT
hF hTERT LT
hF hTERT LT Ras
PAICS
β-actin
E
Breast cancer
0.0128
0.4996
0.5954
Relative PAICS fluorescence intensity (a.u./μm²)
Ki-67 score
G
Breast tissues
0.0346
0.0005
0.1052
Non-malignant
Tumor grade
I
Breast tissues
0.0005
0.0078
0.0490
0-IA
IIA-B
IIIA-B
Tumor stage
F
ERα- breast cancer
0.3344
0.9936
0.8338
Ki-67 score
H
ERα- breast tissues
0.5884
0.0613
0.8884
Tumor grade
J
ERα- breast tissues
0.2471
0.4517
0.5444
Tumor stage
K
ERα+ breast cancer (tamoxifen)
HR = 1.77 (0.87 – 3.63)
logrank P = 0.11
OS probability
mRNA expression
low
high
Time (months)
Number at risk
low 80 61 16 0
high 81 54 18 0

Fig. S2. PAICS expression levels are upregulated in cancer, and correlate with progression and poor clinical outcomes of ERα+ breast tumors. (A) Immunoblot of PAICS in total cell lysates of a panel of breast cancer cell lines and the non-malignant cell line MCF10A. **(B to D)** Immunoblots of PAICS. **(E to J)** Violin plots of the means of the relative fluorescence intensities of PAICS (in arbitrary units (a.u.)) per fluorescent unit area (in μm^2) and and its 95% confidence interval between cores classified based on Ki-67 score (panels E and F), tumor grade (panels G and H), and TNM stage (panels I and J). The statistical significance between the groups was analyzed by one-way ANOVA, and p-values < 0.05 were considered statistically significant. **(K)** Kaplan-Meier plot of the overall survival (OS) data of ERα+ breast cancer patients who received tamoxifen therapy, classified as tumors expressing high levels (red line) and low levels (black line) of *PAICS* mRNA.

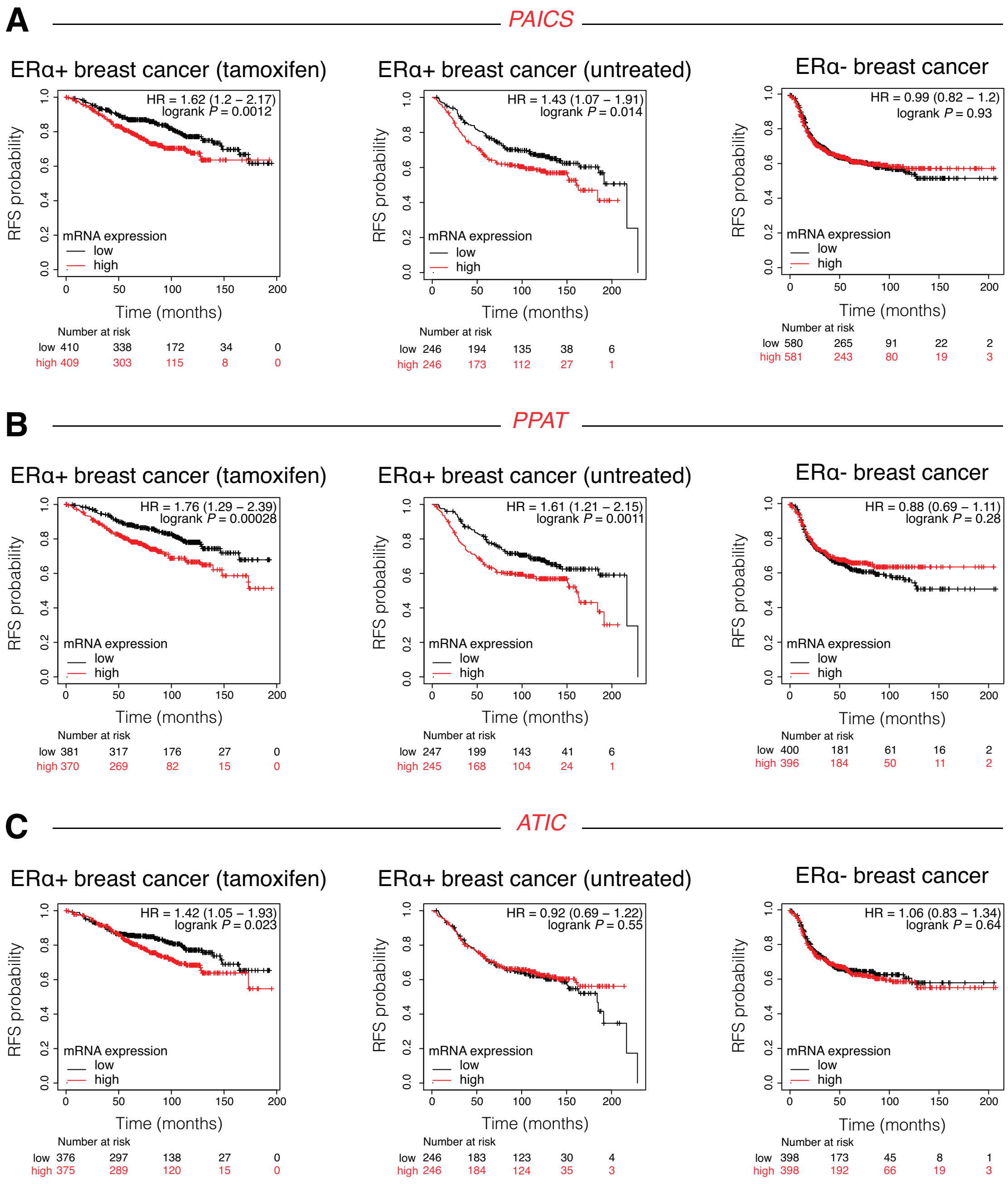

Fig. S3. High gene expression levels of *PAICS*, *PPAT*, and *ATIC* correlate with poor relapse-free survival with tamoxifen treatment. (A to C) Kaplan-Meier plots of the RFS data of ERα+ breast cancer patients who received tamoxifen therapy (left panel) or not (middle panel), and those with ERα- breast tumors. Data are classified as tumors expressing high (red line) and low (black line) levels of the respective mRNA.

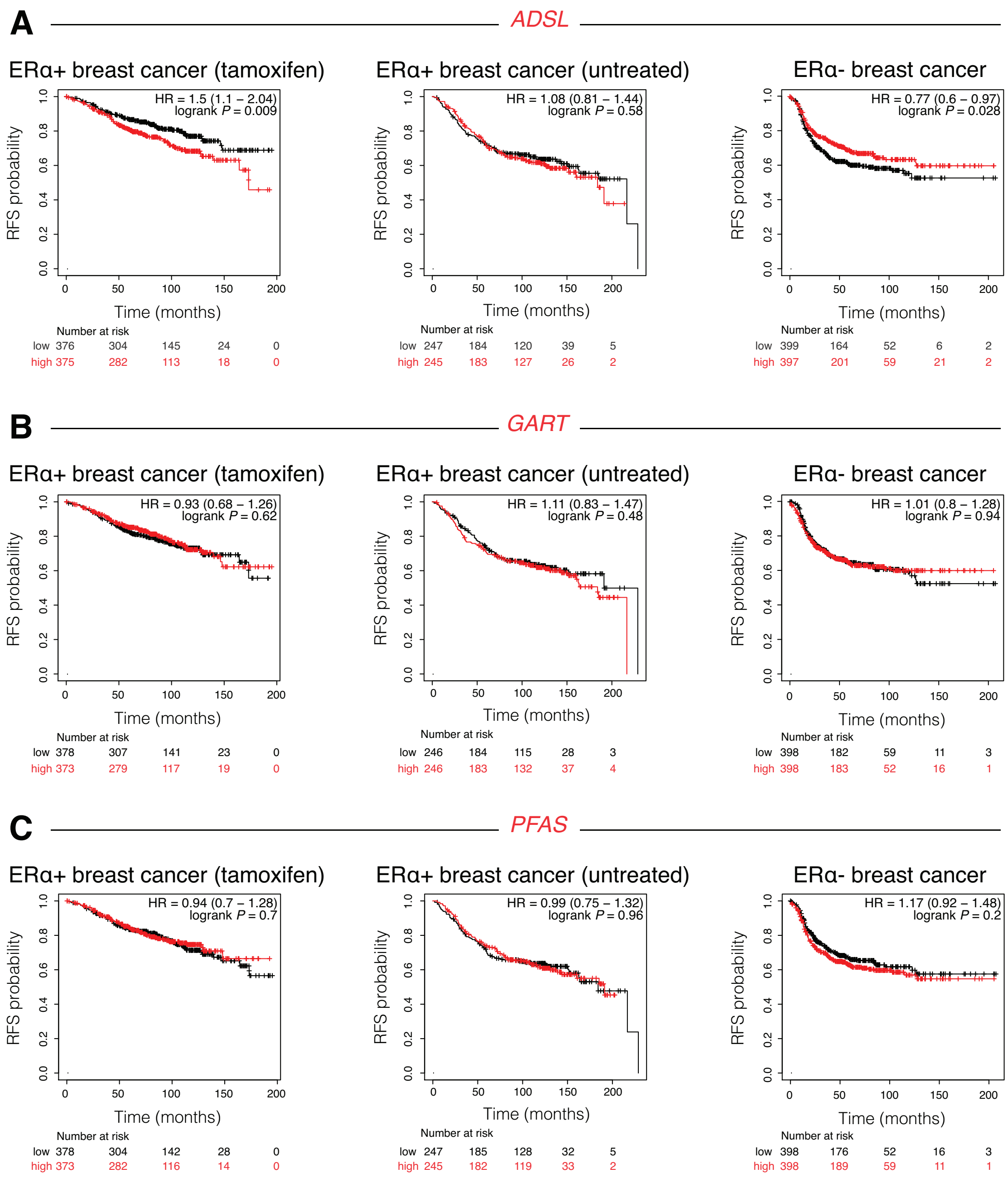

Fig. S4. High gene expression levels of *ADSL*, but not *GART* and *PFAS*, correlate with poor relapse-free survival with tamoxifen treatment. (A to C) Kaplan-Meier plots of the RFS data of ERα+ breast cancer patients who received tamoxifen therapy (left panel) or not (middle panel), and those with ERα- breast tumors. Data are classified as tumors expressing high (red line) and low (black line) levels of the respective mRNA.

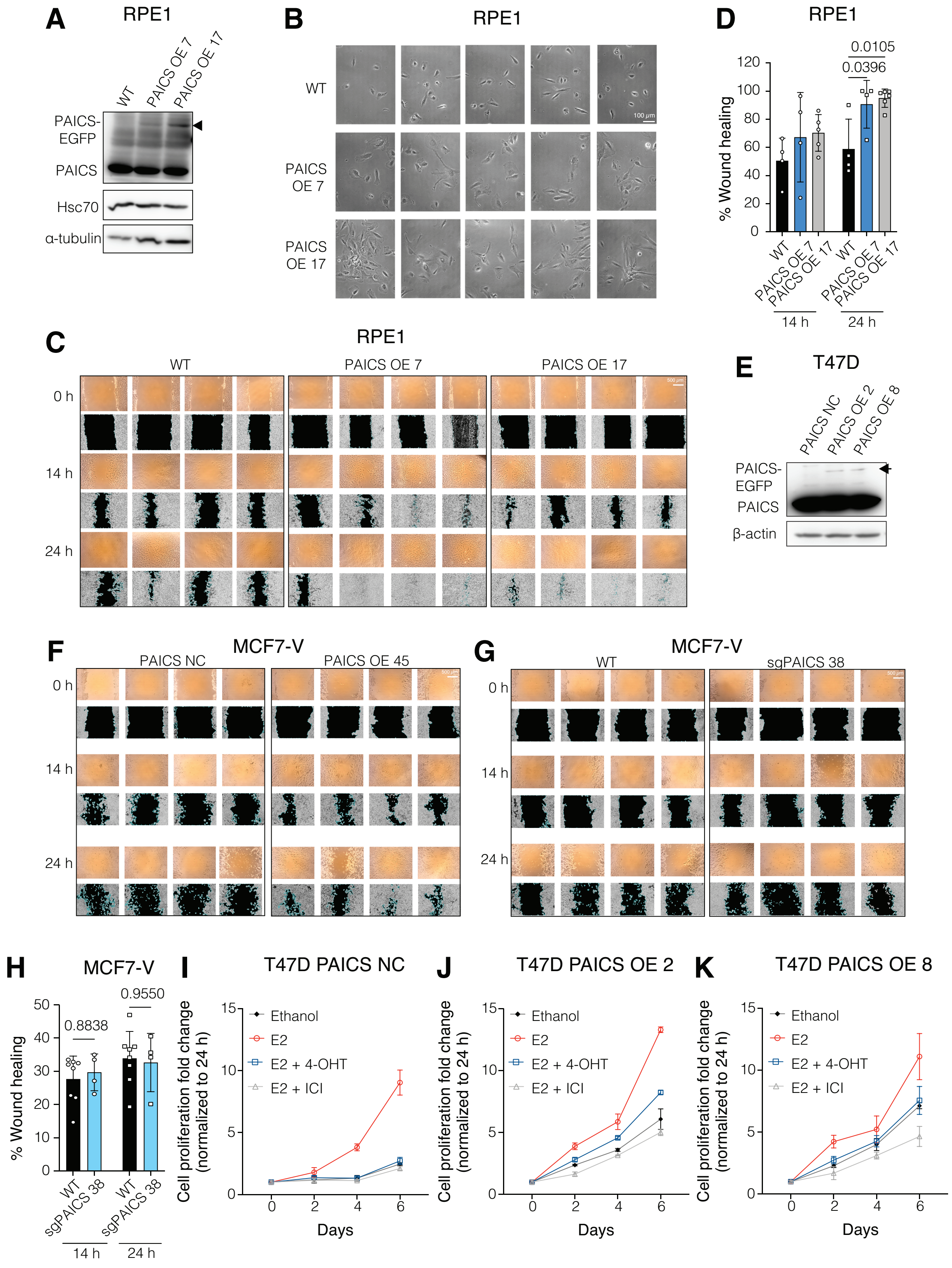
A
RPE1
WT
PAICS OE 7
PAICS OE 17
PAICS-EGFP
PAICS
Hsc70
α-tubulin
B
RPE1
WT
PAICS OE 7
PAICS OE 17
100 μm
D
RPE1
0.0105
0.0396
% Wound healing
14 h
24 h
C
RPE1
WT
PAICS OE 7
PAICS OE 17
0 h
14 h
24 h
E
T47D
PAICS NC
PAICS OE 2
PAICS OE 8
PAICS-EGFP
PAICS
β-actin
F
MCF7-V
PAICS NC
PAICS OE 45
0 h
14 h
24 h
G
MCF7-V
WT
sgPAICS 38
0 h
14 h
24 h
H
MCF7-V
0.8838
0.9550
% Wound healing
WT
sgPAICS 38
14 h
24 h
I
T47D PAICS NC
J
T47D PAICS OE 2
K
T47D PAICS OE 8
Ethanol
E2
E2 + 4-OHT
E2 + ICI
Cell proliferation fold change (normalized to 24 h)
Days

Fig. S5. PAICS expression correlates with cell migration and estrogen-independent and tamoxifen-resistant proliferation of ERα+ breast cancer cells. (A) Immunoblots of endogenous PAICS and exogenous PAICS-EGFP. Hsc70 and α-tubulin were used as internal controls. **(B)** Cellular morphology of RPE1 cells as indicated. **(C, F** and **G)** Cell migration assays. Raw and ImageJ-processed images of the replicates of the scratch wound healing assays. Phase-contrast images were captured at the time of the scratch (0 h), after 14 h and 24 h using an inverted light microscope. **(D** and **H)** Bar graph showing the % wound healing as quantitated with the software ImageJ. **(E)** Immunoblots of endogenous PAICS and exogenous PAICS-EGFP expressed in T47D cell derivatives as indicated. **(I to K)** Cell proliferation of T47D cell derivatives as indicated, treated with either vehicle (ethanol), E2 (100 pM), E2 (100 pM) + 4-OHT (100 nM), or E2 (100 pM) + ICI (100 nM), measured with a crystal violet assay. For panels D, H, and I to K, data are represented as means ± SD of at least 3 independent experiments. The statistical significance between the groups was analyzed by two-way ANOVA (D and H), and p-values < 0.05 were considered statistically significant.

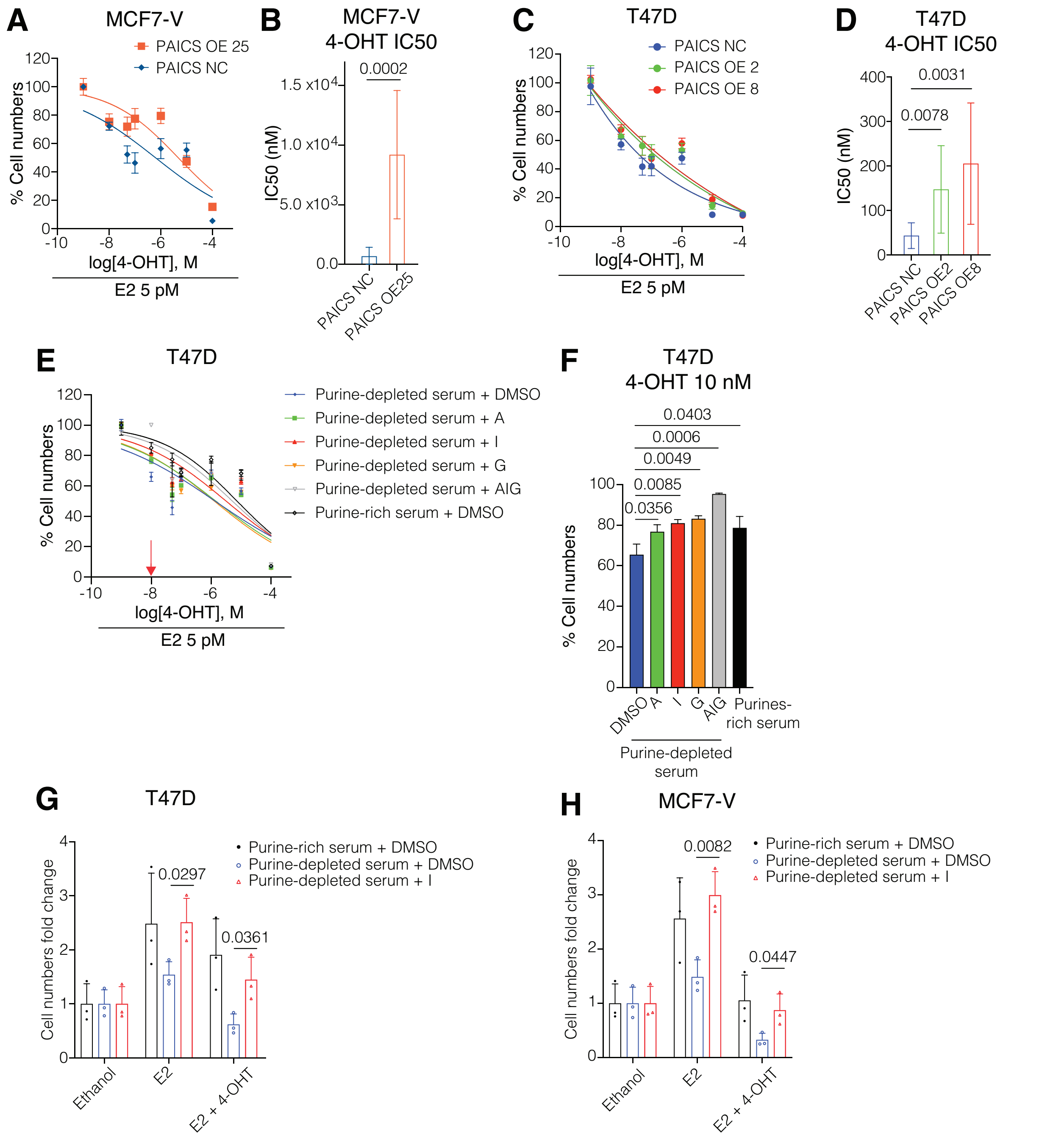

Fig. S6. High PAICS expression and purine nucleoside levels correlate with tamoxifen resistance in breast cancer cells. (A) Dose-response curve with increasing doses of 4-OHT with PAICS OE 25 and PAICS NC cells. **(B)** 4-OHT IC50 values interpolated from the 4-OHT dose-response curves of panel A; the data are represented as the interpolated means ± 95% CI. **(C)** 4-OHT dose-response curves with PAICS-overexpressing clones of T47D cells. **(D)** As in B, 4-OHT IC50 values interpolated from the 4-OHT dose-response curves of panel C. **(E)** 4-OHT dose-response experiment with T47D cells, with the addition of purine nucleosides as indicated (at 5 µM each). **(F)** 4-OHT IC30 values interpolated from the 4-OHT dose-response curves of panel E.

(G and H) Growth assays with T47D (panel G) and MCF7-V (panel H) cells treated with E2 (100 pM) or E2 + 4-OHT (100 nM), supplemented as indicated with 5 μM inosine (I). For panels A, C, E, G, and H, values were measured with a crystal violet assay. The result for a vehicle-treated control group was set to 100% (panels A, C, and E) or to 1 (panels G and H). Data are represented as means ± SD of at least 3 independent experiments. The statistical significance between the groups was analyzed by unpaired Student's t-tests (B and D), and *p*-values < 0.05 were considered statistically significant.

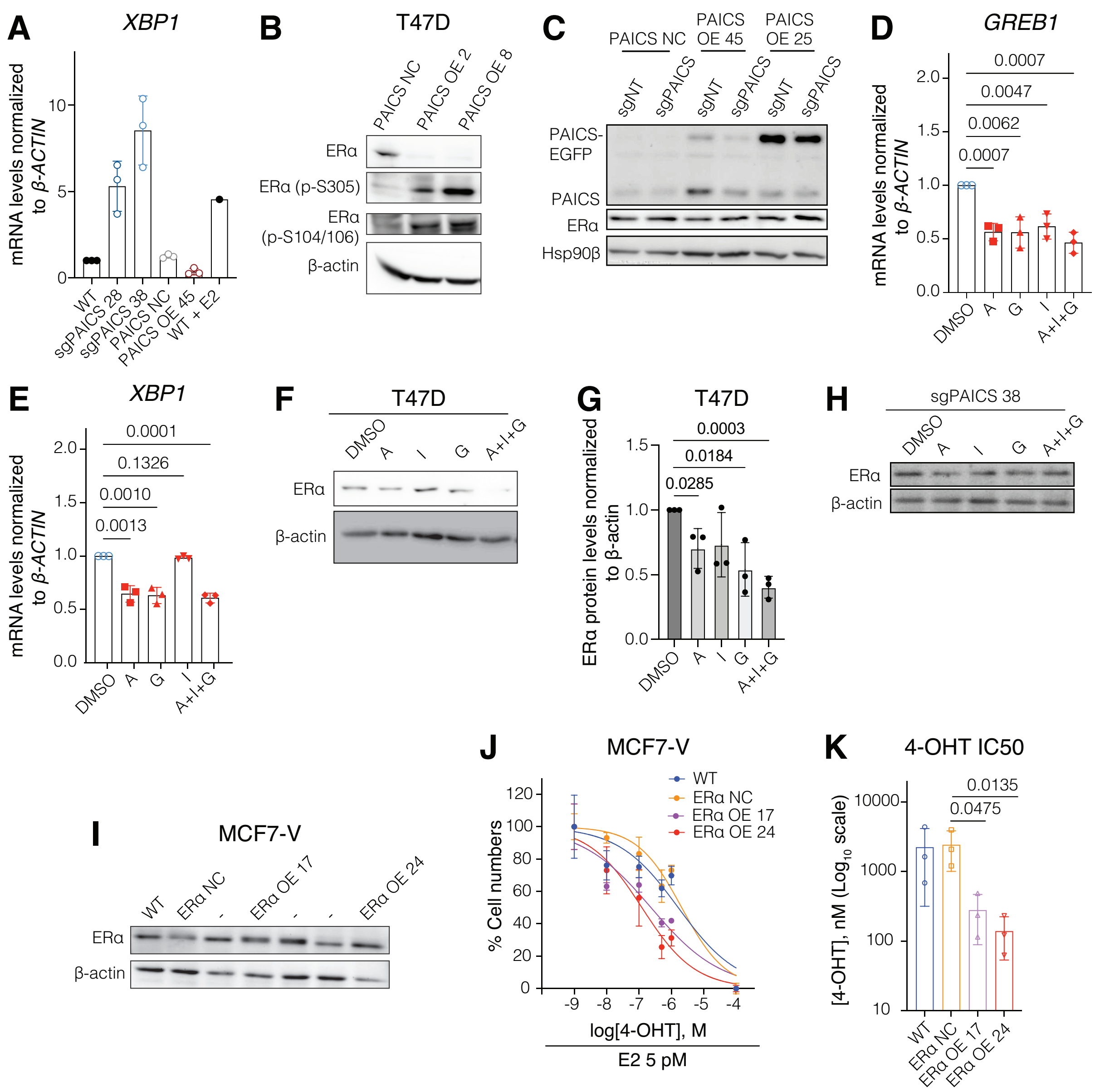

Fig. S7. Interplay between PAICS and ERα. (A) mRNA levels of the ERα target gene *XBP1*. MCF7-V WT cells treated with E2 (1 nM) were considered as a positive control. Ct values were first normalized to *β-ACTIN*, then values corresponding to MCF7 WT were set to 1. **(B)** Immunoblots of phosphorylated (S305) and (S104/106), and total ERα in the indicated T47D cells **(C)** Immunoblots of PAICS and ERα. **(D** and **E)** mRNA levels of the ERα target genes *GREB1* and *XBP1* in MCF7-V cells. Cells were starved in medium containing purine-depleted serum for 72 h and then treated with the indicated nucleoside each at 5 µM. Ct values were first normalized to *β-ACTIN*, then values corresponding to vehicle were set to 1. **(F)** A representative immunoblot of ERα in total cell lysates of T47D cells, hormone-starved for 72 h and then treated with the indicated nucleoside each at 100 µM. **(G)** Quantification of ERα protein levels from 3 replicate blots as in panel F. **(H)** Immunoblot of ERα in the MCF7-V cell clone sgPAICS 38. Cells were

hormone-starved for 72 h and then treated with the indicated nucleoside each at 100 μM. **(I)** Immunoblot of ERα. Before the assay, cells were hormone-starved for 72 h to stabilize ERα protein levels. **(J)** Dose-response curves with increasing doses of 4-OHT. **(K)** 4-OHT IC50 values interpolated from the 4-OHT dose-response curves shown in panel J; the data are represented as the interpolated means ± 95% CI. For panels **A, D, E, G, J,** and **K,** the data are represented as means ± SD of at least 3 independent experiments. The statistical significance between the groups was analyzed by unpaired Student's t-tests (**D, E, G,** and **K)**, and *p*-values < 0.05 were considered statistically significant.

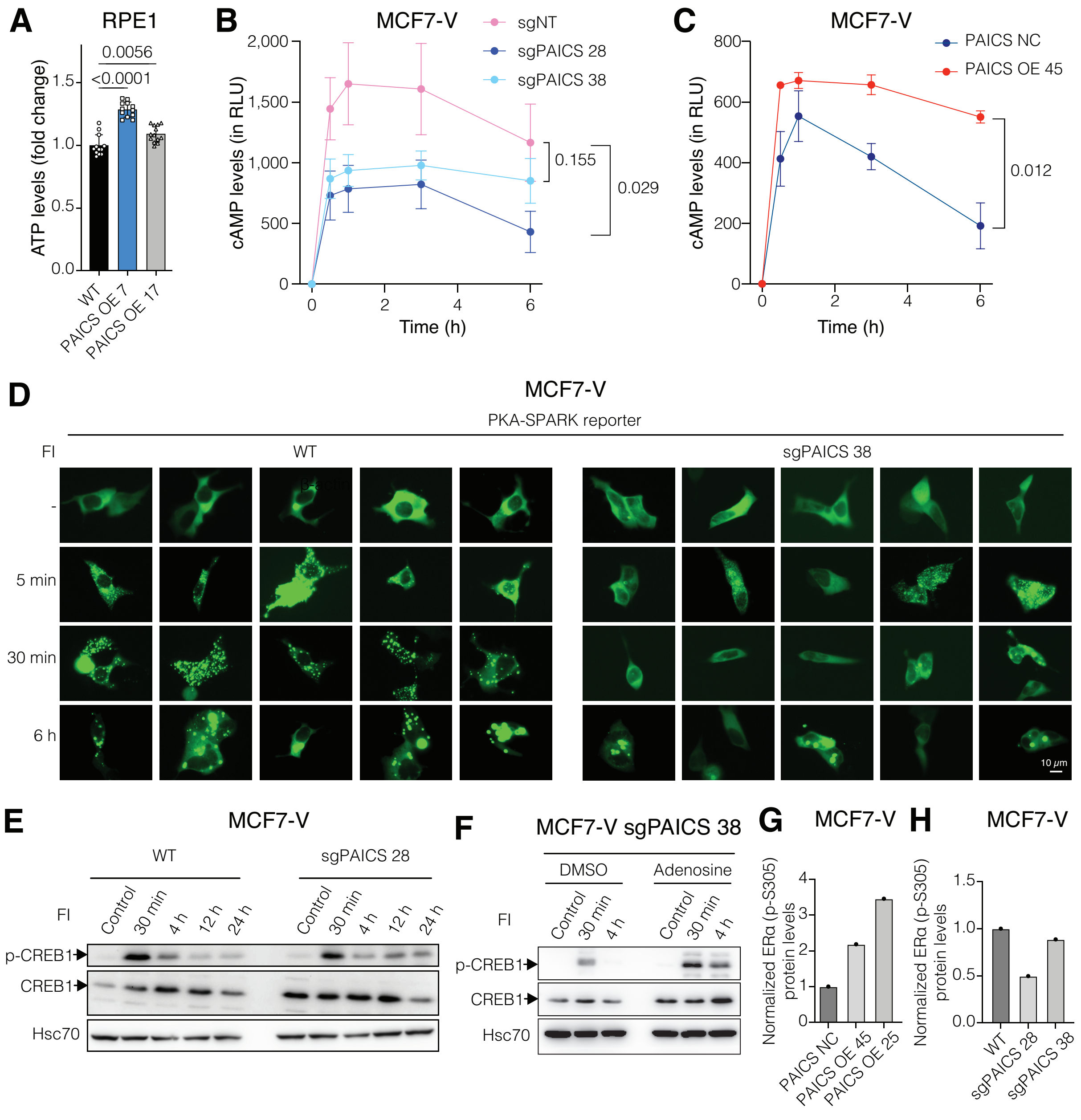

Fig. S8. Increased expression of PAICS correlates with the activation of cAMP/PKA in ERα+ breast cancer cells. (A) ATP levels of indicated cells. Luminescence signal of RPE1 WT cells was set to 1. **(B** and **C)** cAMP levels measured by cAMP-Glo reagent. Cells were treated with FI for 30 min, 1 h, 3 h, and 6 h. Experiment was based on at least 3 independent replicates. **(D)** Fluorescent images of cells transiently transfected with the PKA-SPARK reporter, and treated with FI for 5 min, 30 min, and 6 h to induce cAMP/PKA signaling. Green-fluorescent phase separation droplets are indicative of active PKA. **(E)** Immunoblots of phosphorylated (S133) and total CREB1. Cells were treated with FI for 30 min, 4 h, 12 h, and 24 h to induce the phosphorylation of CREB1 downstream of active PKA. **(F)** As in panel E, cells were treated with FI for 30 min and 4 h, after a prior treatment with or without adenosine (100 μM) for 24 h. **(G** and **H)** Quantification

of the immunoblots of Fig. 7, A and B, respectively. Band intensities of ERα (p-S305) were first normalized to the corresponding bands of the total ERα and then to that of β-actin. For panels A-C, data are represented as mean ± SD of at least 3 independent experiments. The statistical significance between the groups was analyzed by unpaired Student's t-tests (for panel A), and *p*-values < 0.05 were considered statistically significant.

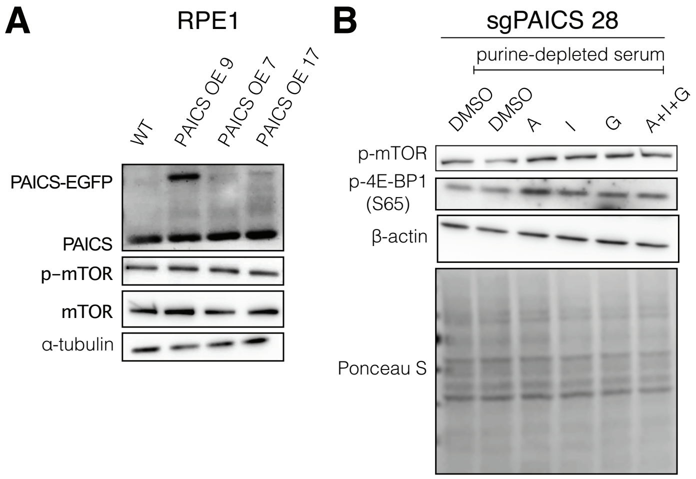

Fig. S9. Expression of PAICS is correlated with mTOR activity and the global rate of protein translation. (A) Immunoblots of the indicated proteins. **(B)** Immunoblots of the indicated protein levels in MCF7-V clone sgPAICS 28. Cells were purine-depleted for 72 h and then treated with the indicated nucleoside each at 5 μM. In parallel, a control group of cells that were not purine-starved were incubated in purine-rich medium for 72 h and then treated with DMSO.

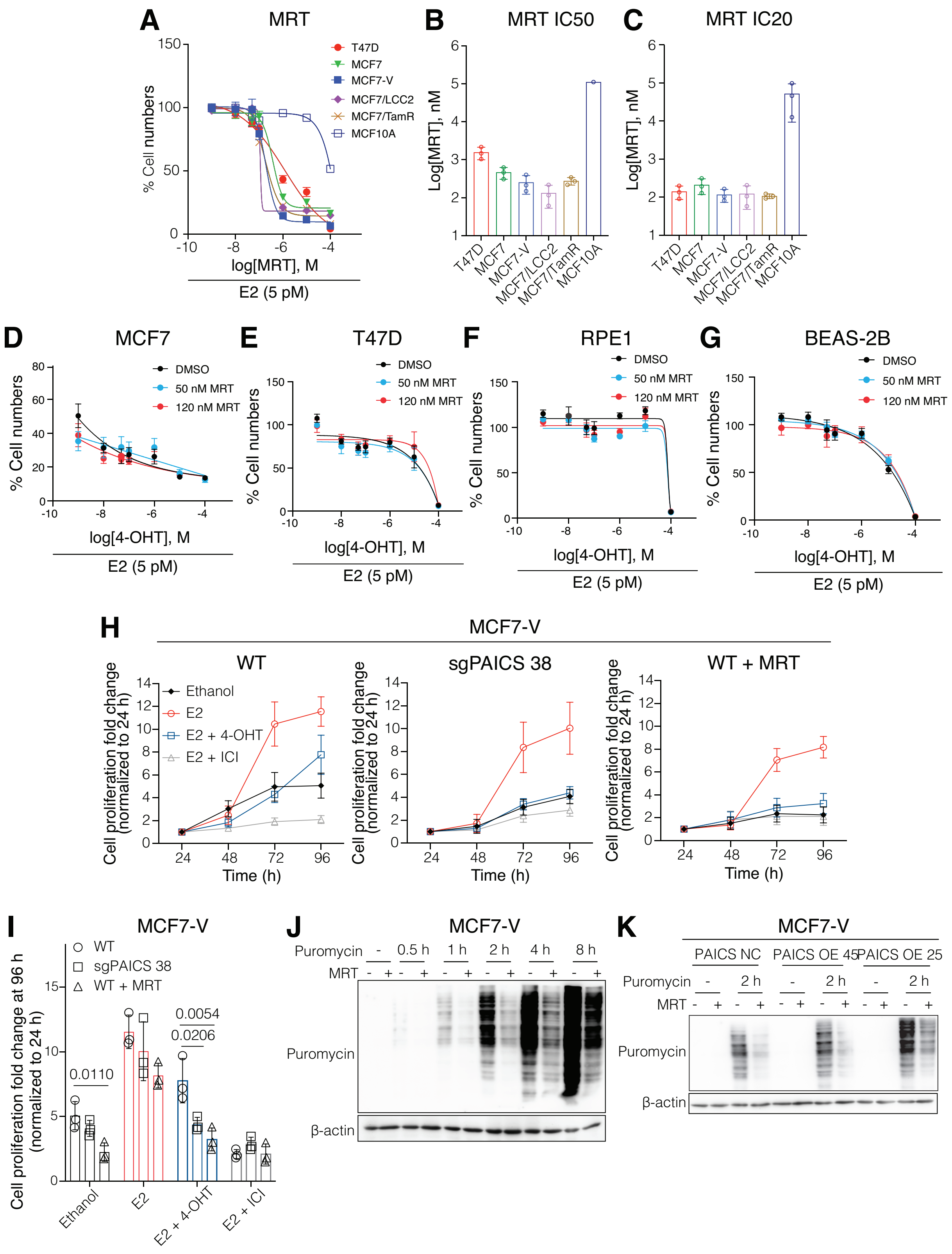

A
MRT
T47D
MCF7
MCF7-V
MCF7/LCC2
MCF7/TamR
MCF10A
% Cell numbers
log[MRT], M
E2 (5 pM)
B
MRT IC50
Log[MRT], nM
C
MRT IC20
Log[MRT], nM
D
MCF7
E
T47D
F
RPE1
G
BEAS-2B
DMSO
50 nM MRT
120 nM MRT
% Cell numbers
log[4-OHT], M
E2 (5 pM)
H
MCF7-V
WT
sgPAICS 38
WT + MRT
Ethanol
E2
E2 + 4-OHT
E2 + ICI
Cell proliferation fold change (normalized to 24 h)
Time (h)
I
MCF7-V
WT
sgPAICS 38
WT + MRT
Cell proliferation fold change at 96 h (normalized to 24 h)
0.0110
0.0054
0.0206
Ethanol
E2
E2 + 4-OHT
E2 + ICI
J
MCF7-V
Puromycin
MRT
0.5 h
1 h
2 h
4 h
8 h
β-actin
K
MCF7-V
PAICS NC
PAICS OE 45
PAICS OE 25
Puromycin
MRT
2 h
β-actin

Fig. S10. Pharmacological inhibition of PAICS sensitizes ERα+ breast cancer cells to 4-OHT. **(A)** Dose-response curves with increasing doses of MRT with the breast cancer cell lines T47D, MCF7, MCF7-V, MCF7/LCC2, and MCF7/TamR, and the non-malignant breast cells MCF10A. **(B** and **C)** Interpolated IC50 (panel B) and IC20 (panel C) values of MRT from the dose-response curves indicated in panel A. Data are represented as the interpolated means ± 95% CI. **(D to G)** Dose-response curves with increasing doses of 4-OHT with cell density measured with a crystal violet assay. For each curve, the result for a control group treated with vehicle was set to 100%. **(H)** Proliferation of WT MCF7-V cells, with or without MRT (100 nM) treatment. Cells were treated with either vehicle (ethanol), E2 (100 pM), E2 (100 pM) + 4-OHT (100 nM), or E2 (100 pM) + ICI (100 nM), measured with a crystal violet assay. The data are represented as means ± SD of at least 3 independent experiments. Proliferation of the clone sgPAICS 38 was used as control. **(I)** Quantification of the cell proliferation assay shown in panel H after 96 h. The statistical significance between the groups in each treatment condition was analyzed by one-way ANOVA, and p-values < 0.05 were considered statistically significant. **(J** and **K)** Immunoblot of puromycin-labelled nascent polypeptides. For panel J, MCF7-V WT cells were pre-treated with either vehicle (DMSO) or MRT (500 nM) for 24 h, then treated without or with puromycin (1 μM) for 0.5 h, 1 h, 2 h, 4 h, and 8 h. Cells in panel K were pre-treated with either vehicle (DMSO) or MRT (500 nM) for 24 h, then treated without or with puromycin (1 μM) for 2 h. For panels A, and D to G, data are represented as means ± SD of at least 3 independent experiments.

Table S1. List of Illumina sequencing primers used for library amplification and NGS of the primary CRISPR/Cas9 screen

Oligo name	Sequence 5'-3'	Corresponding samples
P5	AAT GAT ACG GCG ACC ACC GAG ATC TAC ACT CTT TCC CTA CAC GAC GCT CTT CCG ATC TTT G TG GAA AGG ACG AAA CAC CG	Forward primer used for the initial amplification of the library and of all the samples of the primary CRISPR/Cas9 screen.
P7	CAA GCA GAA GAC GGC ATA CGA GAT GTG ACT GGA GTT CAG ACG TGT GCT CTT CC GAT CTC CAA TTC CCA CTC CTT TCA AGA CCT	Reverse primer used for the initial amplification of the library.
P71	CAA GCA GAA GAC GGC ATA CGA GAT **ACA TCG** GTG ACT GGA GTT CAG ACG TGT GCT CTT CC GAT CTC CAA TTC CCA CTC CTT TCA AGA CCT	Control "T0"
P72	CAA GCA GAA GAC GGC ATA CGA GAT **TGG TCA** GTG ACT GGA GTT CAG ACG TGT GCT CTT CC GAT CTC CAA TTC CCA CTC CTT TCA AGA CCT	Control "T4w"
P73	CAA GCA GAA GAC GGC ATA CGA GAT **CAC TGT** GTG ACT GGA GTT CAG ACG TGT GCT CTT CC GAT CTC CAA TTC CCA CTC CTT TCA AGA CCT	E2
P74	CAA GCA GAA GAC GGC ATA CGA GAT **ATT GGC** GTG ACT GGA GTT CAG ACG TGT GCT CTT CC GAT CTC CAA TTC CCA CTC CTT TCA AGA CCT	db-cAMP
P75	CAA GCA GAA GAC GGC ATA CGA GAT **GAT CTG** GTG ACT GGA GTT CAG ACG TGT GCT CTT CC GAT CTC CAA TTC CCA CTC CTT TCA AGA CCT	E2 + 4-OHT
P76	CAA GCA GAA GAC GGC ATA CGA GAT **TCA AGT** GTG ACT GGA GTT CAG ACG TGT GCT CTT CC GAT CTC CAA TTC CCA CTC CTT TCA AGA CCT	E2 + ICI
P77	CAA GCA GAA GAC GGC ATA CGA GAT **AAG CTA** GTG ACT GGA GTT CAG ACG TGT GCT CTT CC GAT CTC CAA TTC CCA CTC CTT TCA AGA CCT	E2 + 4-OHT + db-cAMP
P78	CAA GCA GAA GAC GGC ATA CGA GAT **TAC AAG** GTG ACT GGA GTT CAG ACG TGT GCT CTT CC GAT CTC CAA TTC CCA CTC CTT TCA AGA CCT	E2 + ICI + db-cAMP

Barcode sequences in bold are available from the NEBNext Multiplex Oligos for Illumina (Index Primers Set 1) (NEB #E7335S/L).

Underlined sequences correspond to the vector binding sequences.

Table S2. List of specific primer sequences used for real-time RT-qPCR

**Gene**	**Forward 5'-3'**	**Reverse 5'-3'**
ESR1	GCTCTTGGACAGGAACCAGG	AAGATCTCCACCATGCCCTCT
CXCL12	CCCAGGTGCTACACCCTTTT	CAGGAATGGGGCTCCTTCAG
TFF1	CAATTCTGTCTTTCACGGGG	CACCATGGAGAACAAGGTGA
XBP1	CCCTCCAGAACATCTCCCCAT	ACATGACTGGGTCCAAGTTGT
GREB1	GGCAGGACCAGCTTCTGA	CTGTTCCCACCACCTTGG
β-ACTIN	CATGTACGTTGCTATCCAGGC	CTCCTTAATGTCACGCACGAT

Table S3. List of antibodies used in this study

Antigen and/or antibody	Supplier	Catalog no.	Host	Clonality	Dilution
ERα	Bethyl Laboratories	A300-498A-M	Rabbit	Polyclonal	1:500
PAICS	Atlas antibodies	HPA041538	Rabbit	Polyclonal	1:500 for western blotting
					1:25 for tissue staining
Phospho-S305 of ERα	Abnova	PAB12622	Rabbit	Polyclonal	1:300
Phospho-S104/106 of ERα	Formerly available from Bethyl Laboratories) *	N/A	Rabbit	Polyclonal	1:500
CREB1 (48H2)	Cell signaling technology	9197	Rabbit	Monoclonal	1:1000
Phospho-S133 of CREB1 (87G3)	Cell signaling technology	9198	Rabbit	Monoclonal	1:1000
β-actin (13E5)	Cell signaling technology	4970	Rabbit	Monoclonal	1:1000
HSP73 against HSC70	StressMarq	SMC-151	Mouse	Monoclonal	1:2000
Hsp90β (scFv H90-10)	Geneva antibody facility	ABCD_AO870	Mouse	Monoclonal scFV	1:2000
Puromycin (clone 12D10)	Sigma-Aldrich	MABE343	Mouse	Monoclonal	1:22000
Phospho-S2448 of mTOR	Cell Signaling Technology	2971	Rabbit	Polyclonal	1:1000
mTOR (7C10)	Cell Signaling Technology	2983	Rabbit	Monoclonal	1:2000
RPS6	GeneTex	GTX130450	Rabbit	Polyclonal	1:1000

phospho- S235/236 of S6 ribosomal protein (D57.2.2E) XP®	Cell Signaling Technology	4858	Rabbit	Monoclonal	1:1000
4EBP1 (N1C3)	GeneTex	GTX109162	Rabbit	Polyclonal	1:1000
Phospho-S65 of 4E-BP1	GeneTex	GTX133184	Rabbit	Polyclonal	1:1000
phospho-S51 of eIF2α (119A11)	Cell Signaling Technology	3597	Rabbit	Monoclonal	1:1000
eIF2α	Cell Signaling Technology	9722	Rabbit	Polyclonal	1:1000
GFP from mouse IgG1κ (clones 7.1 and 13.1)	Roche	11814460001	Mouse	Monoclonal	1:7500
Mouse IgG (H+L), HRP secondary antibody	Invitrogen	31430	Goat	Polyclonal	1:10000
Rabbit IgG (H+L), HRP secondary antibody	Invitrogen	31460	Goat	Polyclonal	1:10000
Cy3 AffiniPure Anti-Rabbit IgG (H+L), secondary red fluorescent antibody	Jackson Laboratory	711-165-152	Donkey	Polyclonal	1:1000 for tissue staining

* A gift from Brian Rowan (Tulane University School of Medicine, New Orleans).

The following Supplementary Materials are available as separate files:

Data S1. Normalized counts of the sgRNAs of the primary CRISPR/Cas9 knockout screen. Plain text document contains the raw read counts from the two biological replicates that were normalized per million reads and presented side by side.

Data S2. Calculated β-score of sgRNAs of the primary CRISPR/Cas9 knockout screen. Plain text document contains values that indicate the enrichment ($\beta > 0$) or depletion ($\beta < 0$) of sgRNAs in the corresponding treatment groups as compared to the control group at T0.

Data S3. Genes essential for MCF7-V cell survival. Microsoft excel workbook contains the list of 600 genes and the calculated β-scores of their corresponding sgRNAs. These genes were considered essential for survival since their sgRNAs were depleted regardless of the added treatments.

Data S4. Top 58 differentially abundant sgRNAs between the treatment conditions. CSV document contains the list of genes represented with the calculated β-score values of their corresponding sgRNAs. The 15 groups of the hierarchical clustering are indicated.

Data S5. Oligonucleotide sequences used to construct the sgRNAs of the secondary CRISPR/Cas9 screen. Microsoft excel workbook contains the sequences that were selected from the Brunello library based on the off-target score and the consistency of read counts between the two replicates of the primary screen.

Data S6. Human breast tissue microarray. Microsoft excel workbook contains the details of the tissue biopsies, including tissue type, pathology diagnosis, TNM stage, tumor grade, the status of ERα, PR, HER2, and Ki-67, the measured fluorescence intensities corresponding to PAICS, the respective fluorescent area, and the calculated relative fluorescence of PAICS to unit area.

Data S7. Uncropped images of immunoblots. The pdf file contains all uncropped and unadjusted images.

Data S8. Original numerical data. The zipped archive contains Microsoft Excel workbooks with all numerical data used to generate the figures.
