# Supplementary material for: CRISPR-Cas9 screen reveals a role of purine synthesis for estrogen receptor α activity and tamoxifen resistance of breast cancer cells
Source: Sci Adv. 2023 May 12;9(19):eadd3685. doi: 10.1126/sciadv.add3685 (PMC10181187; doi:10.1126/sciadv.add3685)

Figure 3

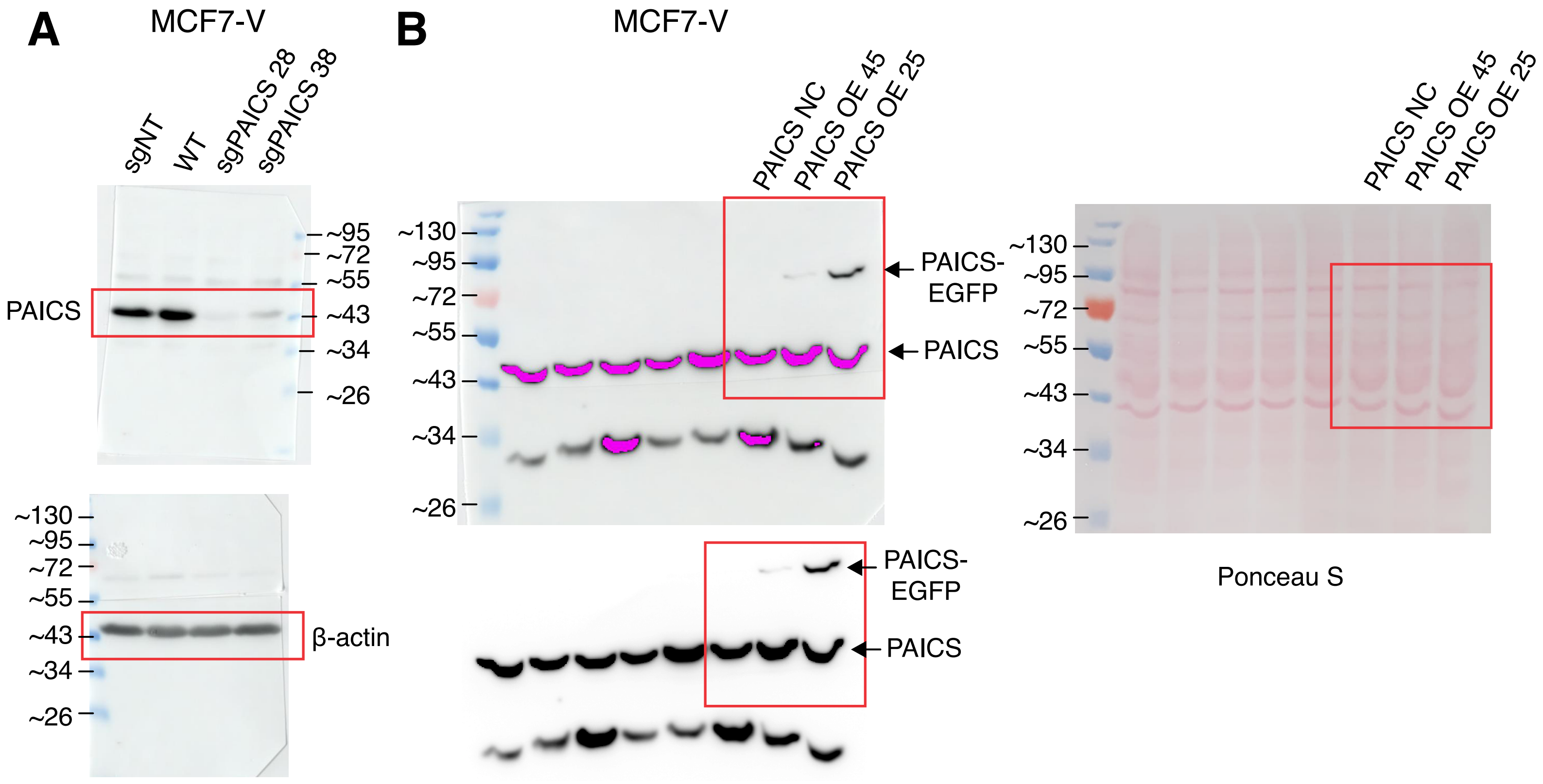

Figure 4

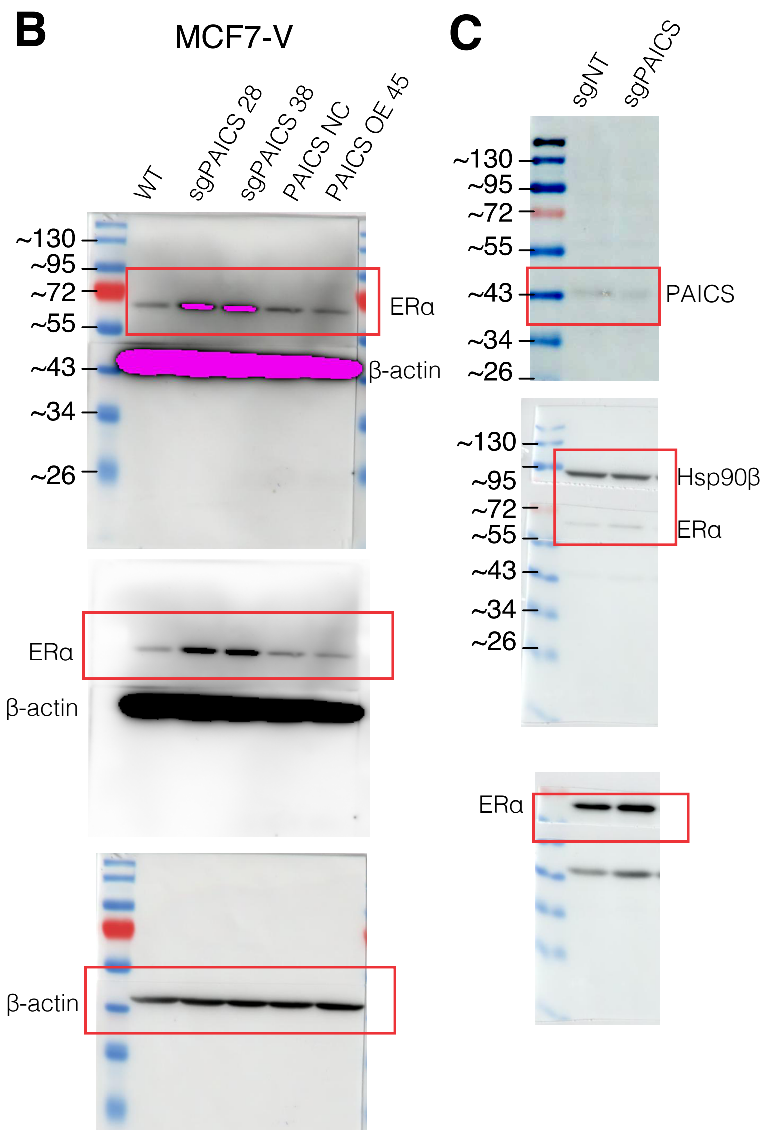

Figure 5

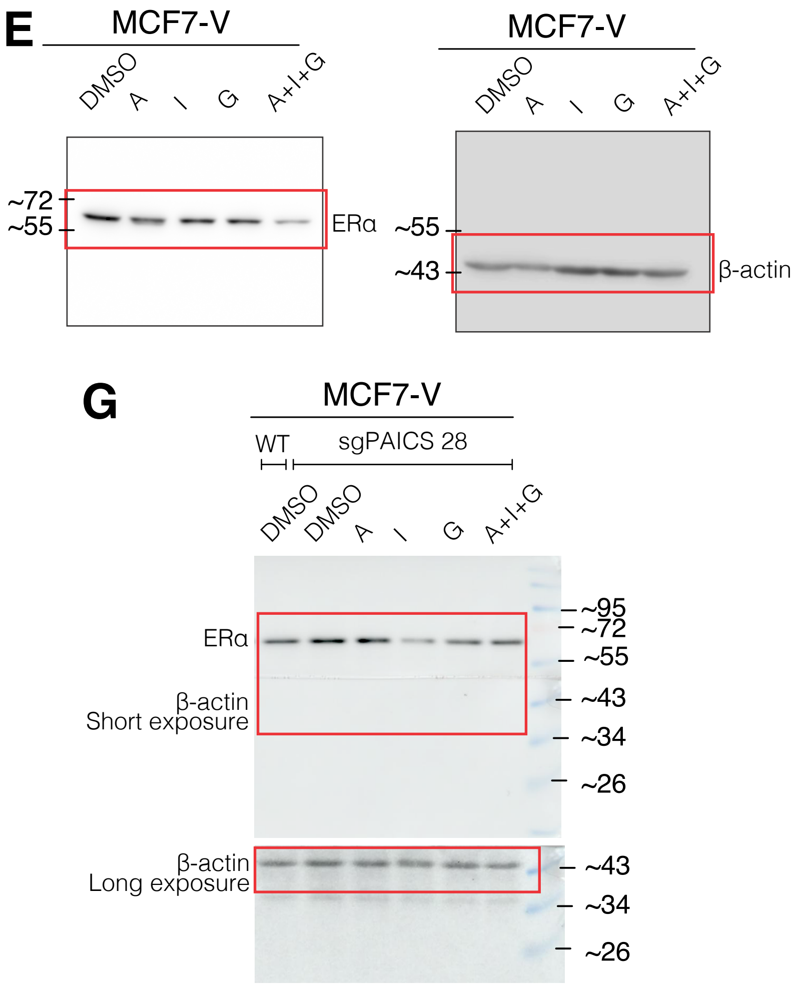

Figure 6

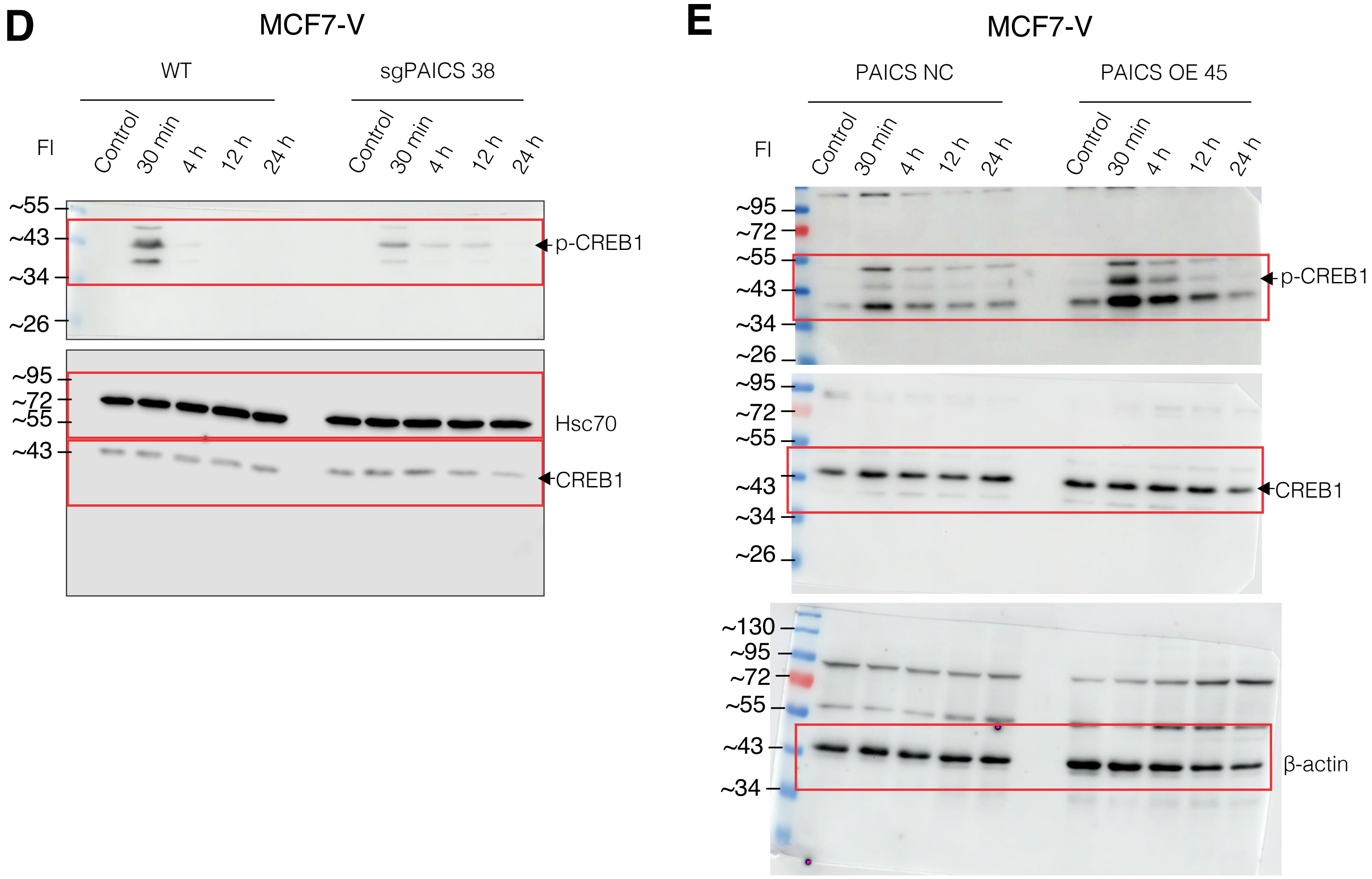

Figure 7

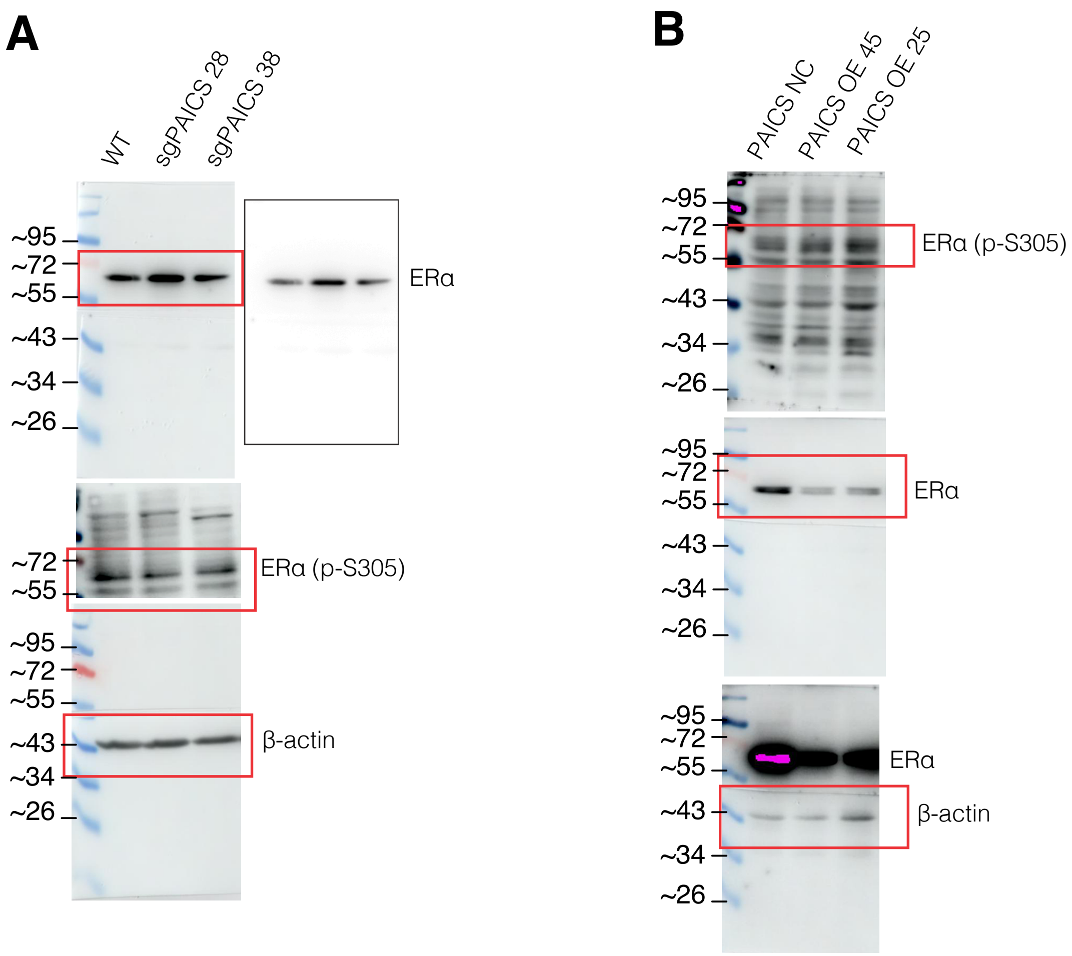
A
WT
sgPAICS 28
sgPAICS 38
~95
~72
~55
~43
~34
~26
ERα
ERα (p-S305)
β-actin
B
PAICS NC
PAICS OE 45
PAICS OE 25
ERα (p-S305)
ERα
β-actin

Figure 8

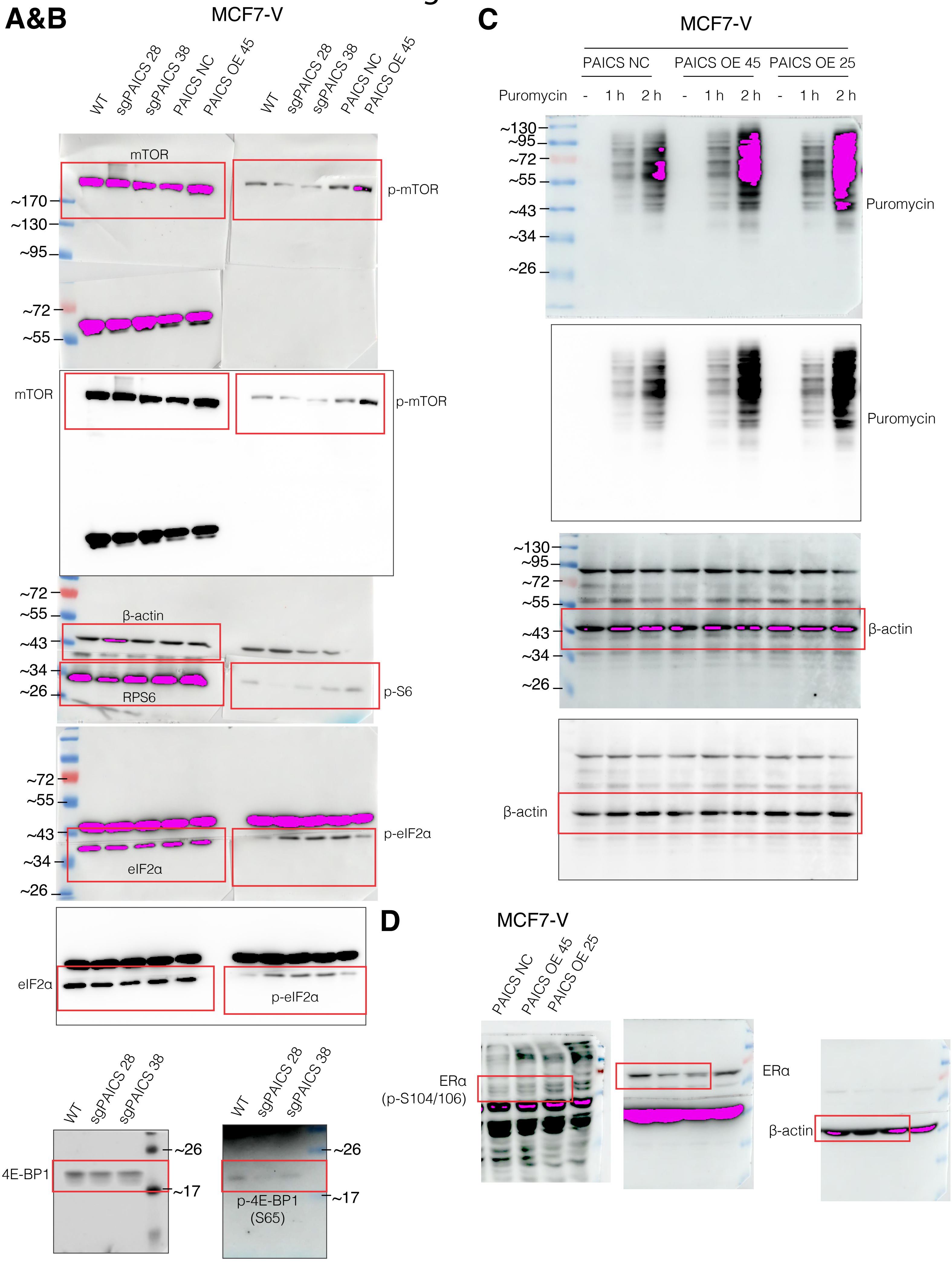
A&B
MCF7-V
WT
sgPAICS 28
sgPAICS 38
PAICS NC
PAICS OE 45
mTOR
p-mTOR
~170
~130
~95
~72
~55
β-actin
RPS6
p-S6
~43
~34
~26
p-eIF2α
eIF2α
4E-BP1
p-4E-BP1
(S65)
~17
C
PAICS NC
PAICS OE 45
PAICS OE 25
Puromycin
- 1 h 2 h - 1 h 2 h - 1 h 2 h
D
ERα
(p-S104/106)

Figure 9

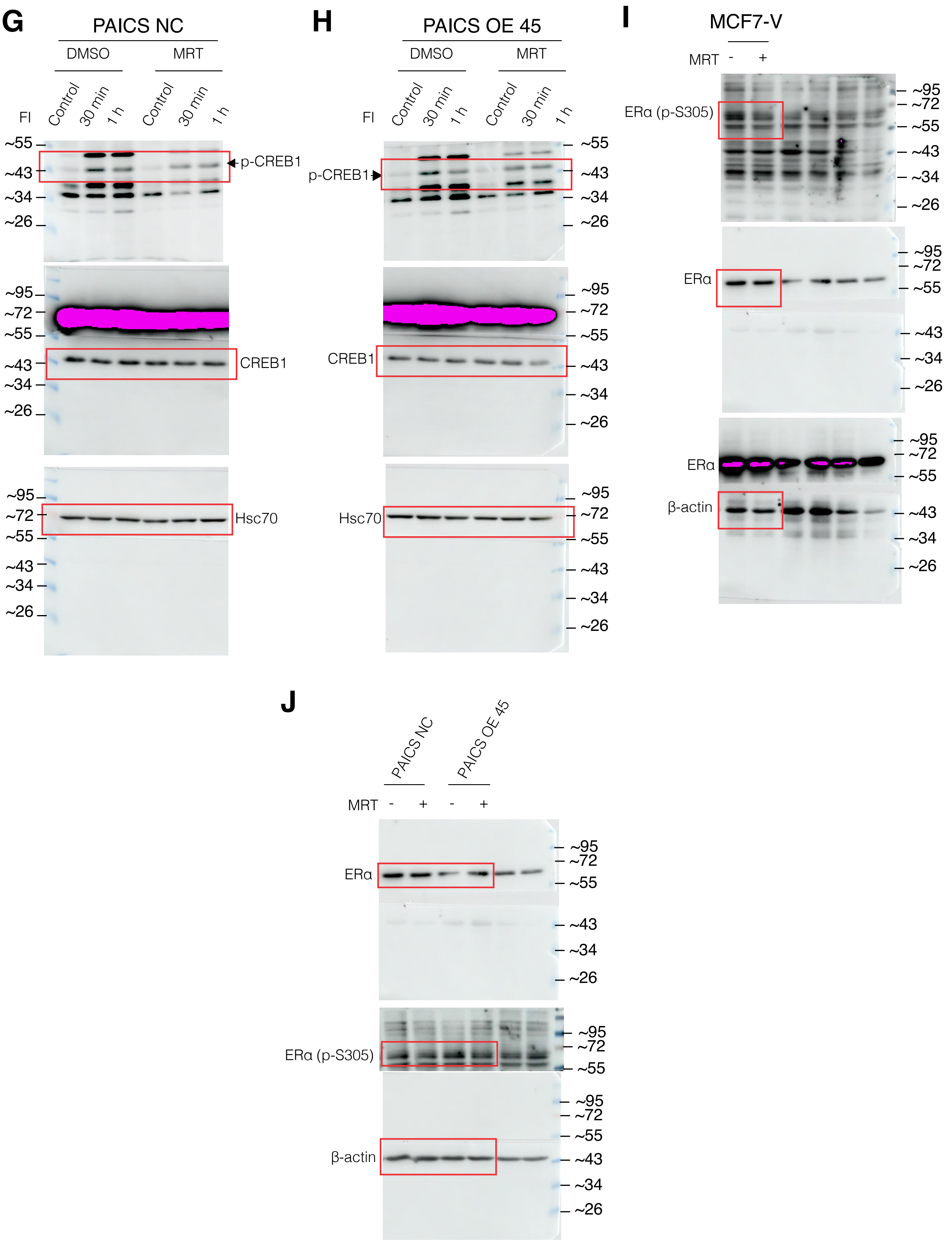
G
PAICS NC
DMSO
MRT
FI
Control
30 min
1 h
Control
30 min
1 h
~55
~43
~34
~26
p-CREB1
~95
~72
~55
~43
~34
~26
CREB1
~95
~72
~55
~43
~34
~26
Hsc70
H
PAICS OE 45
DMSO
MRT
FI
Control
30 min
1 h
Control
30 min
1 h
p-CREB1
~55
~43
~34
~26
~95
~72
~55
CREB1
~43
~34
~26
Hsc70
~95
~72
~55
~43
~34
~26
I
MCF7-V
MRT - +
ERα (p-S305)
~95
~72
~55
~43
~34
~26
ERα
~95
~72
~55
~43
~34
~26
ERα
~95
~72
~55
β-actin
~43
~34
~26
J
PAICS NC
PAICS OE 45
MRT - + - +
ERα
~95
~72
~55
~43
~34
~26
ERα (p-S305)
~95
~72
~55
β-actin
~95
~72
~55
~43
~34
~26

Figure 9

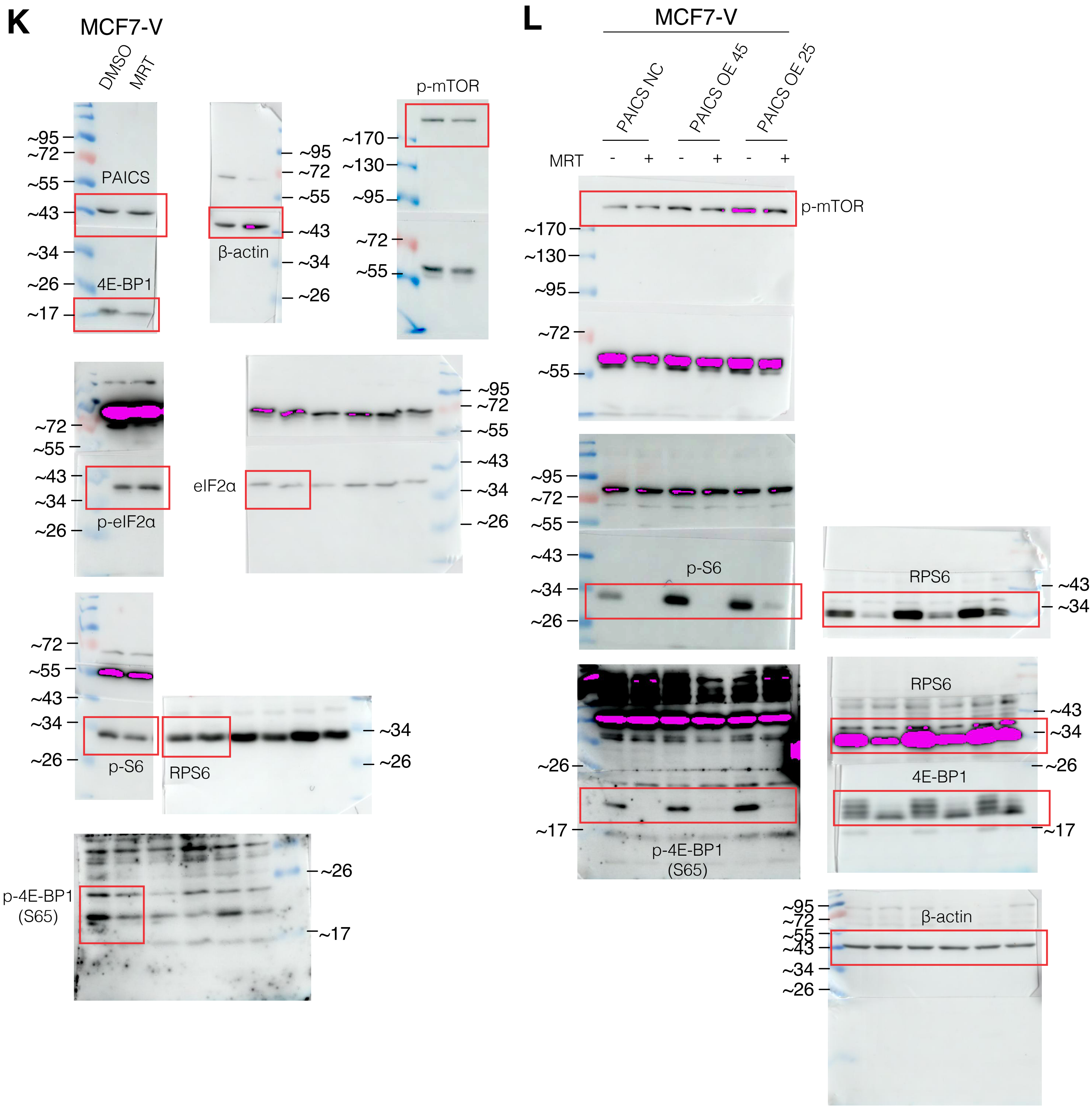

K
MCF7-V
DMSO
MRT
PAICS
4E-BP1
β-actin
p-mTOR
p-eIF2α
eIF2α
p-S6
RPS6
p-4E-BP1
(S65)
L
MCF7-V
PAICS NC
PAICS OE 45
PAICS OE 25
MRT
p-mTOR
p-S6
RPS6
p-4E-BP1
(S65)
4E-BP1
β-actin

Supplementary figure 2

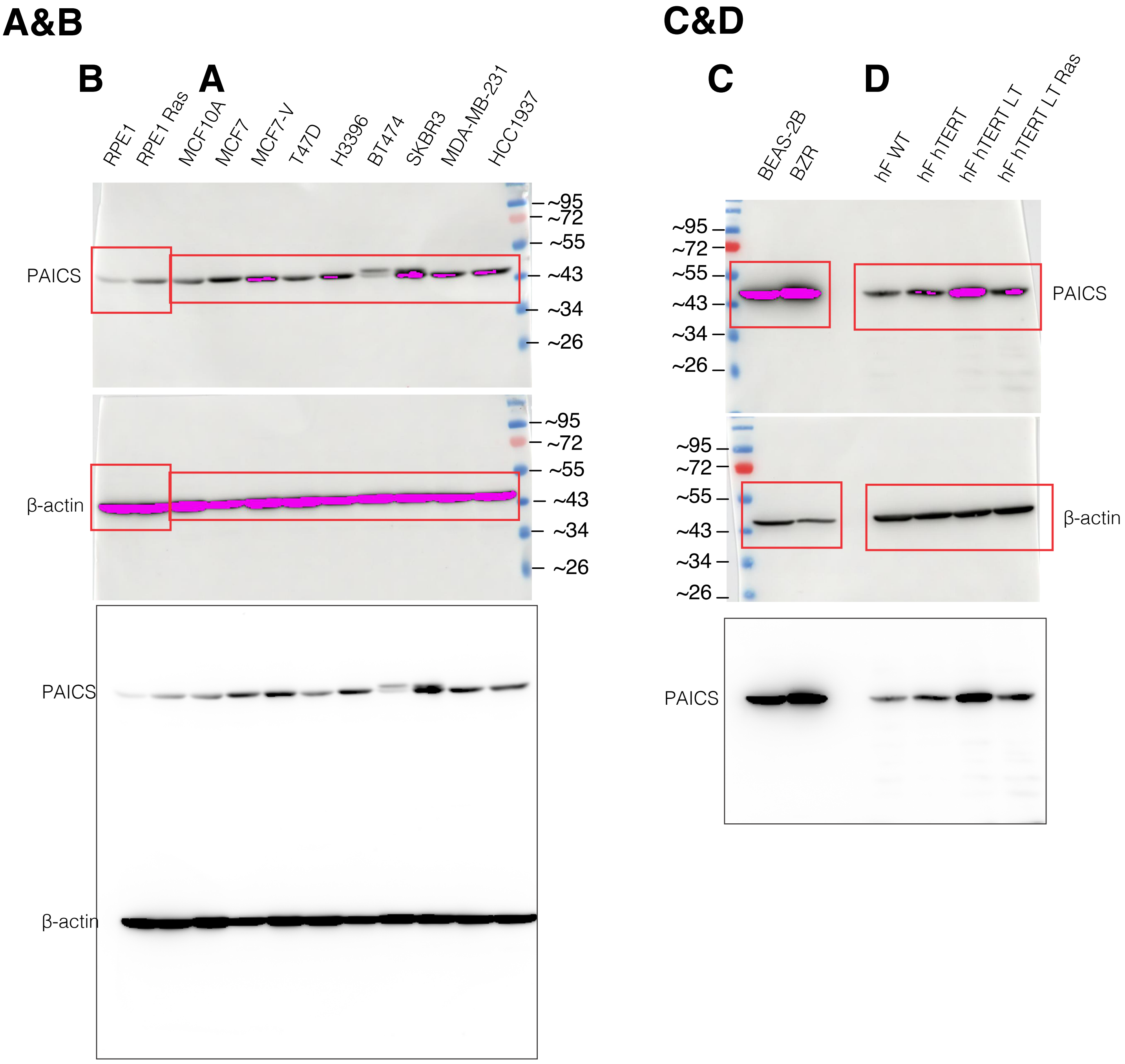

Supplementary figure 5

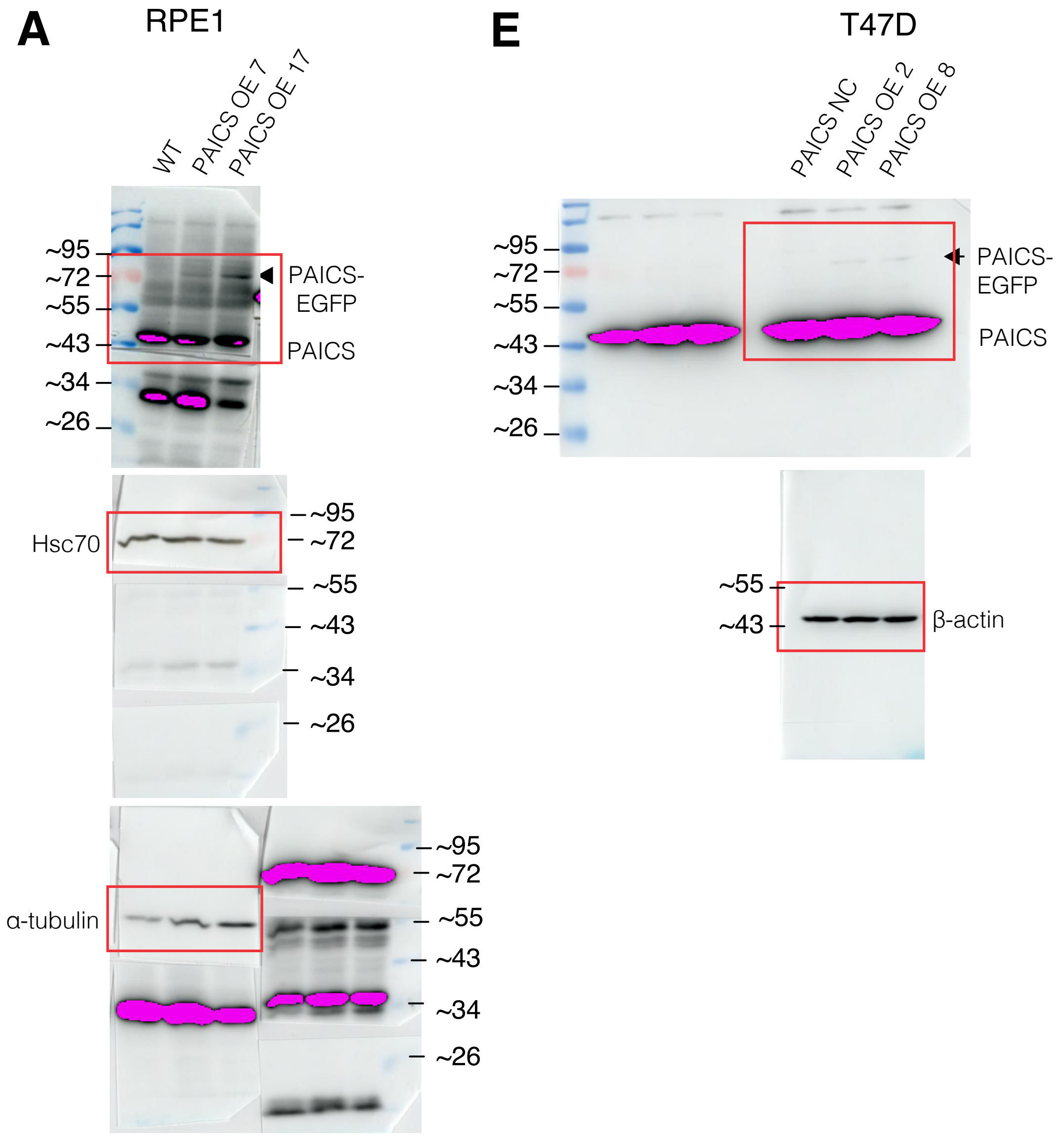

Supplementary figure 7

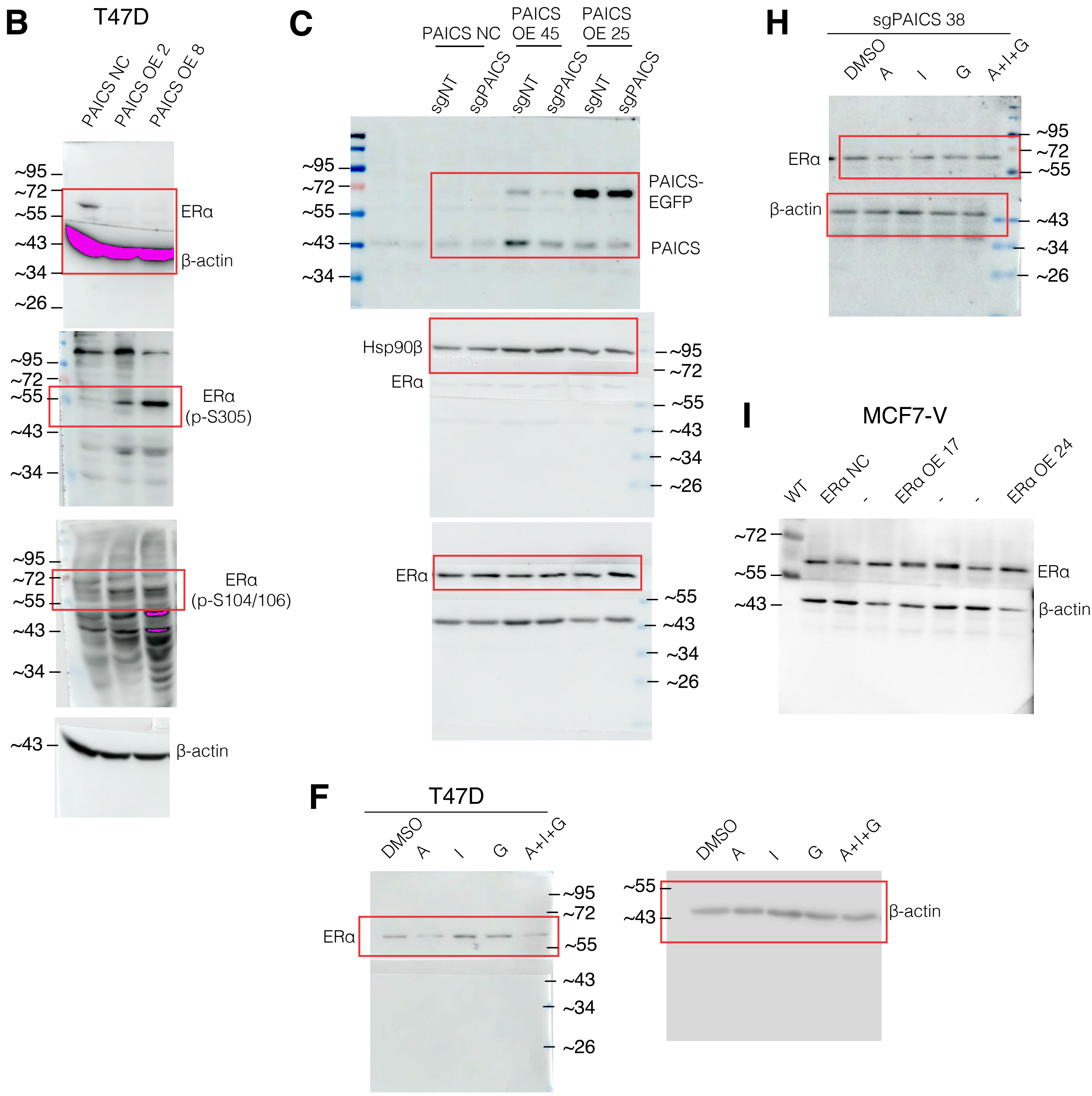

Supplementary figure 8

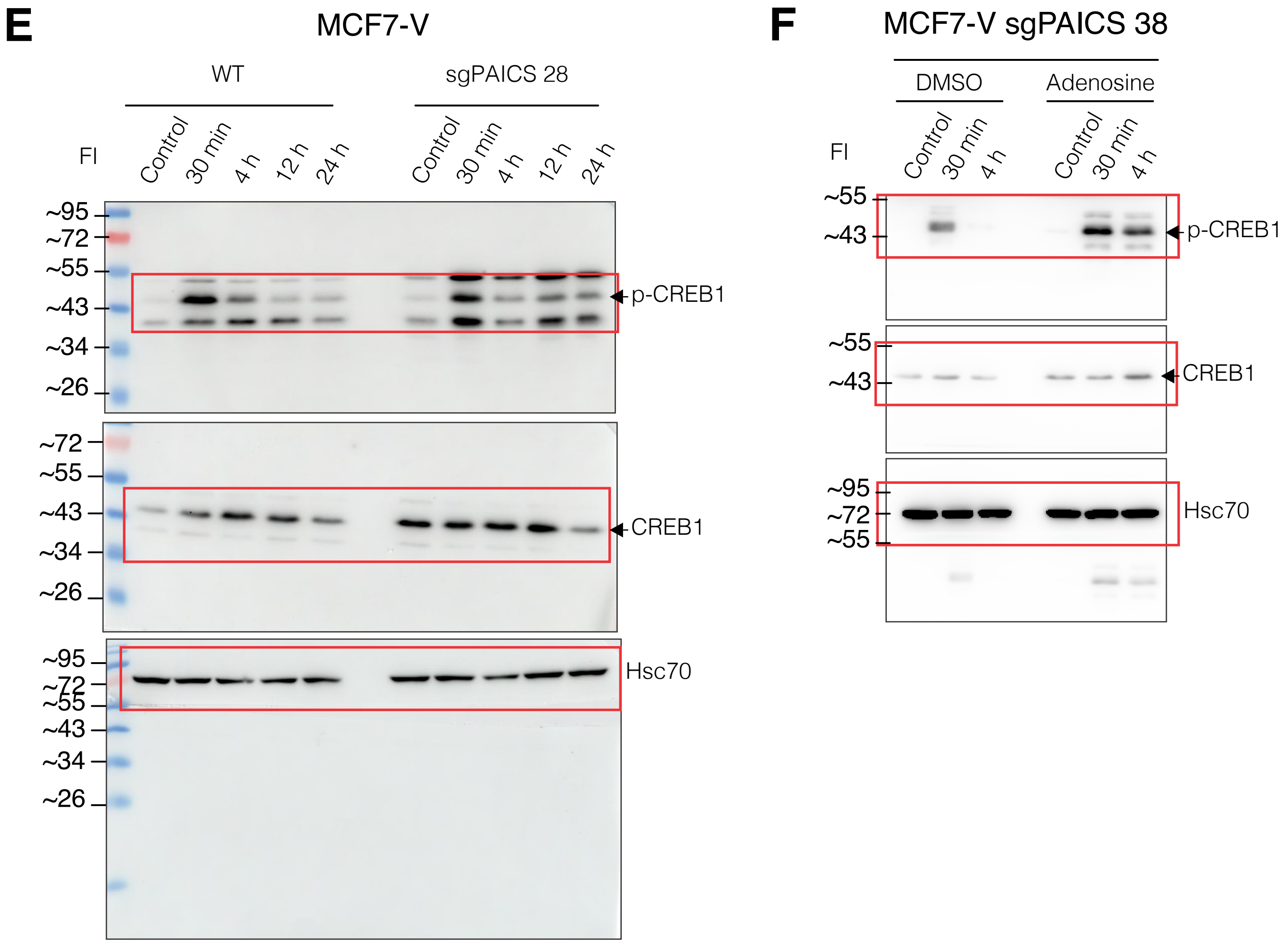

Supplementary figure 9

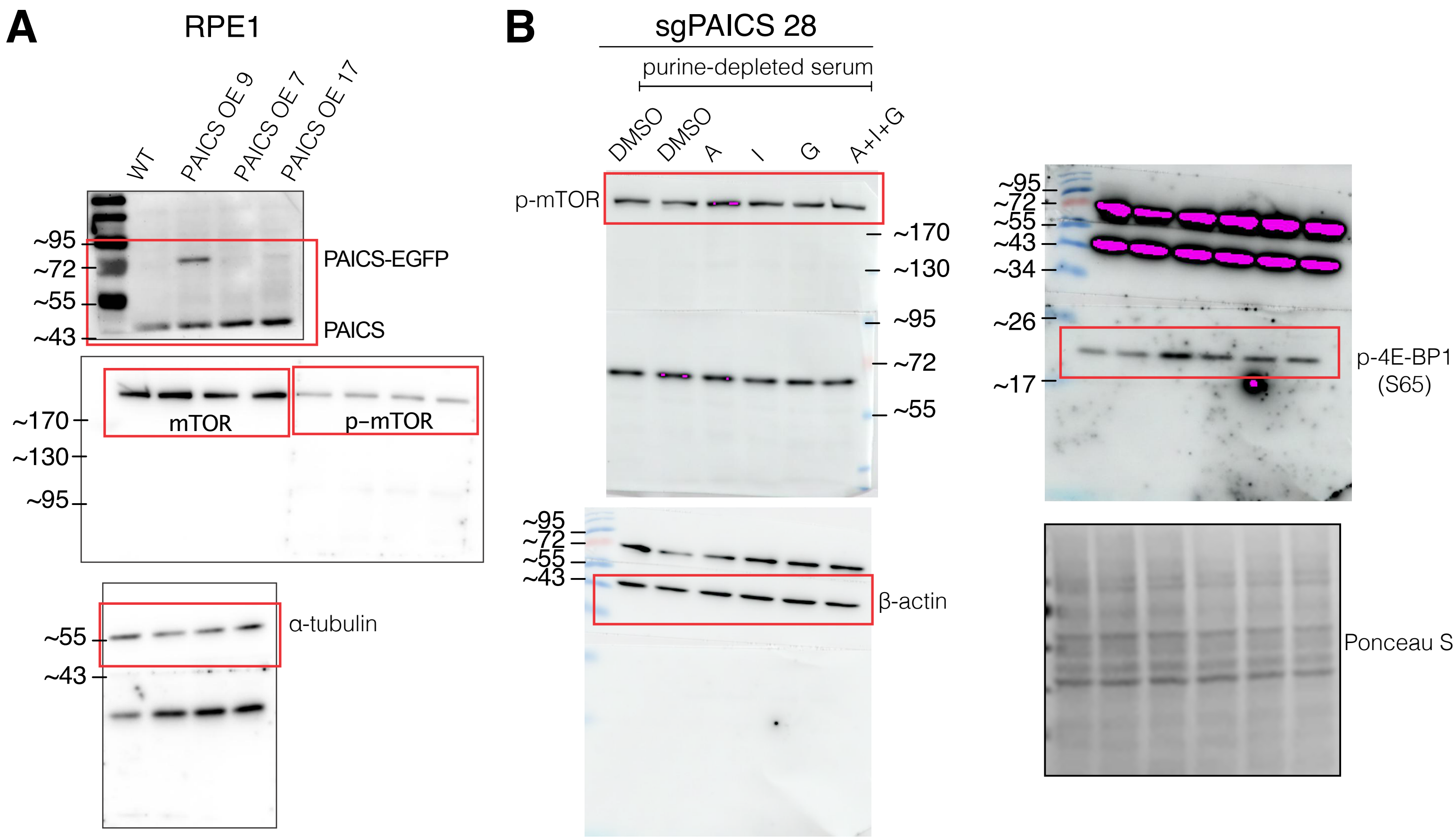

Supplementary figure 10

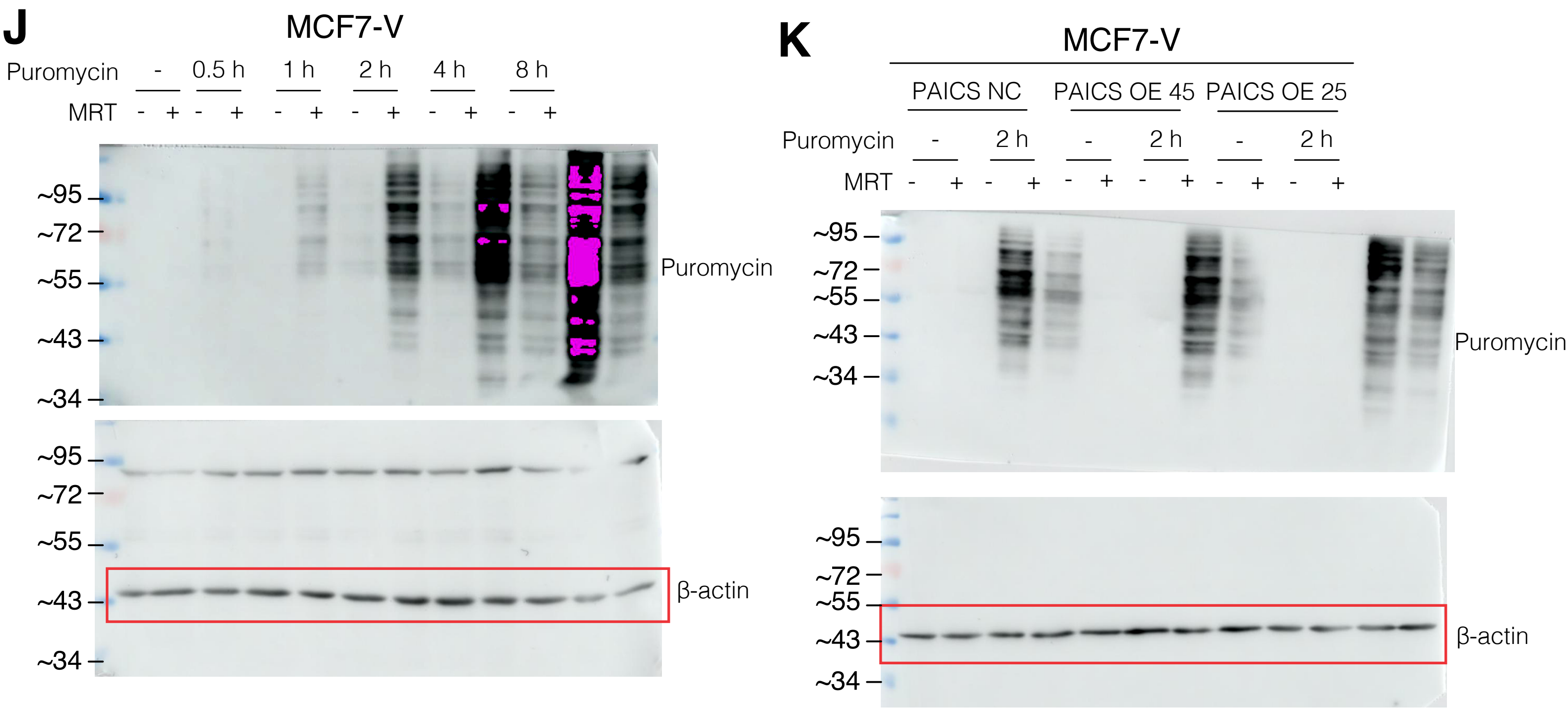

Supplement: Supplementary file 2 — Data S1 to S8 [file sciadv.add3685_data_s1_to_s8.zip › add3685_Data_S7.pdf]
